# Supplementary figures and images for: HNF4α-TET2-FBP1 axis contributes to gluconeogenesis and type 2 diabetes
Source: eLife. 2025 Jun 3;13:RP103663. doi: 10.7554/eLife.103663 (PMC12133150; doi:10.7554/eLife.103663)

Figure. 1C

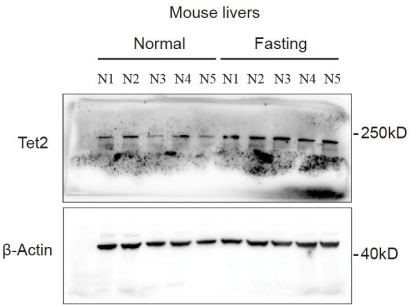

Figure. 1D

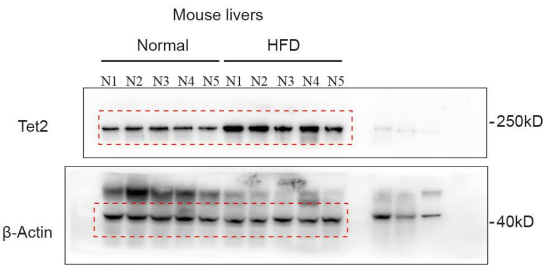

Figure. 1E

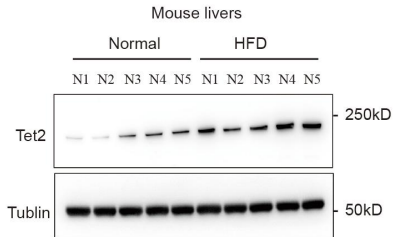

Supplement: Figure 1—source data 1. [file elife-103663-fig1-data1.pdf]

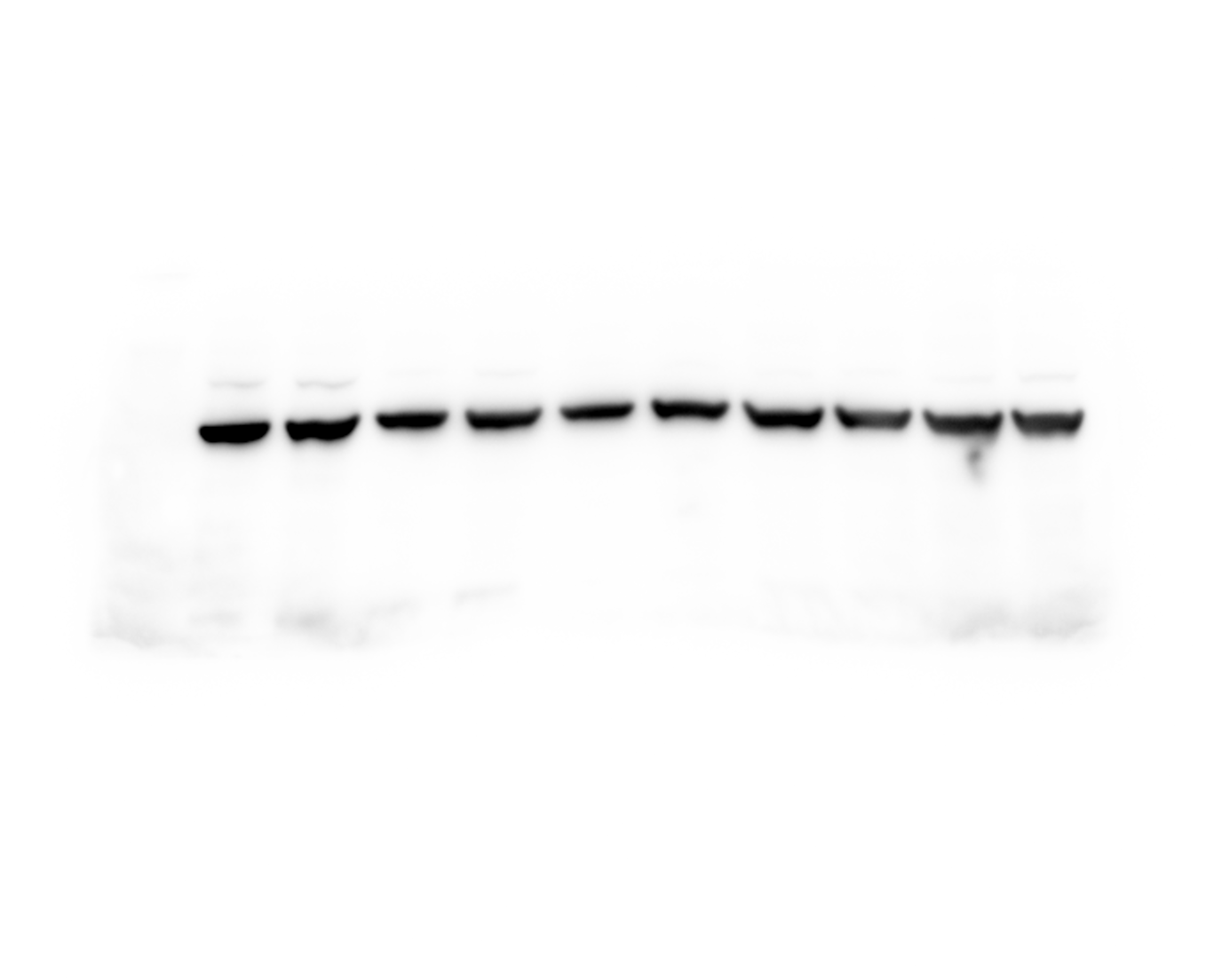

Supplement: Figure 1—source data 2. [file elife-103663-fig1-data2.zip › Figure 1/Figure 1C/1C ACTIN.Tif]

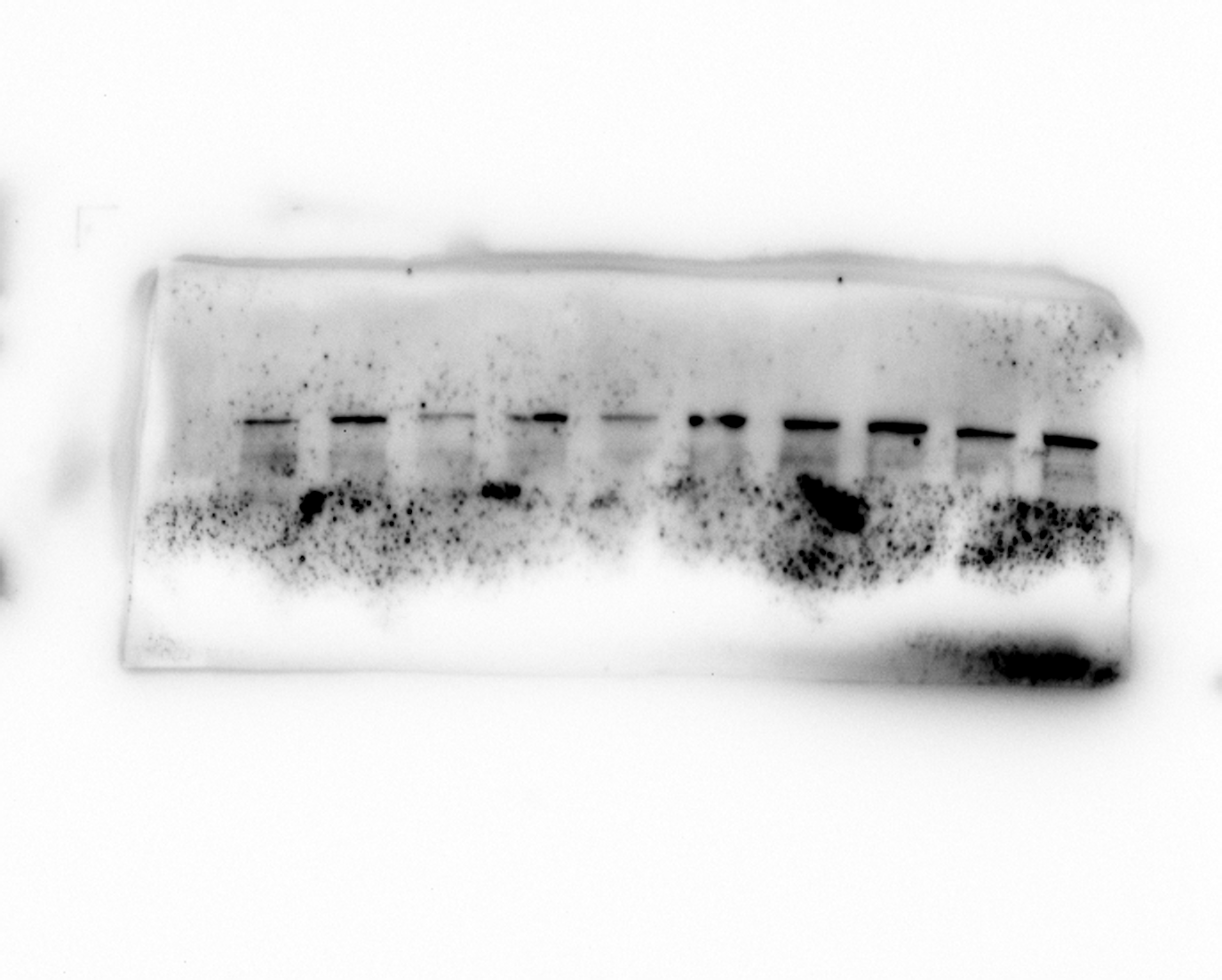

Supplement: Figure 1—source data 2. [file elife-103663-fig1-data2.zip › Figure 1/Figure 1C/1C TET2.Tif]

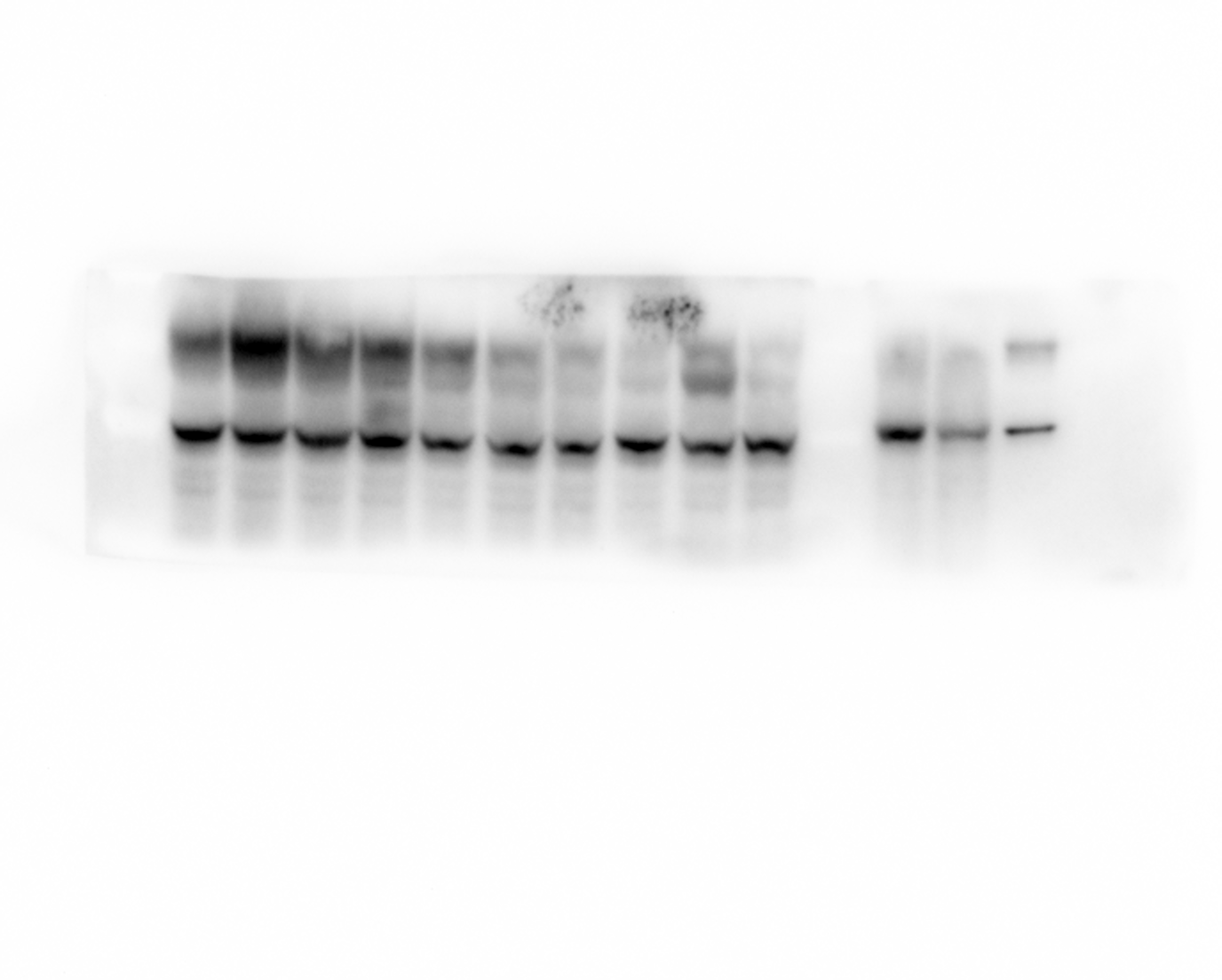

Supplement: Figure 1—source data 2. [file elife-103663-fig1-data2.zip › Figure 1/Figure 1D/1D ACTIN.Tif]

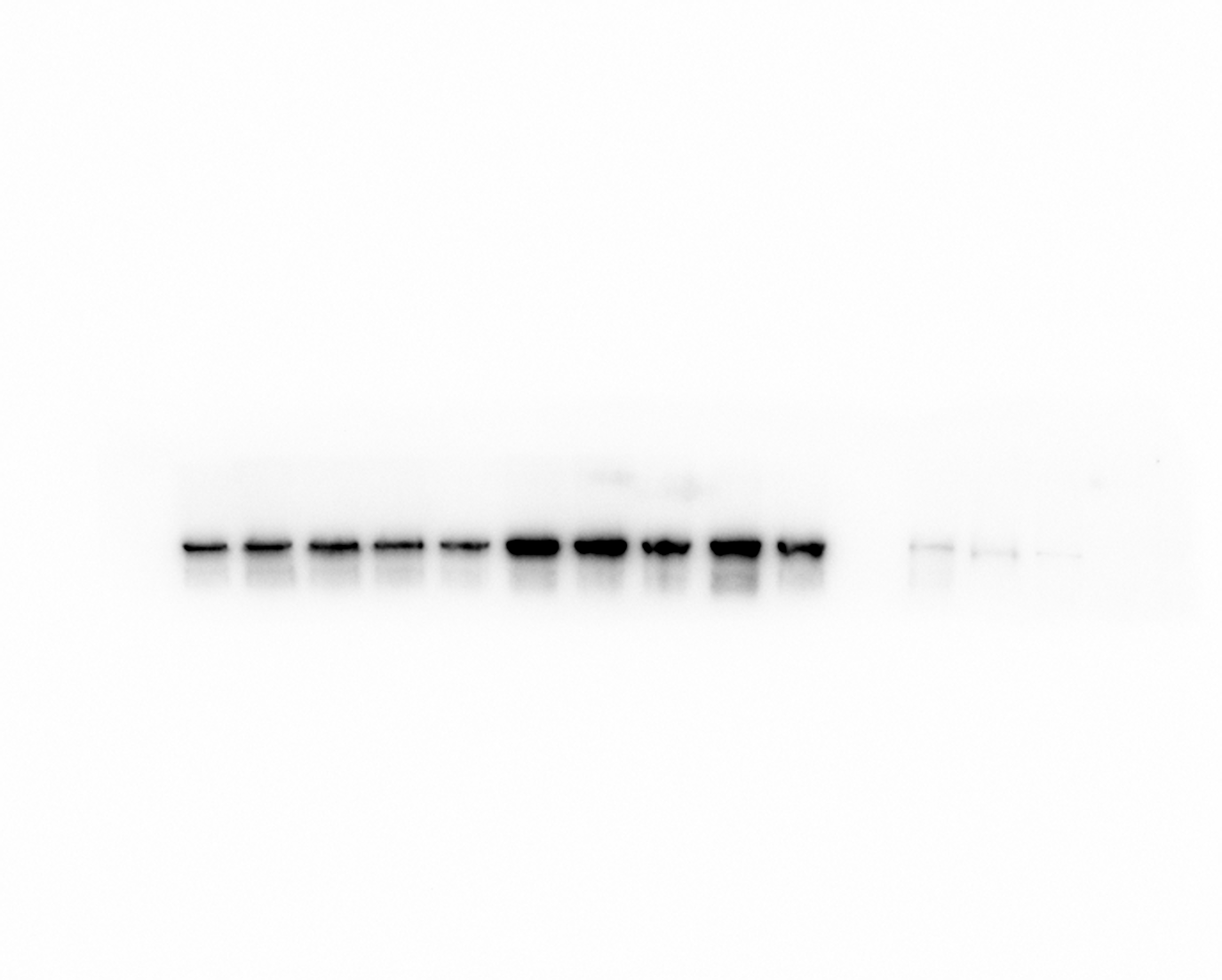

Supplement: Figure 1—source data 2. [file elife-103663-fig1-data2.zip › Figure 1/Figure 1D/1D TET2.Tif]

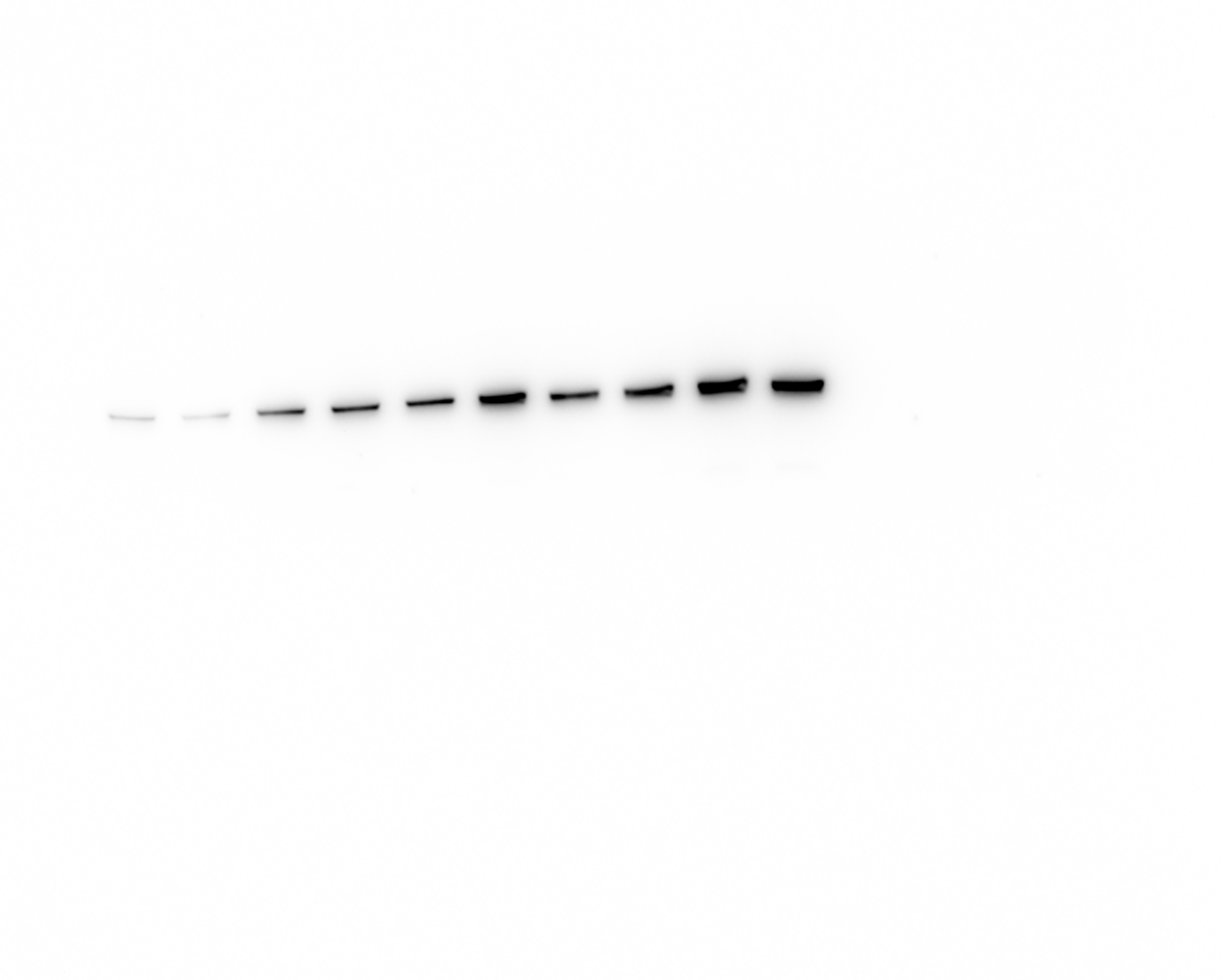

Supplement: Figure 1—source data 2. [file elife-103663-fig1-data2.zip › Figure 1/Figure 1E/1E TET2.Tif]

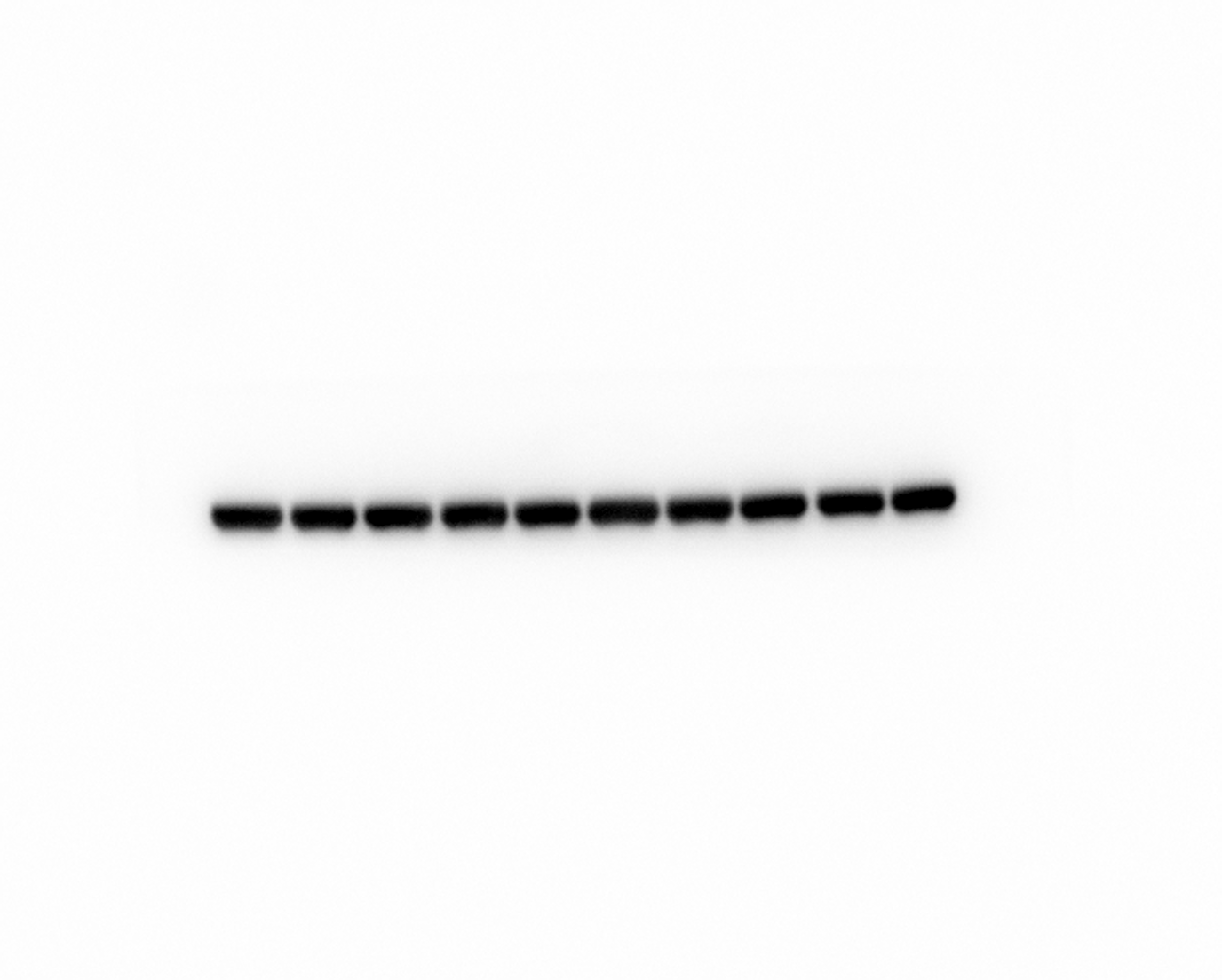

Supplement: Figure 1—source data 2. [file elife-103663-fig1-data2.zip › Figure 1/Figure 1E/1E TUBULIN.Tif]

Figure. 3C

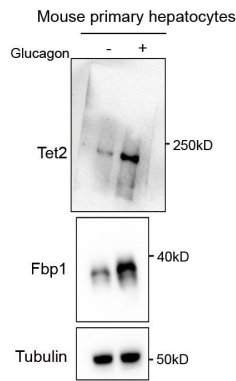

Figure. 3J

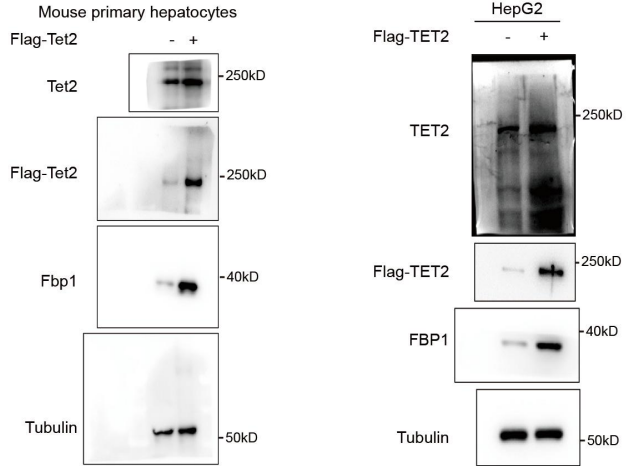

Figure. 3L

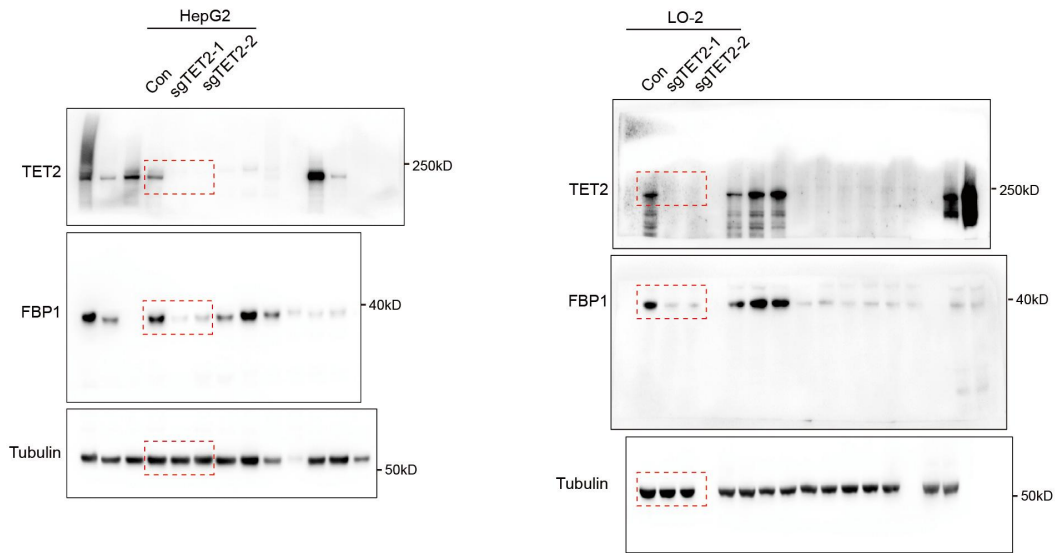

Figure. 3M

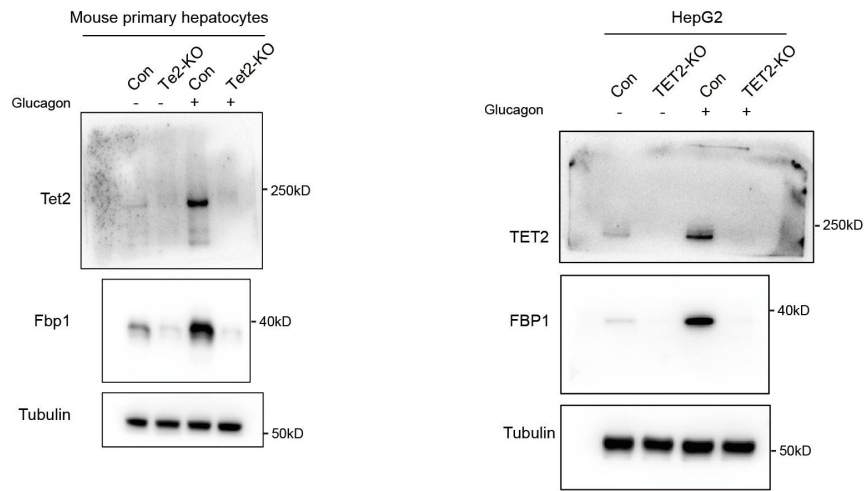

Supplement: Figure 3—source data 1. [file elife-103663-fig3-data1.pdf]

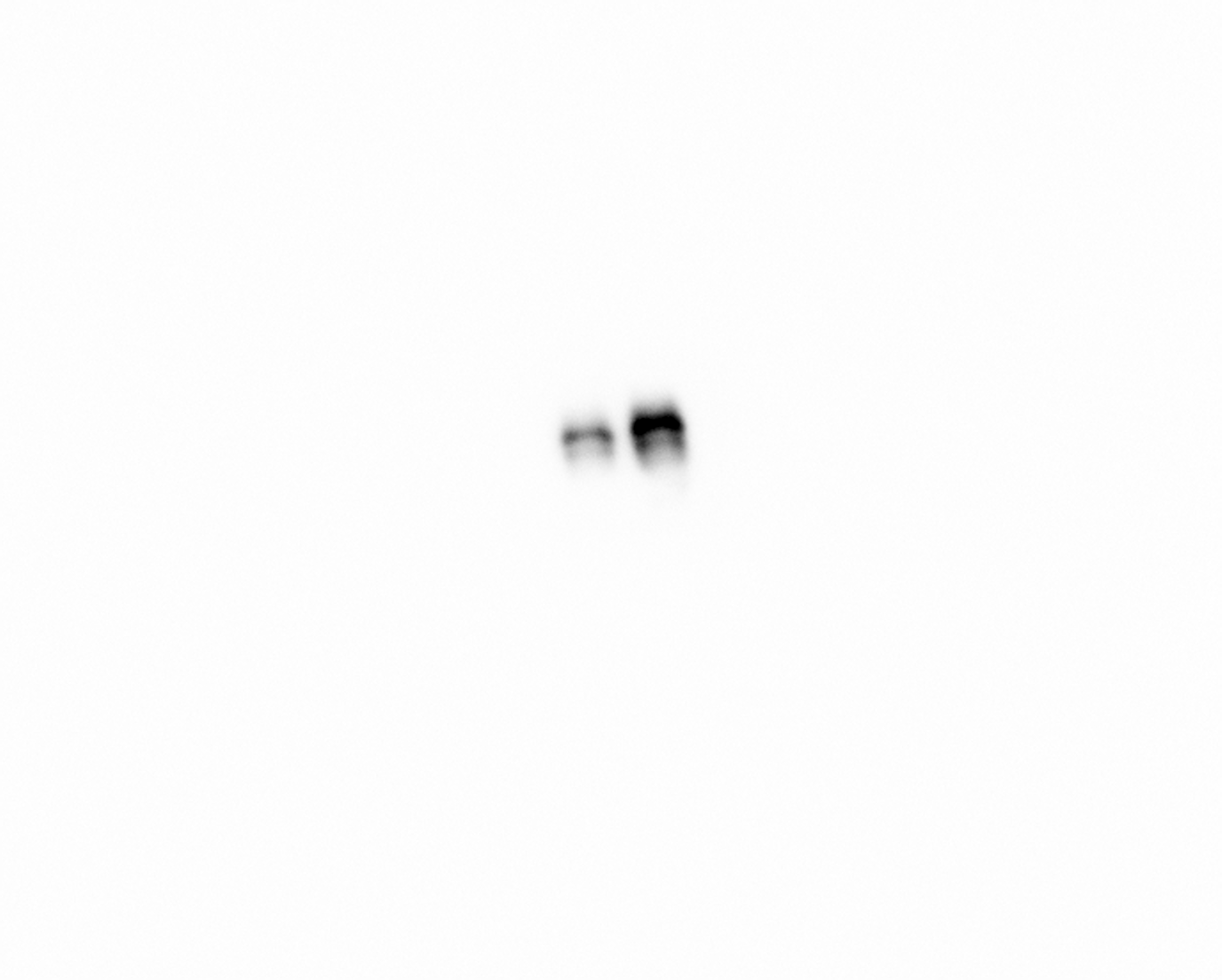

Supplement: Figure 3—source data 2. [file elife-103663-fig3-data2.zip › Figure 3/Figure 3C/Fbp1.Tif]

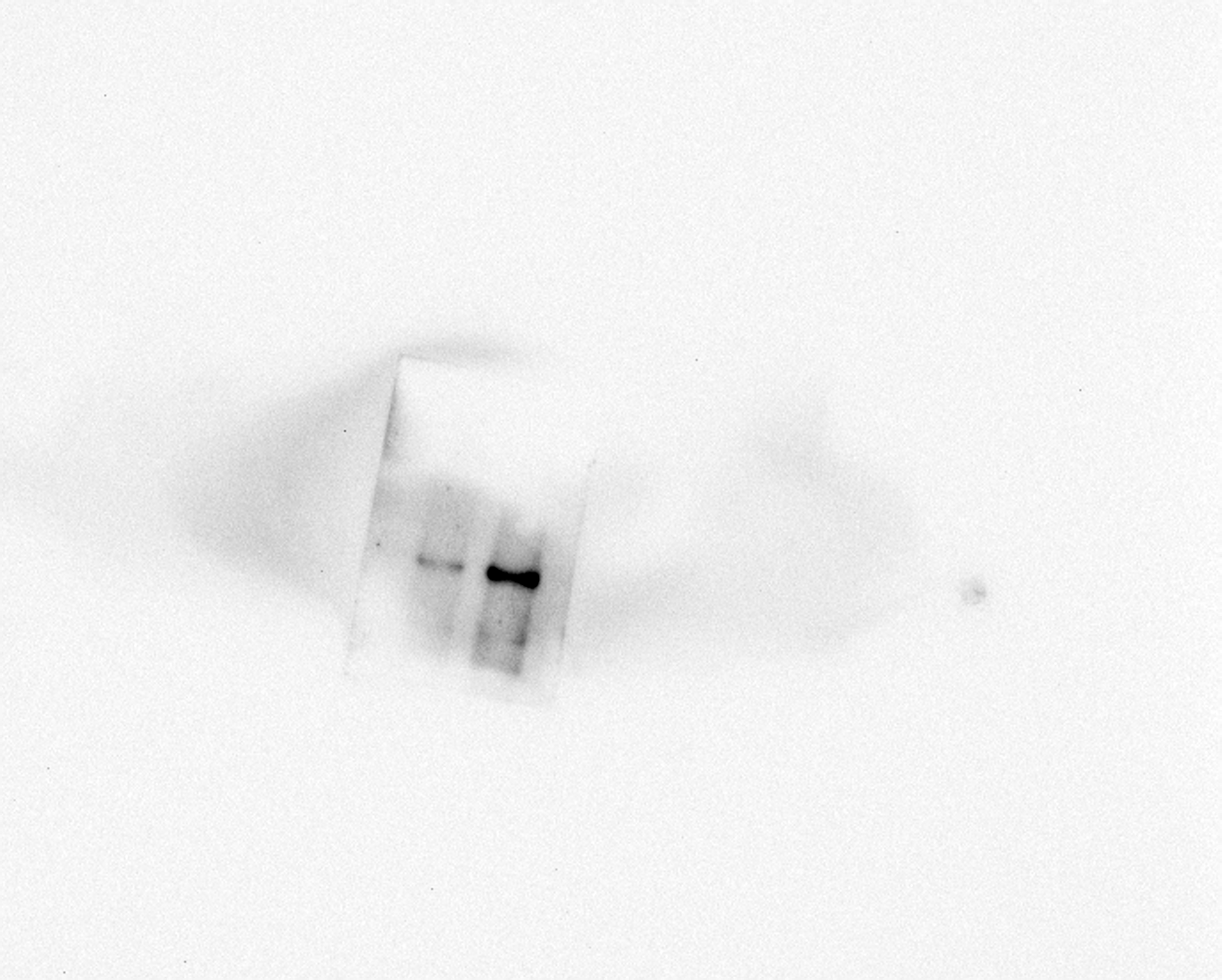

Supplement: Figure 3—source data 2. [file elife-103663-fig3-data2.zip › Figure 3/Figure 3C/Tet2.Tif]

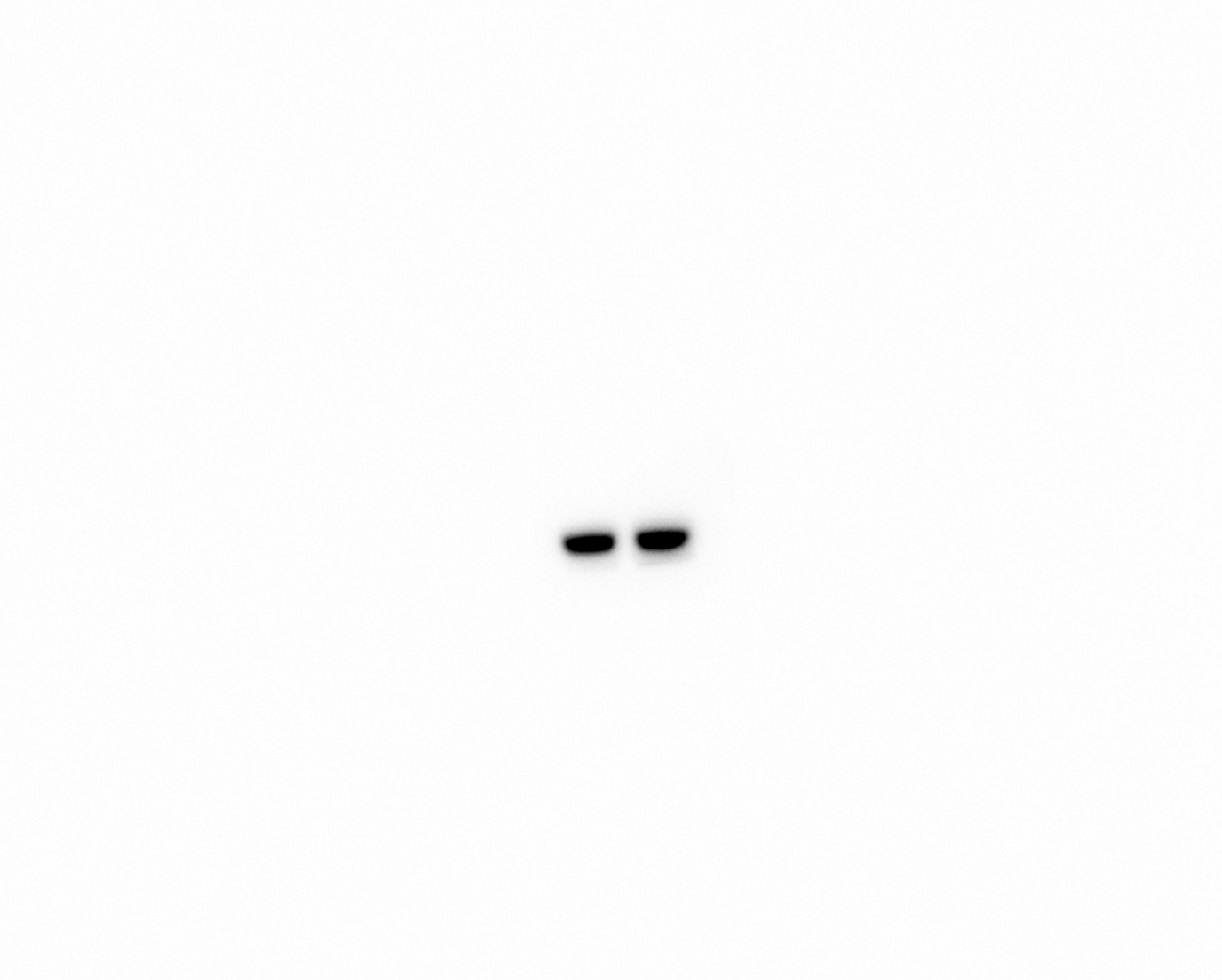

Supplement: Figure 3—source data 2. [file elife-103663-fig3-data2.zip › Figure 3/Figure 3C/Tublin.Tif]

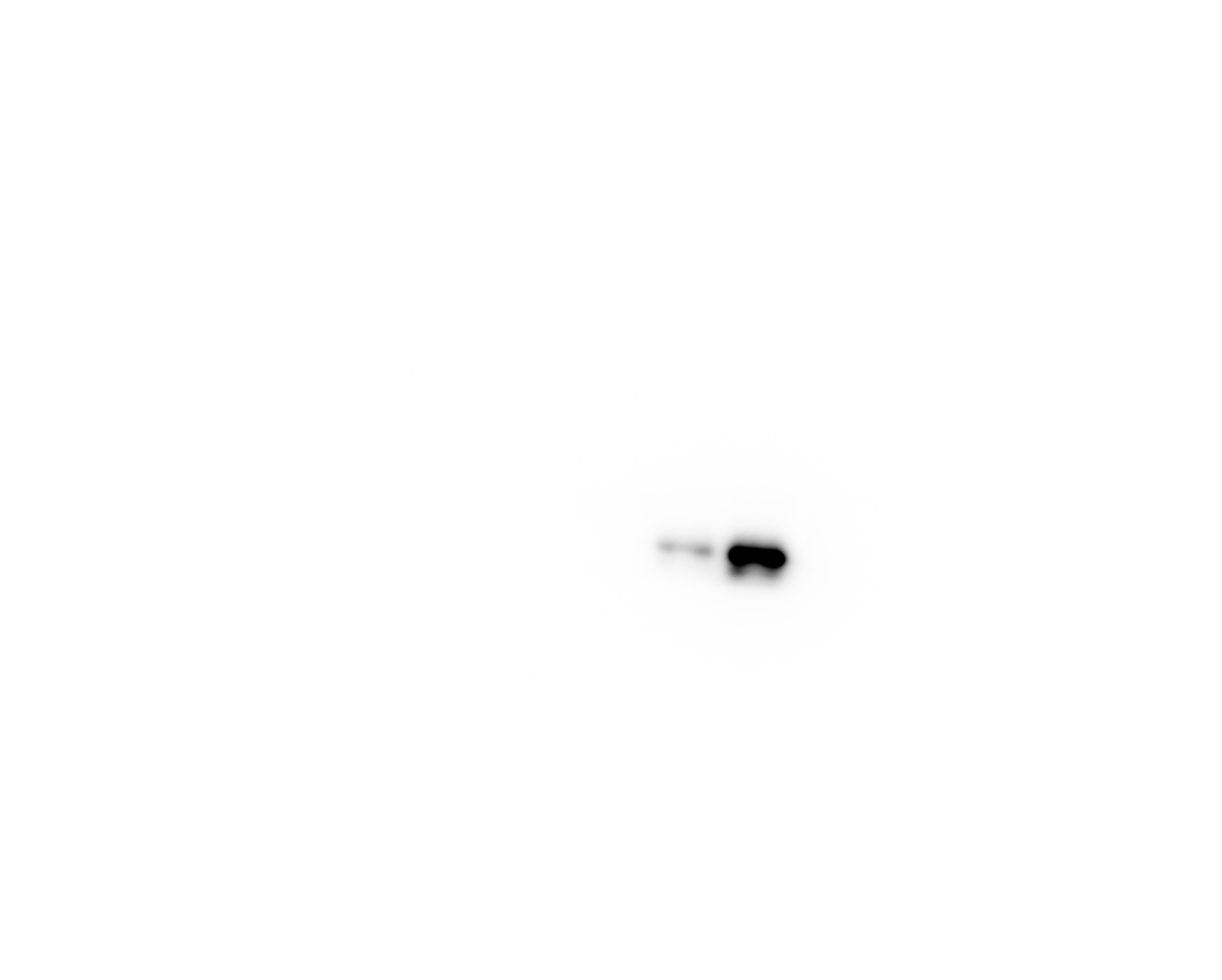

Supplement: Figure 3—source data 2. [file elife-103663-fig3-data2.zip › Figure 3/Figure 3J/Fbp1-left.Tif]

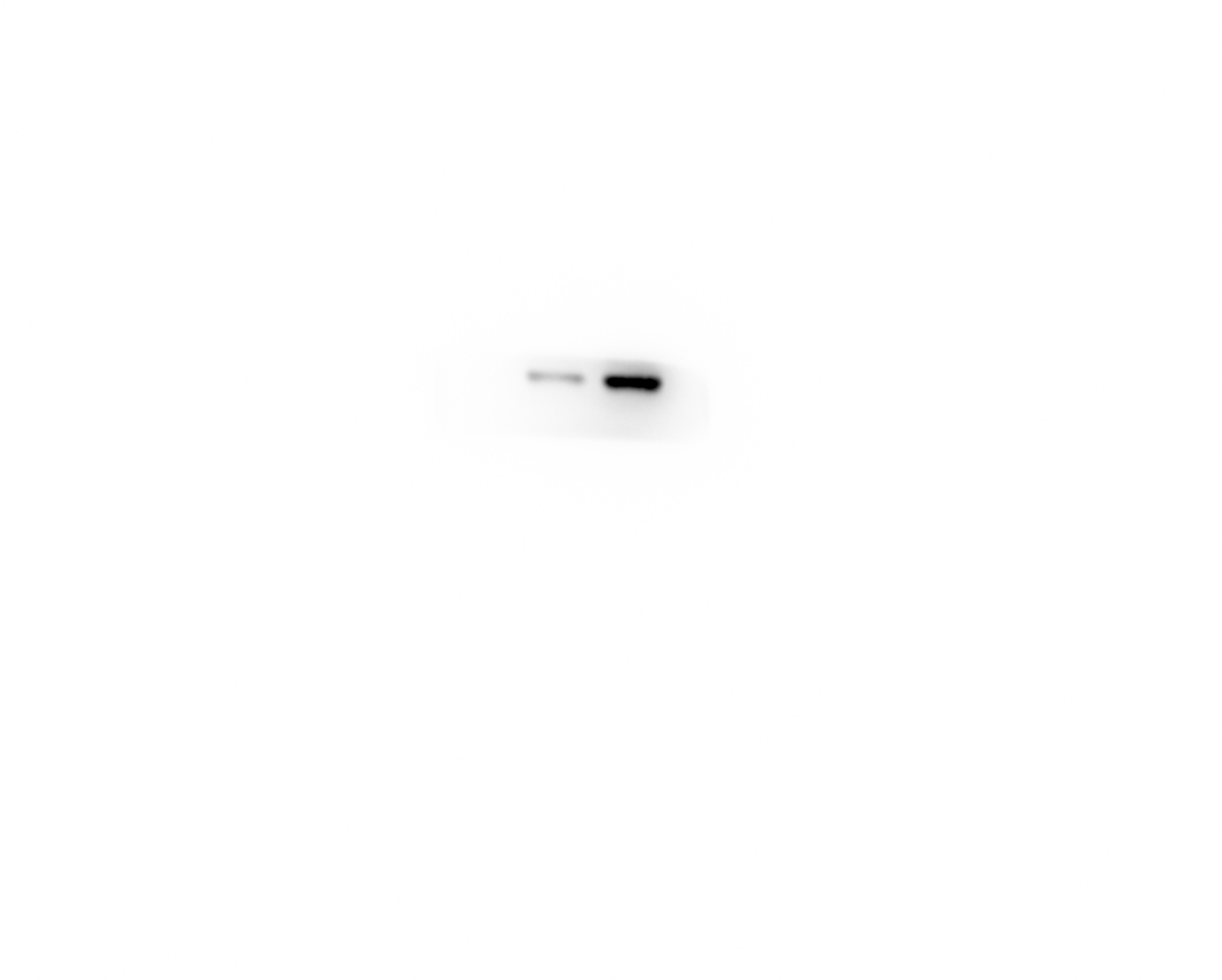

Supplement: Figure 3—source data 2. [file elife-103663-fig3-data2.zip › Figure 3/Figure 3J/Fbp1-right.Tif]

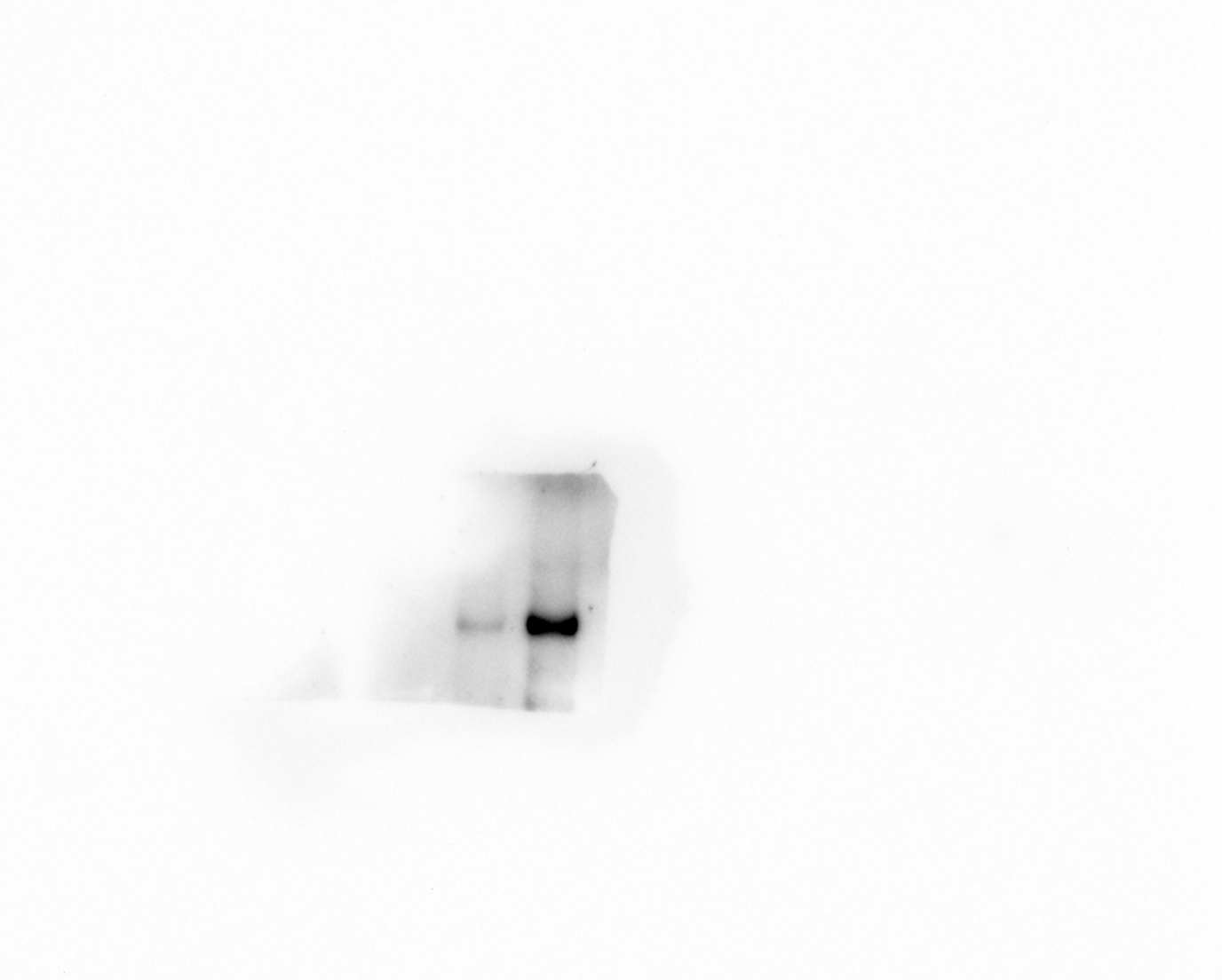

Supplement: Figure 3—source data 2. [file elife-103663-fig3-data2.zip › Figure 3/Figure 3J/Flag-Tet2-left.Tif]

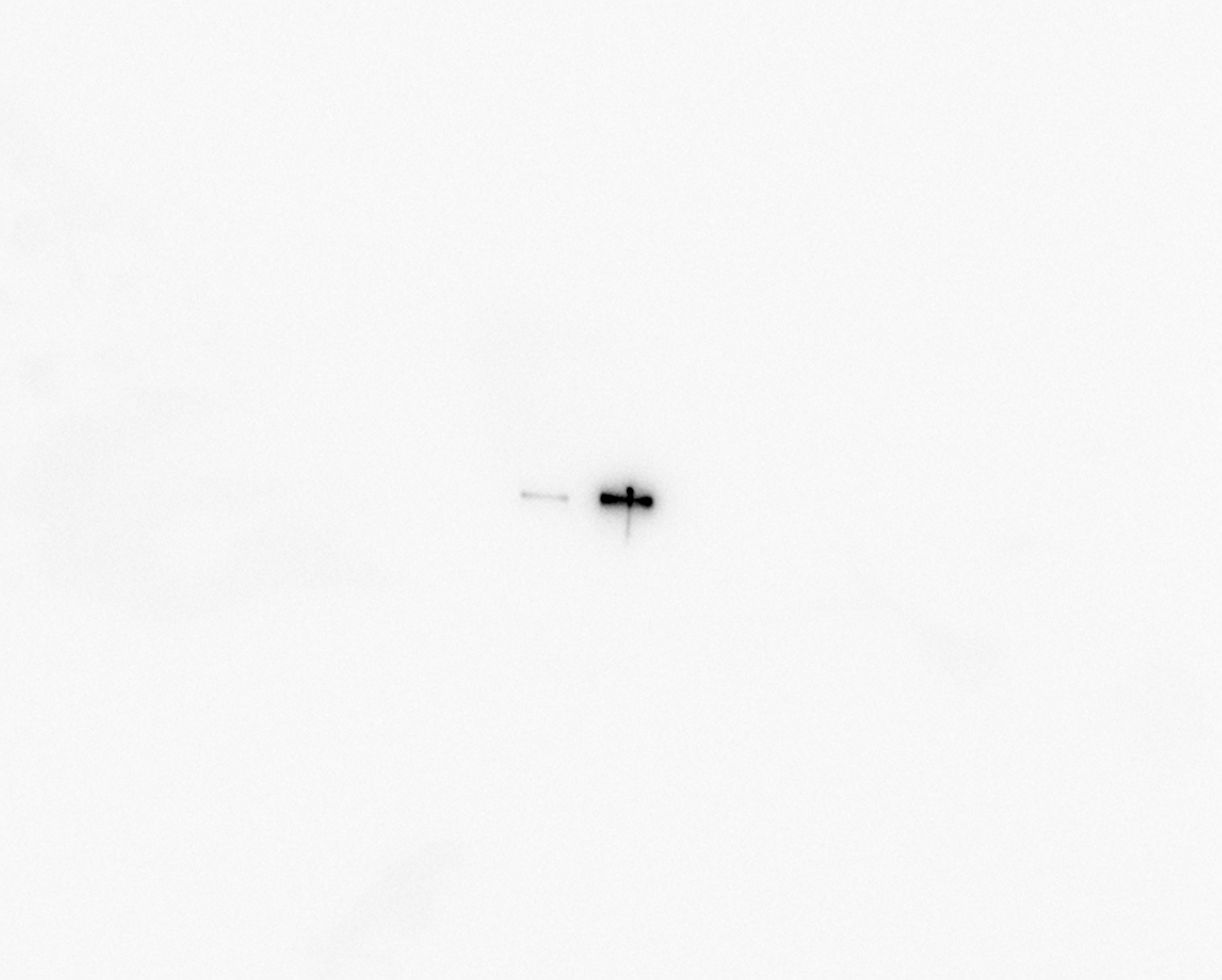

Supplement: Figure 3—source data 2. [file elife-103663-fig3-data2.zip › Figure 3/Figure 3J/Flag-Tet2-right.tif]

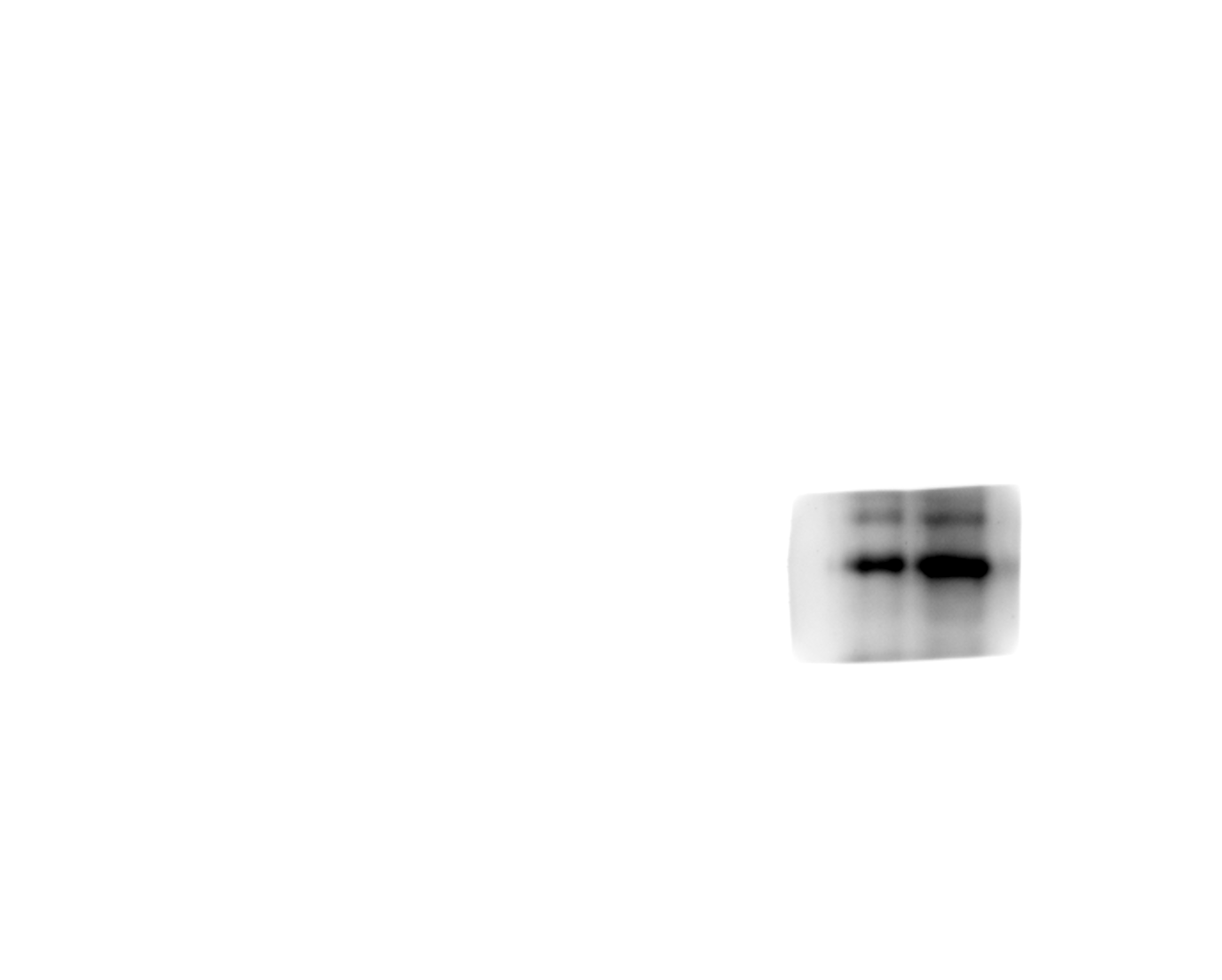

Supplement: Figure 3—source data 2. [file elife-103663-fig3-data2.zip › Figure 3/Figure 3J/Tet2-left.Tif]

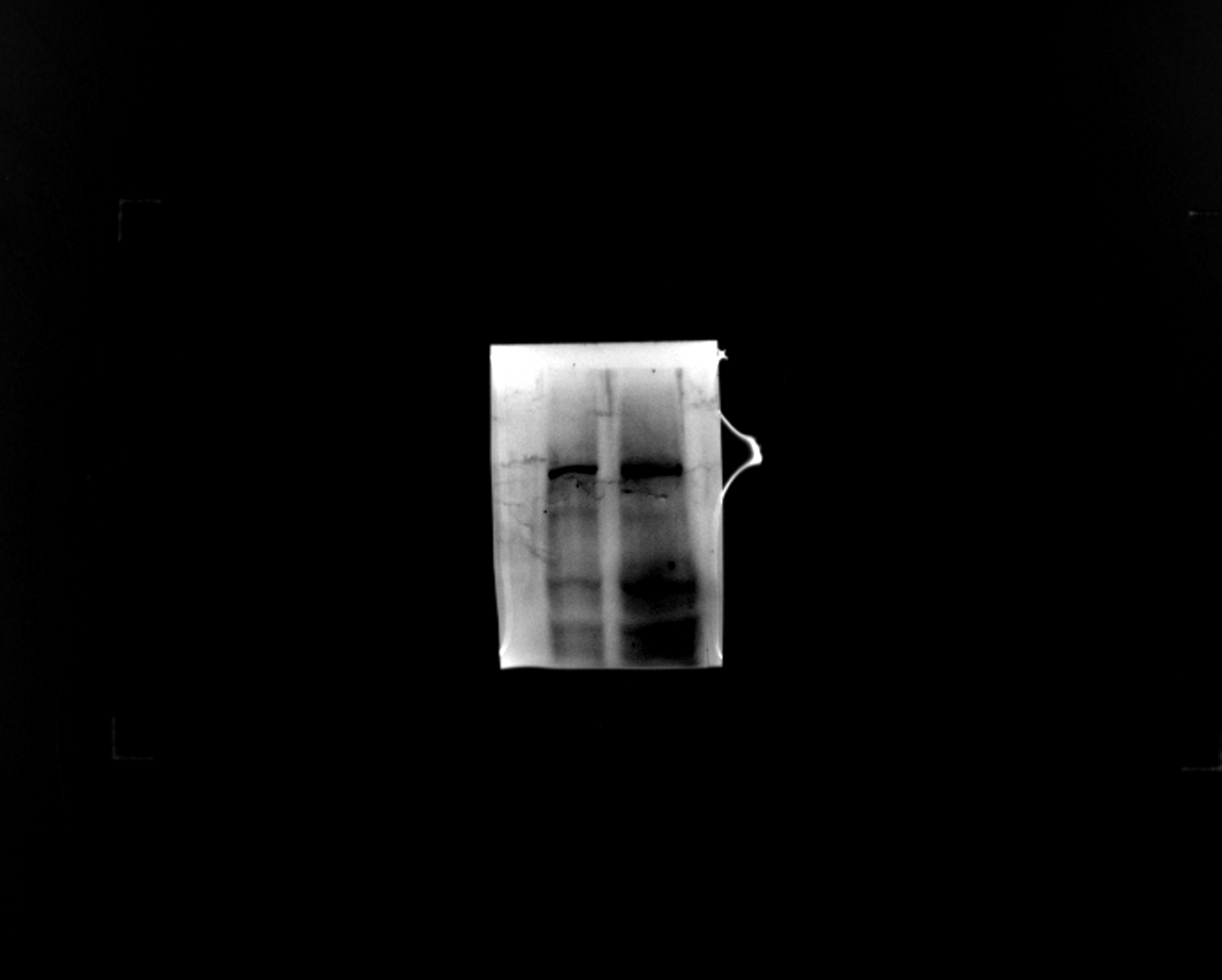

Supplement: Figure 3—source data 2. [file elife-103663-fig3-data2.zip › Figure 3/Figure 3J/Tet2-right.Tif]

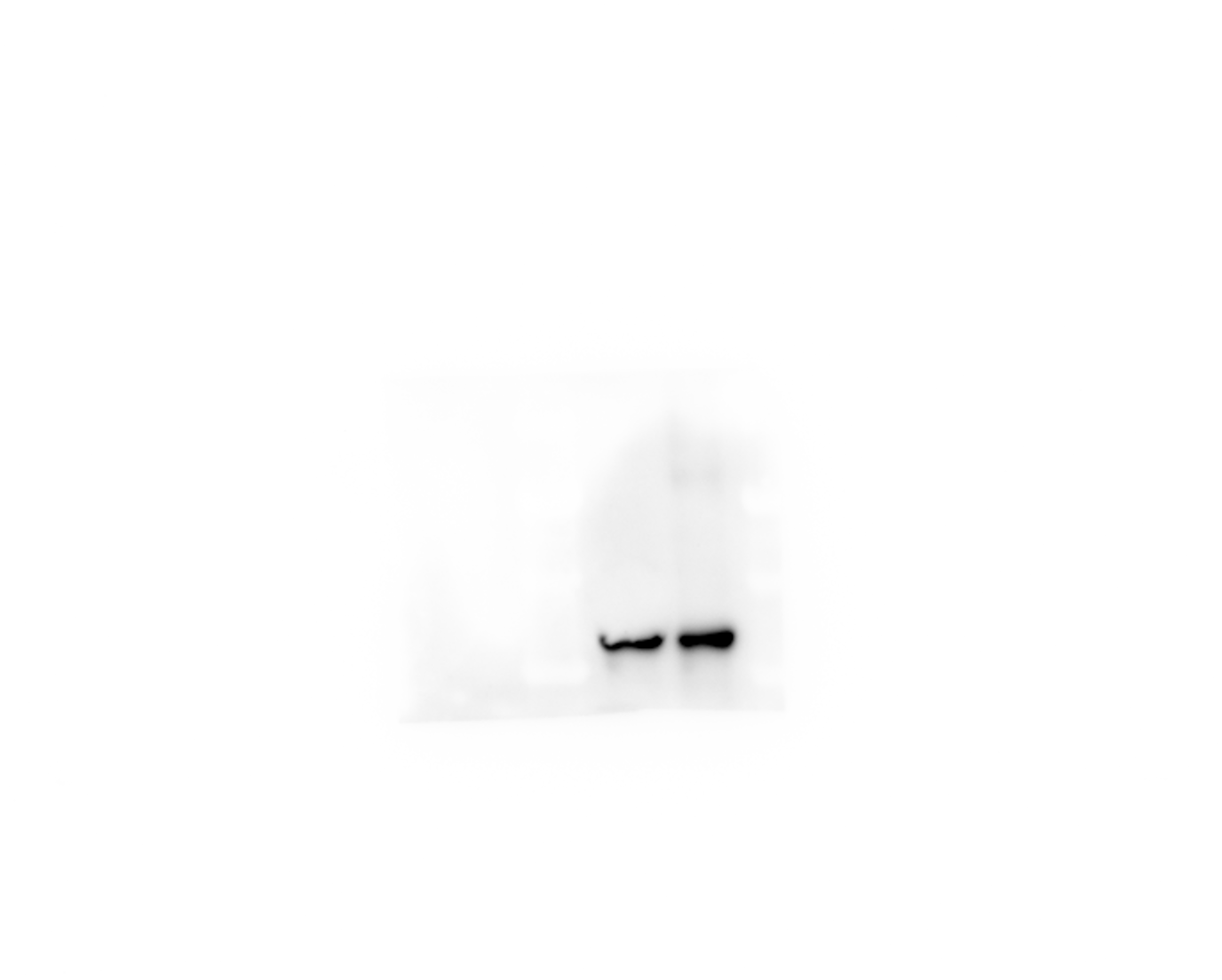

Supplement: Figure 3—source data 2. [file elife-103663-fig3-data2.zip › Figure 3/Figure 3J/Tublin-left.Tif]

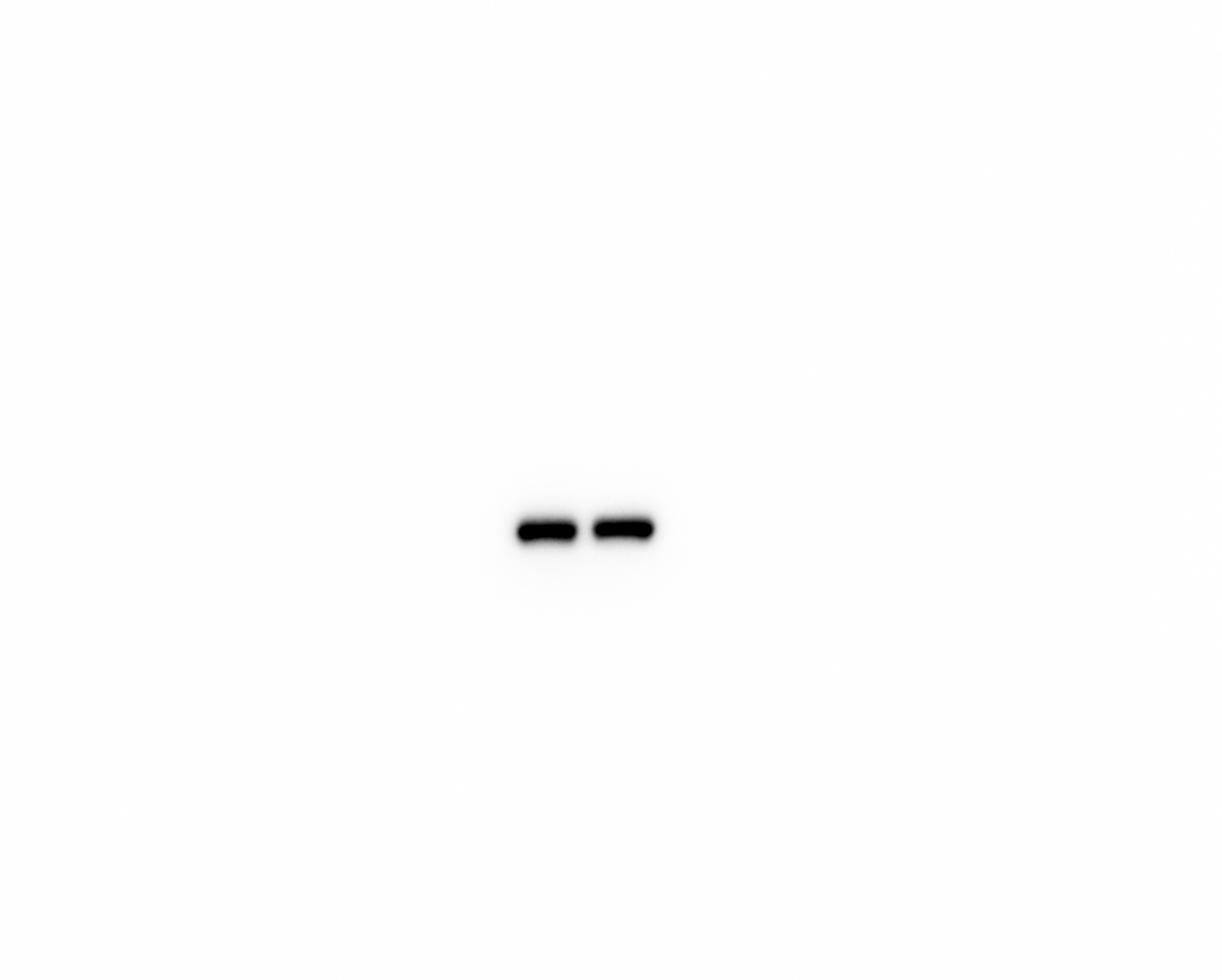

Supplement: Figure 3—source data 2. [file elife-103663-fig3-data2.zip › Figure 3/Figure 3J/Tublin-right.Tif]

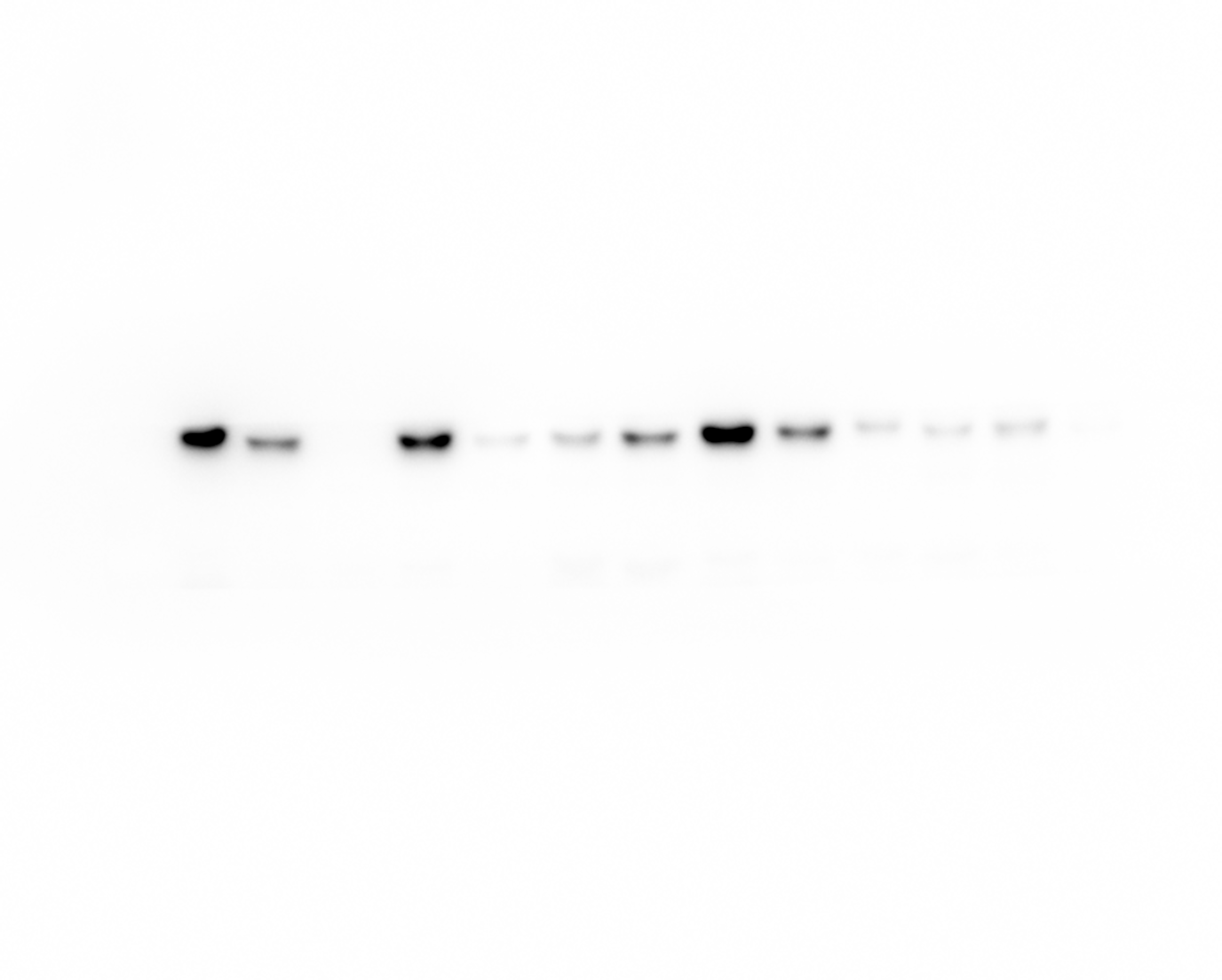

Supplement: Figure 3—source data 2. [file elife-103663-fig3-data2.zip › Figure 3/Figure 3L/FBP1-left.Tif]

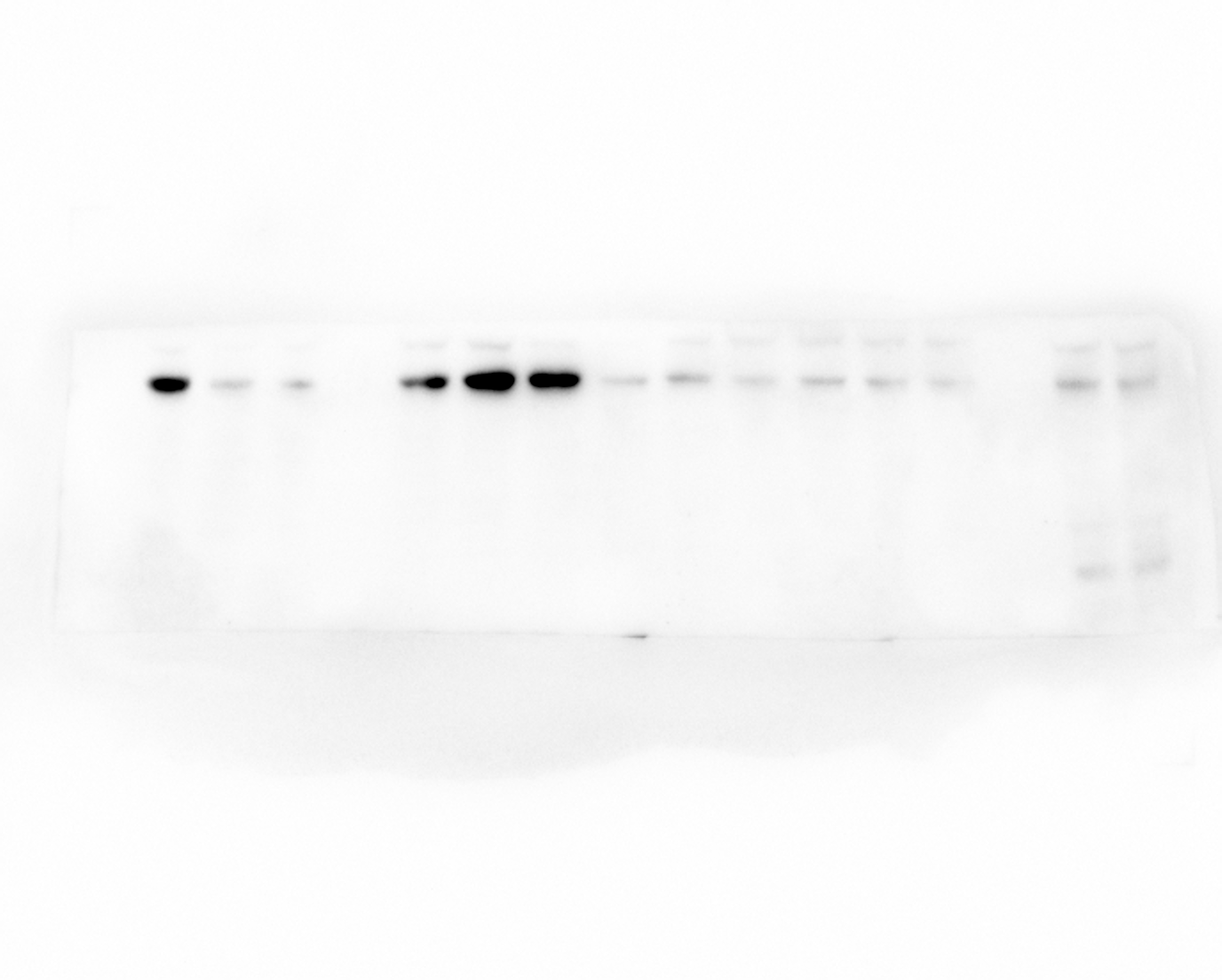

Supplement: Figure 3—source data 2. [file elife-103663-fig3-data2.zip › Figure 3/Figure 3L/FBP1-right.Tif]

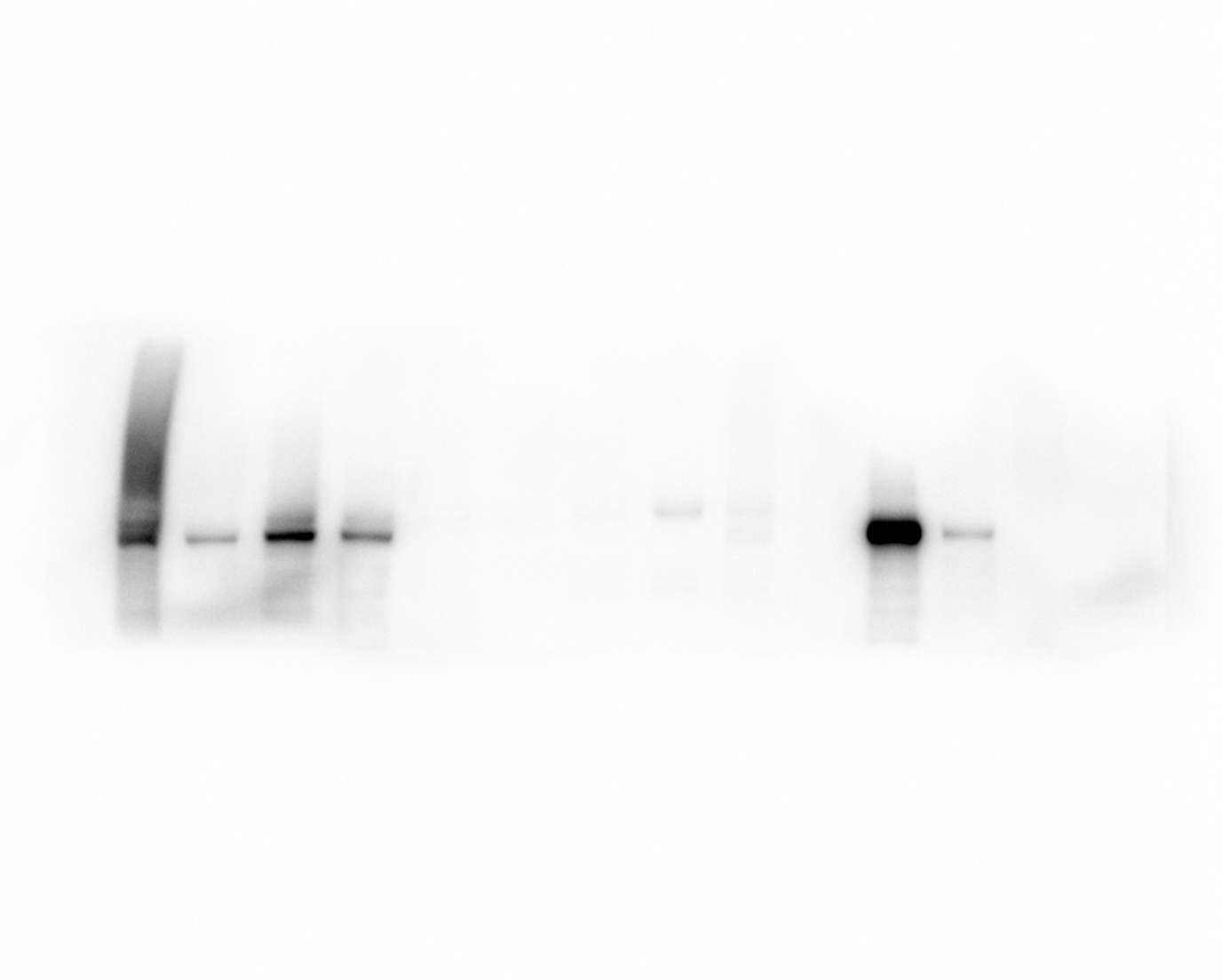

Supplement: Figure 3—source data 2. [file elife-103663-fig3-data2.zip › Figure 3/Figure 3L/TET2-left.Tif]

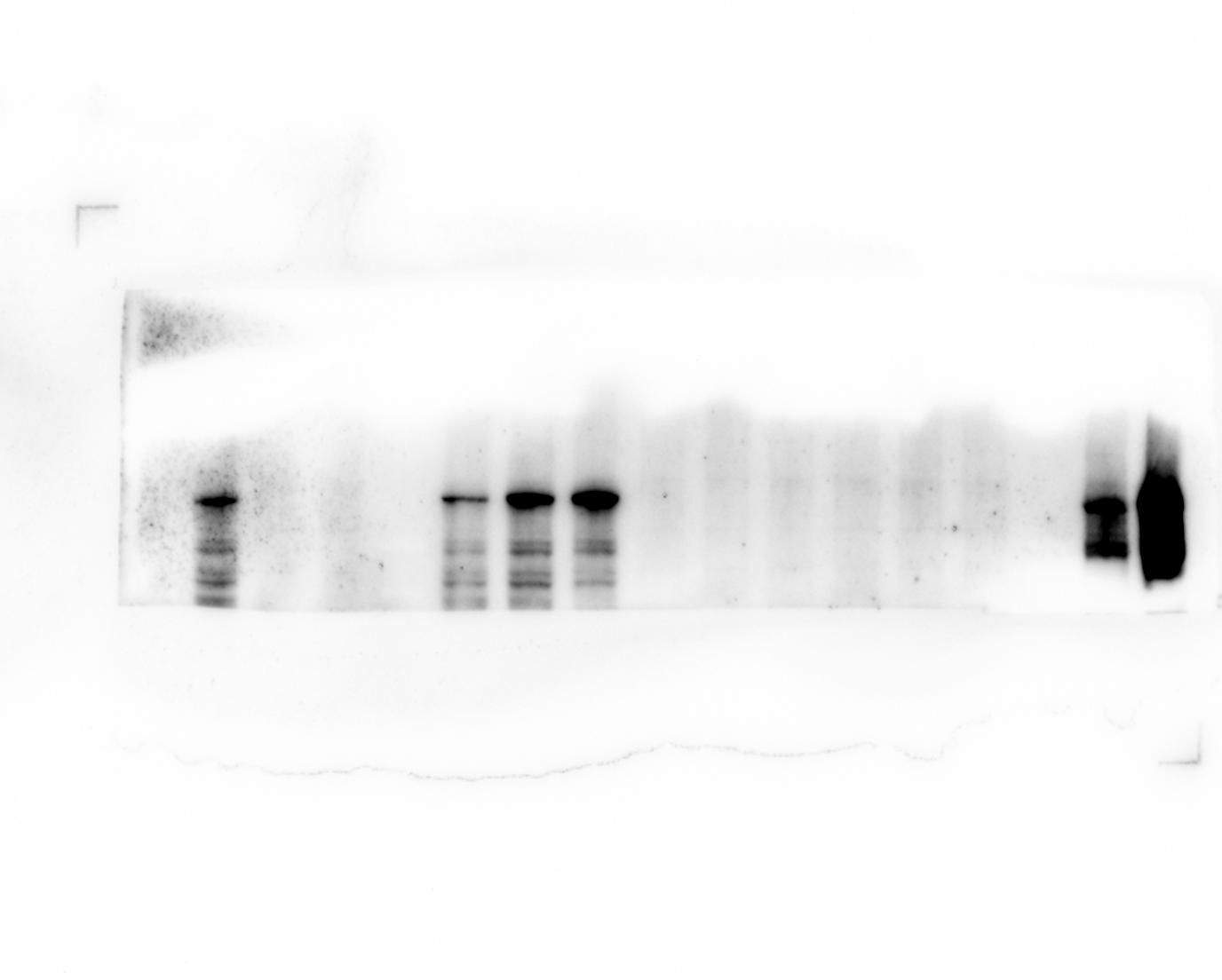

Supplement: Figure 3—source data 2. [file elife-103663-fig3-data2.zip › Figure 3/Figure 3L/TET2-right.Tif]

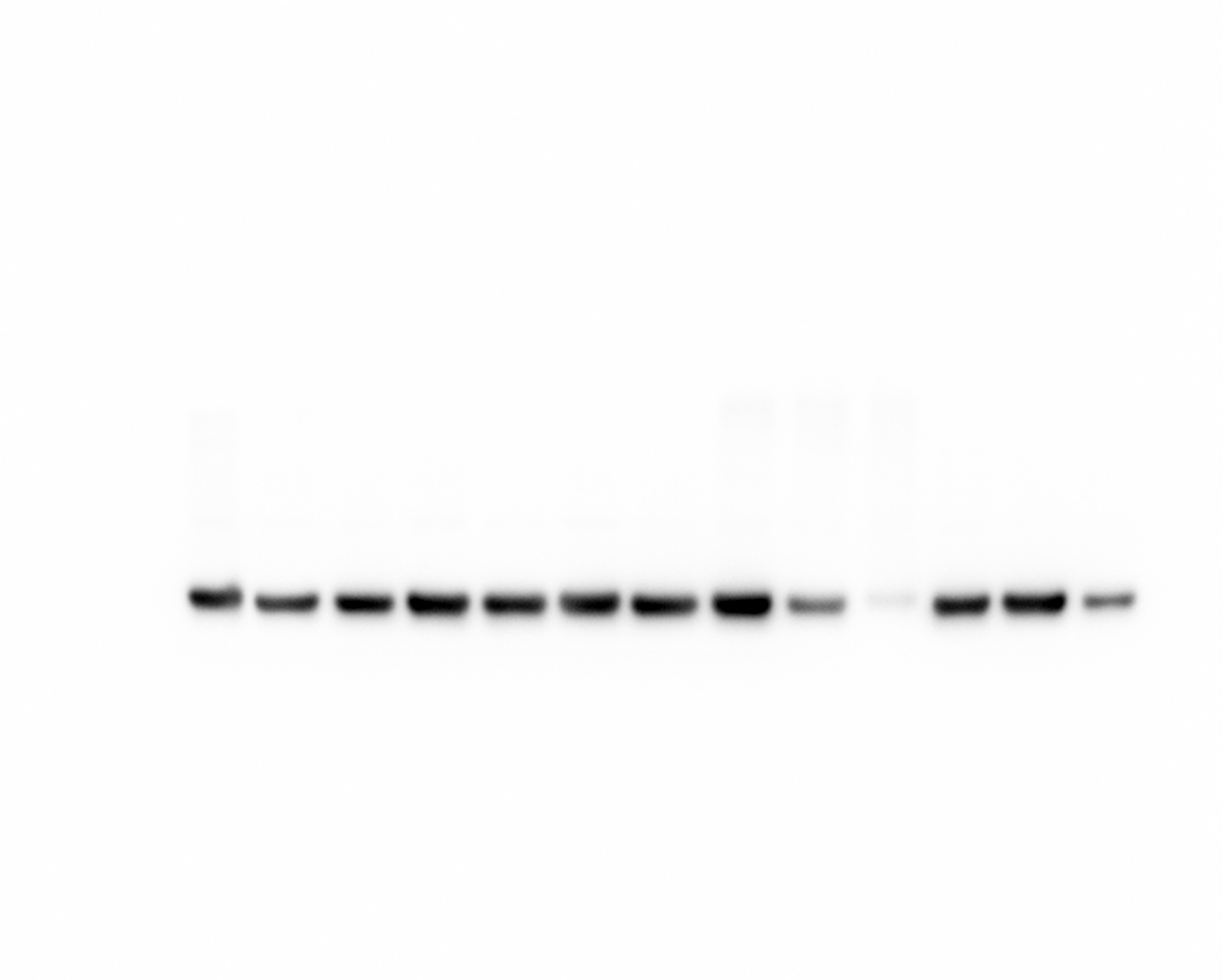

Supplement: Figure 3—source data 2. [file elife-103663-fig3-data2.zip › Figure 3/Figure 3L/Tublin-left.Tif]

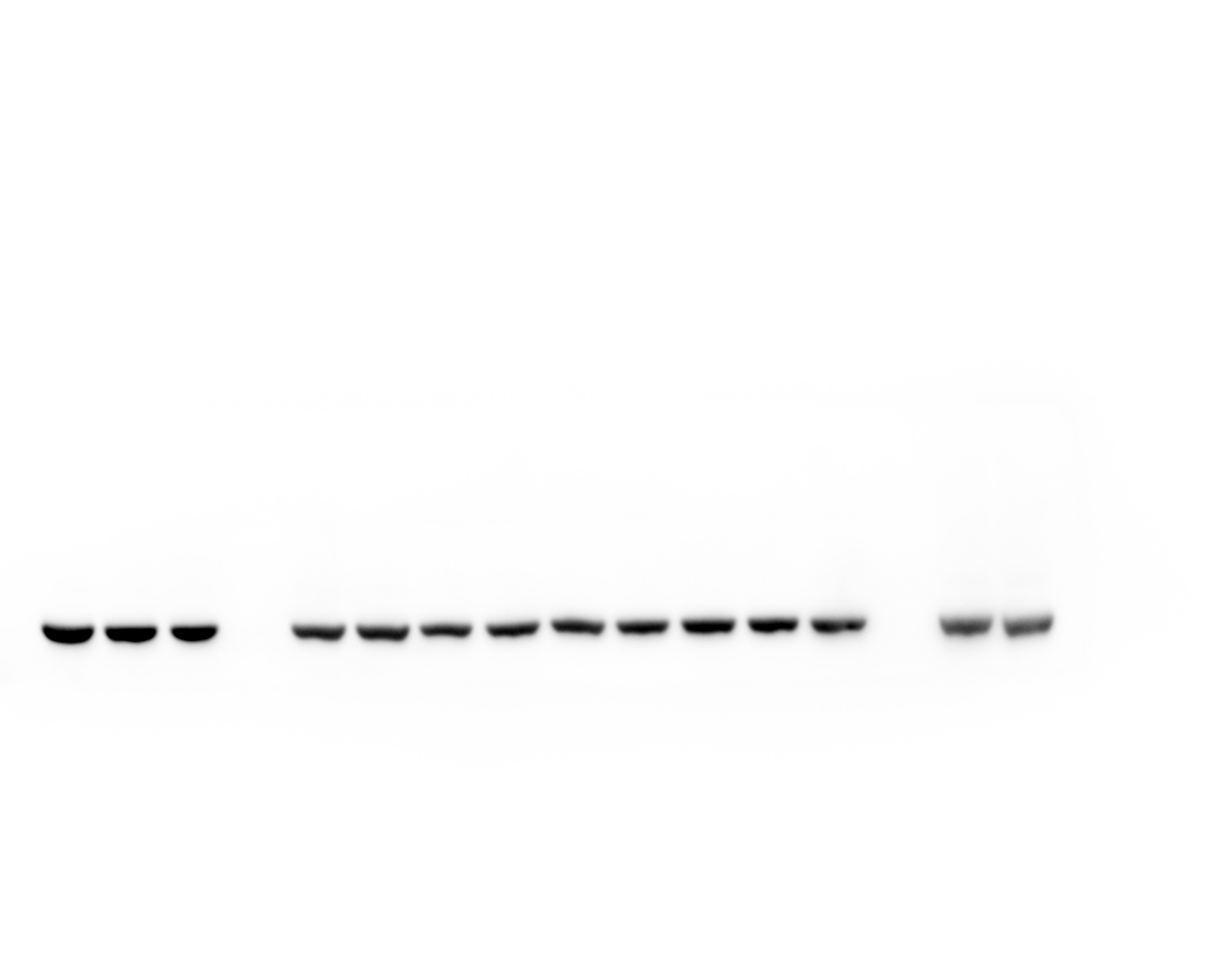

Supplement: Figure 3—source data 2. [file elife-103663-fig3-data2.zip › Figure 3/Figure 3L/Tublin-right.Tif]

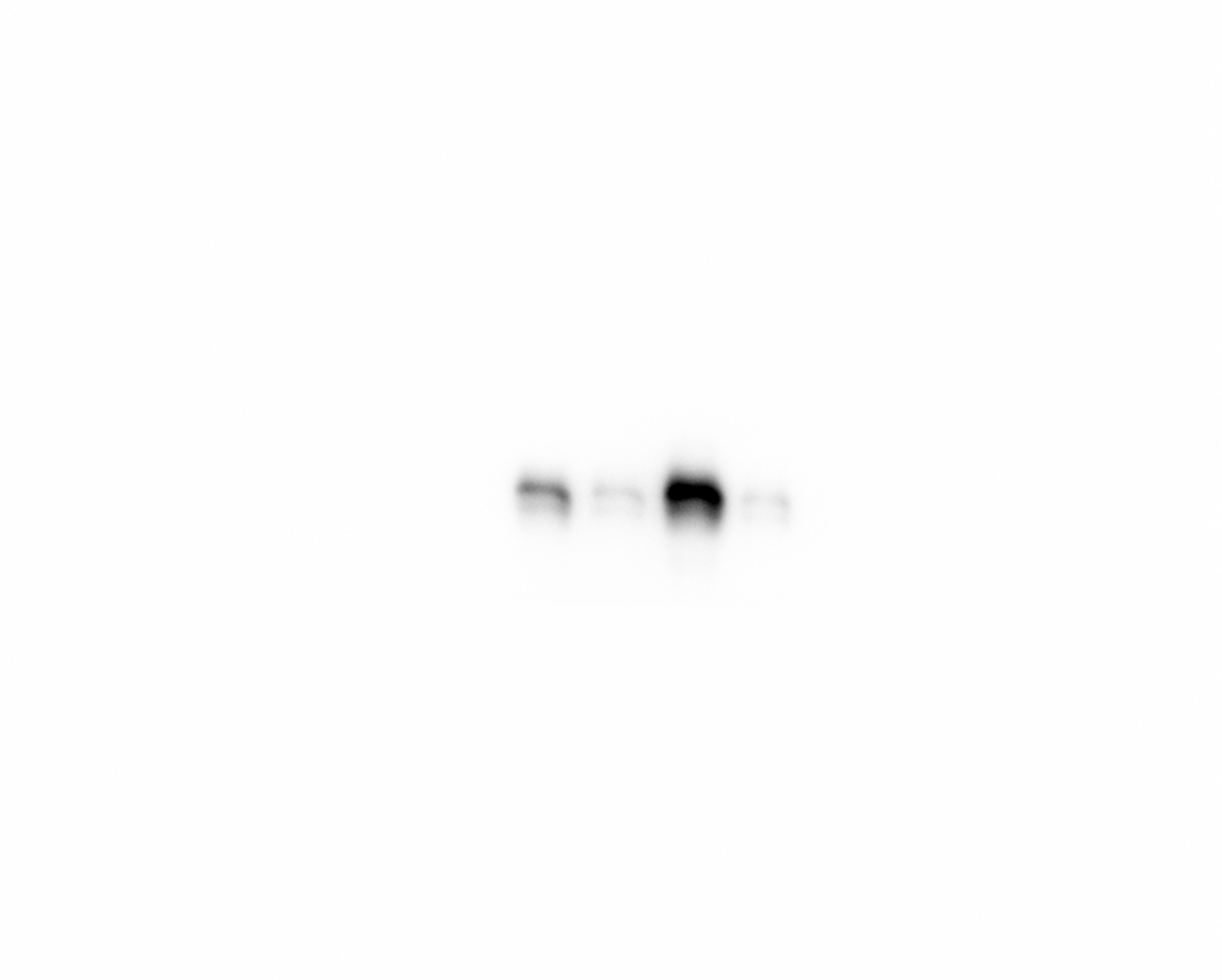

Supplement: Figure 3—source data 2. [file elife-103663-fig3-data2.zip › Figure 3/Figure 3M/Fbp1-left.Tif]

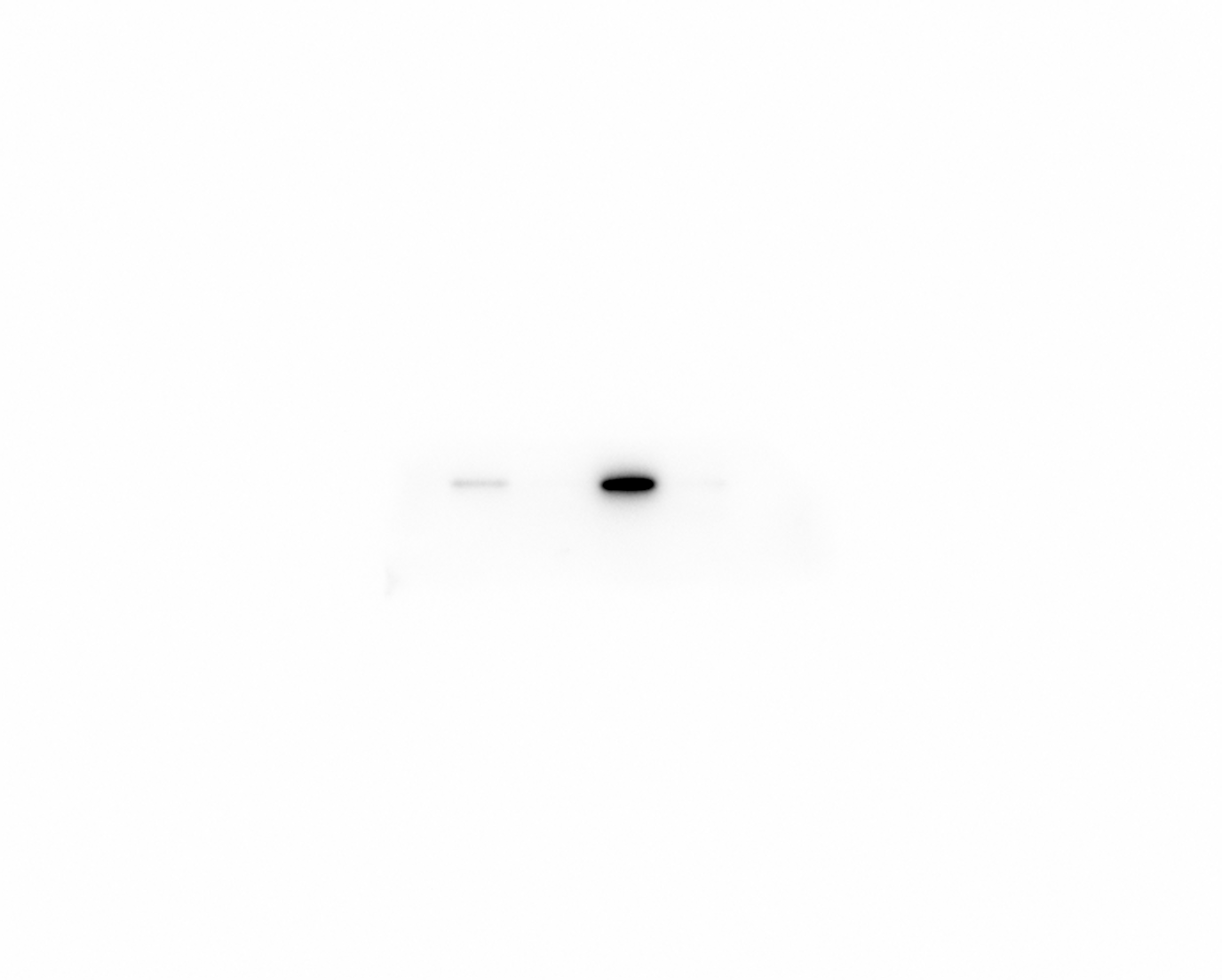

Supplement: Figure 3—source data 2. [file elife-103663-fig3-data2.zip › Figure 3/Figure 3M/Fbp1-right.Tif]

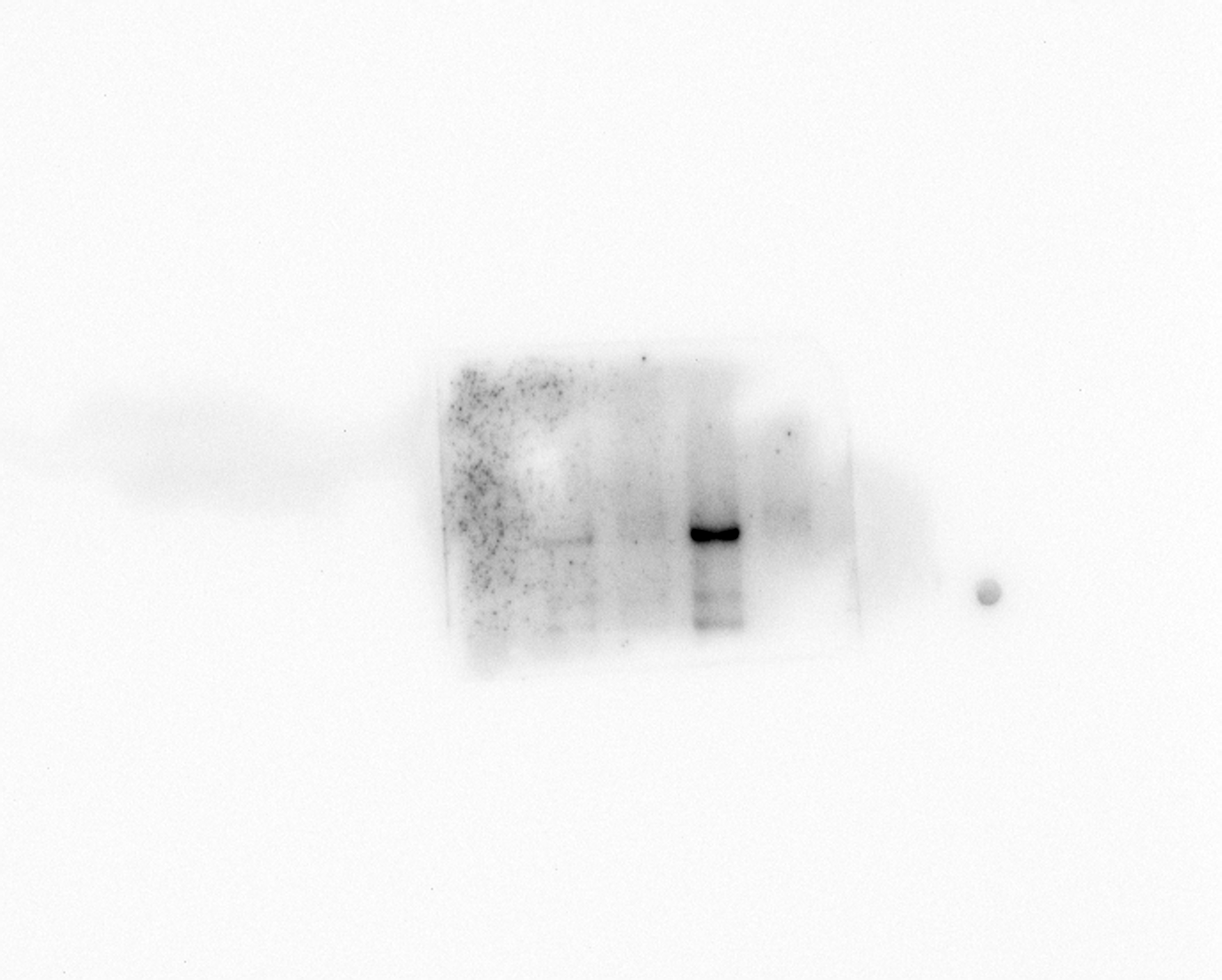

Supplement: Figure 3—source data 2. [file elife-103663-fig3-data2.zip › Figure 3/Figure 3M/Tet2-left.Tif]

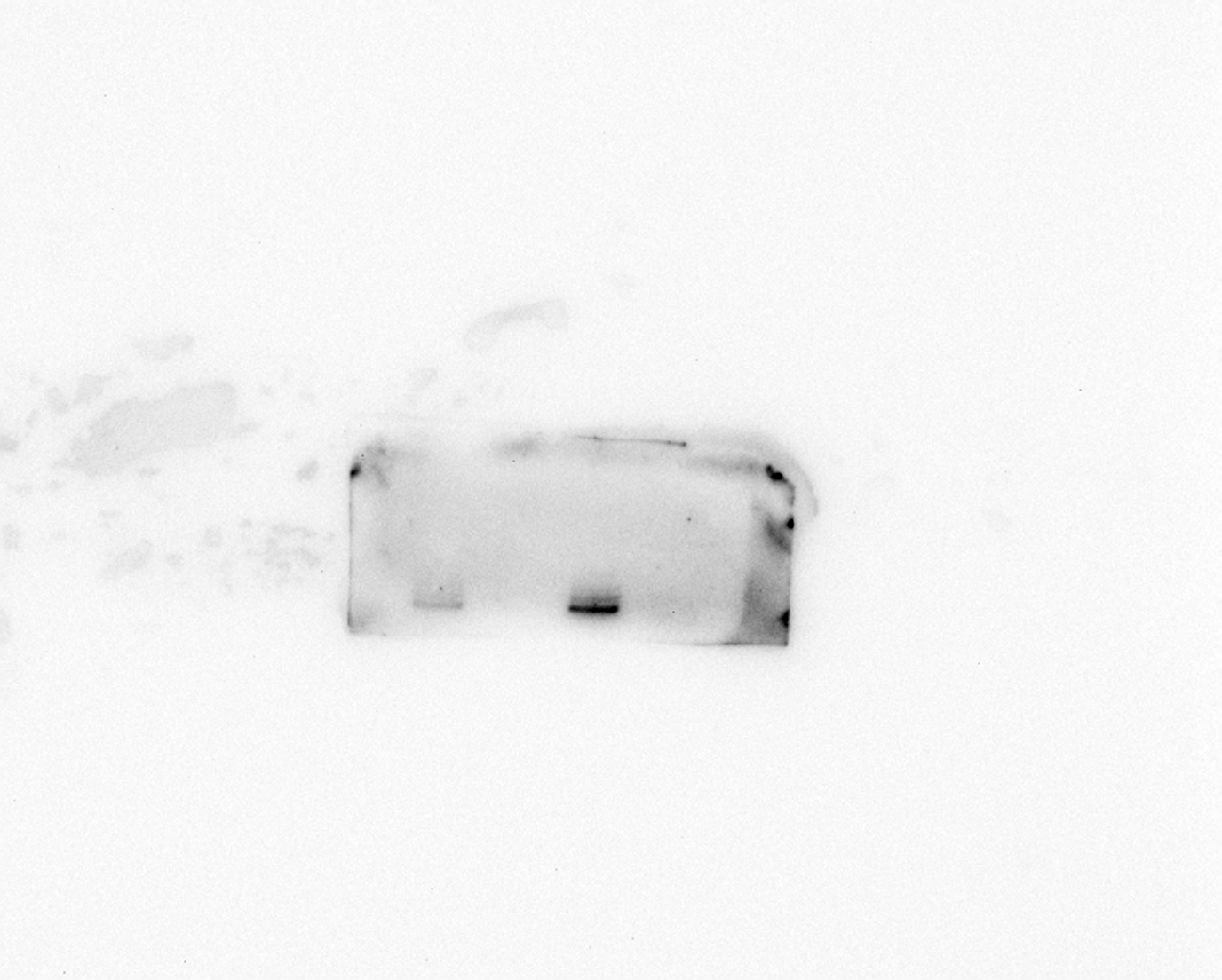

Supplement: Figure 3—source data 2. [file elife-103663-fig3-data2.zip › Figure 3/Figure 3M/Tet2-right.Tif]

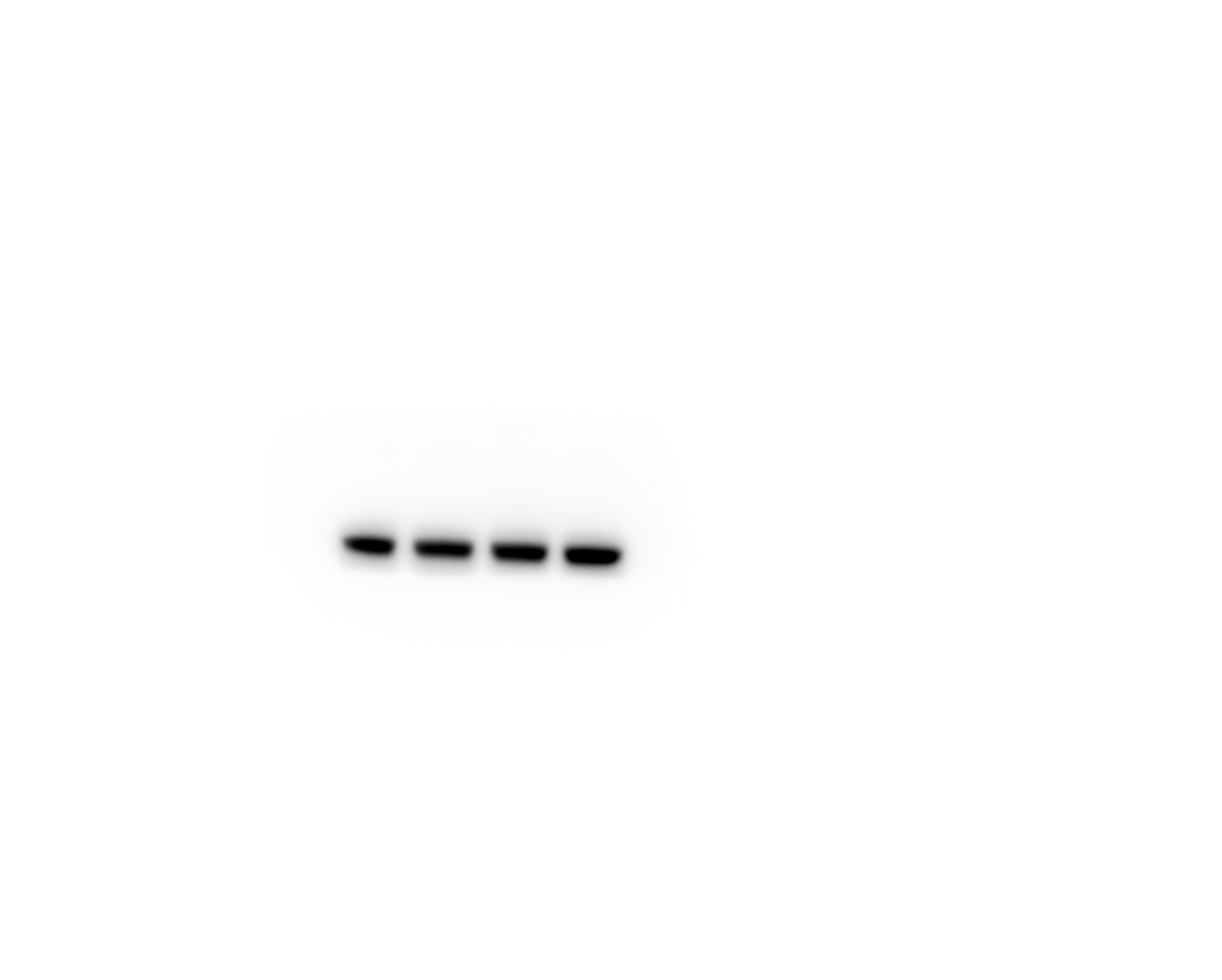

Supplement: Figure 3—source data 2. [file elife-103663-fig3-data2.zip › Figure 3/Figure 3M/Tubulin-left.Tif]

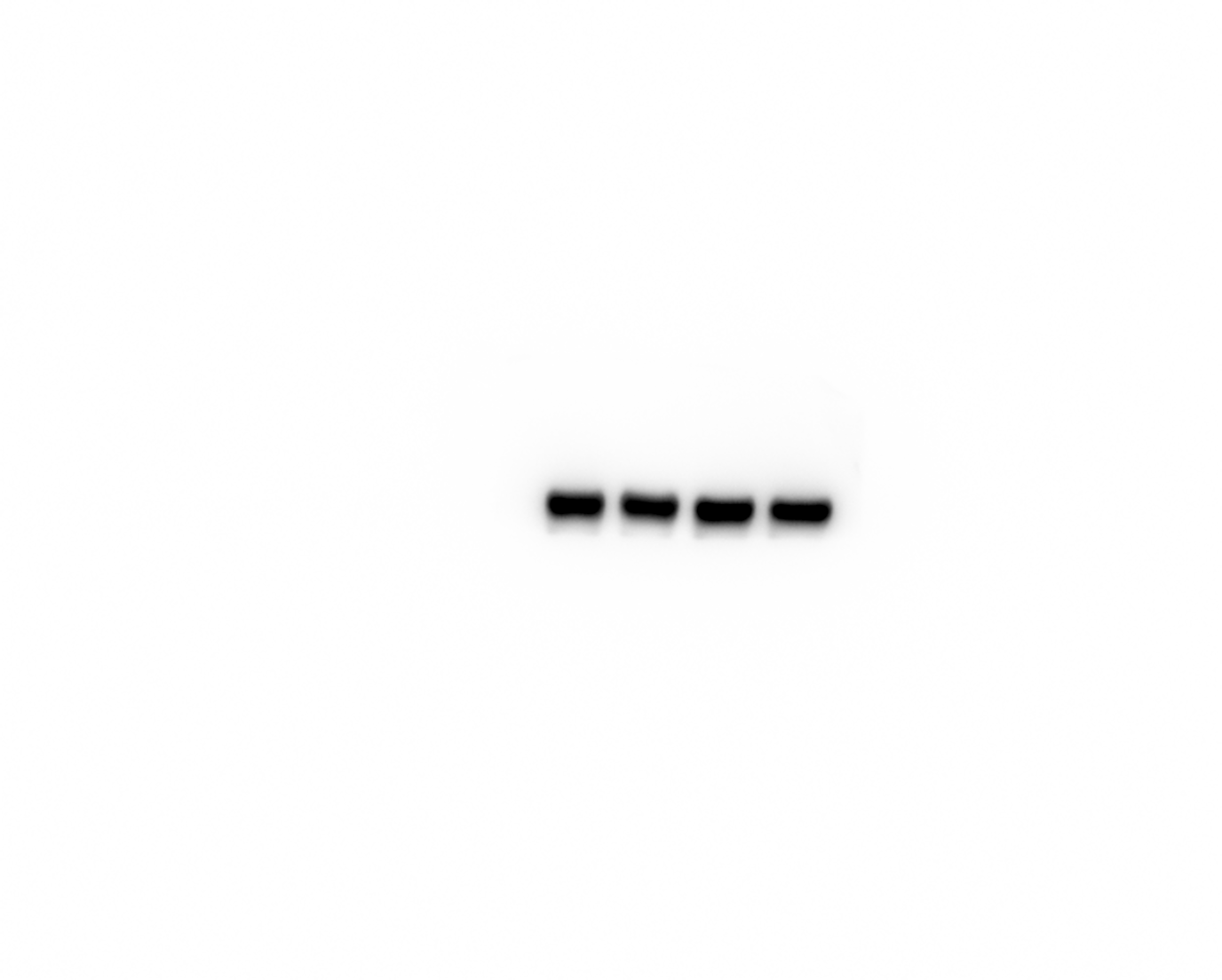

Supplement: Figure 3—source data 2. [file elife-103663-fig3-data2.zip › Figure 3/Figure 3M/Tubulin-right.Tif]

Figure. 4D

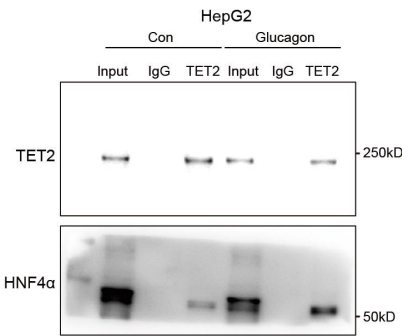

Figure. 4E

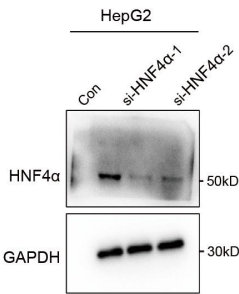

Figure. 4I

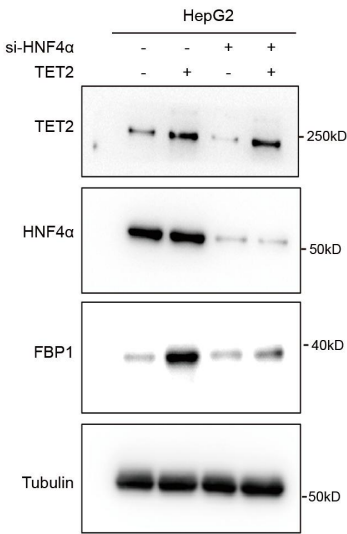

Figure. 4K

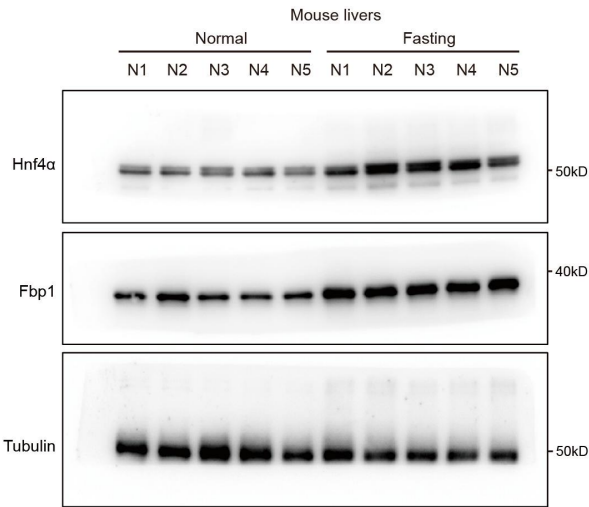

Figure. 4L

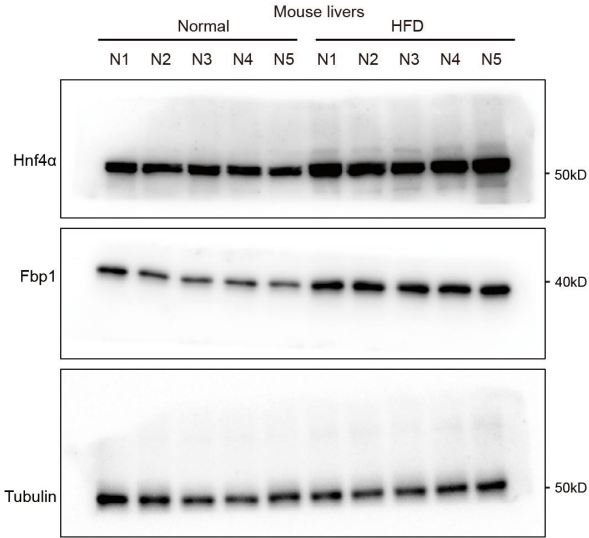

Figure. 4M

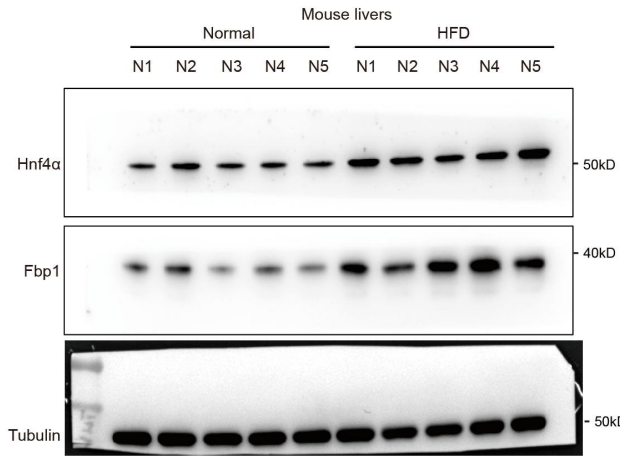

Supplement: Figure 4—source data 1. [file elife-103663-fig4-data1.pdf]

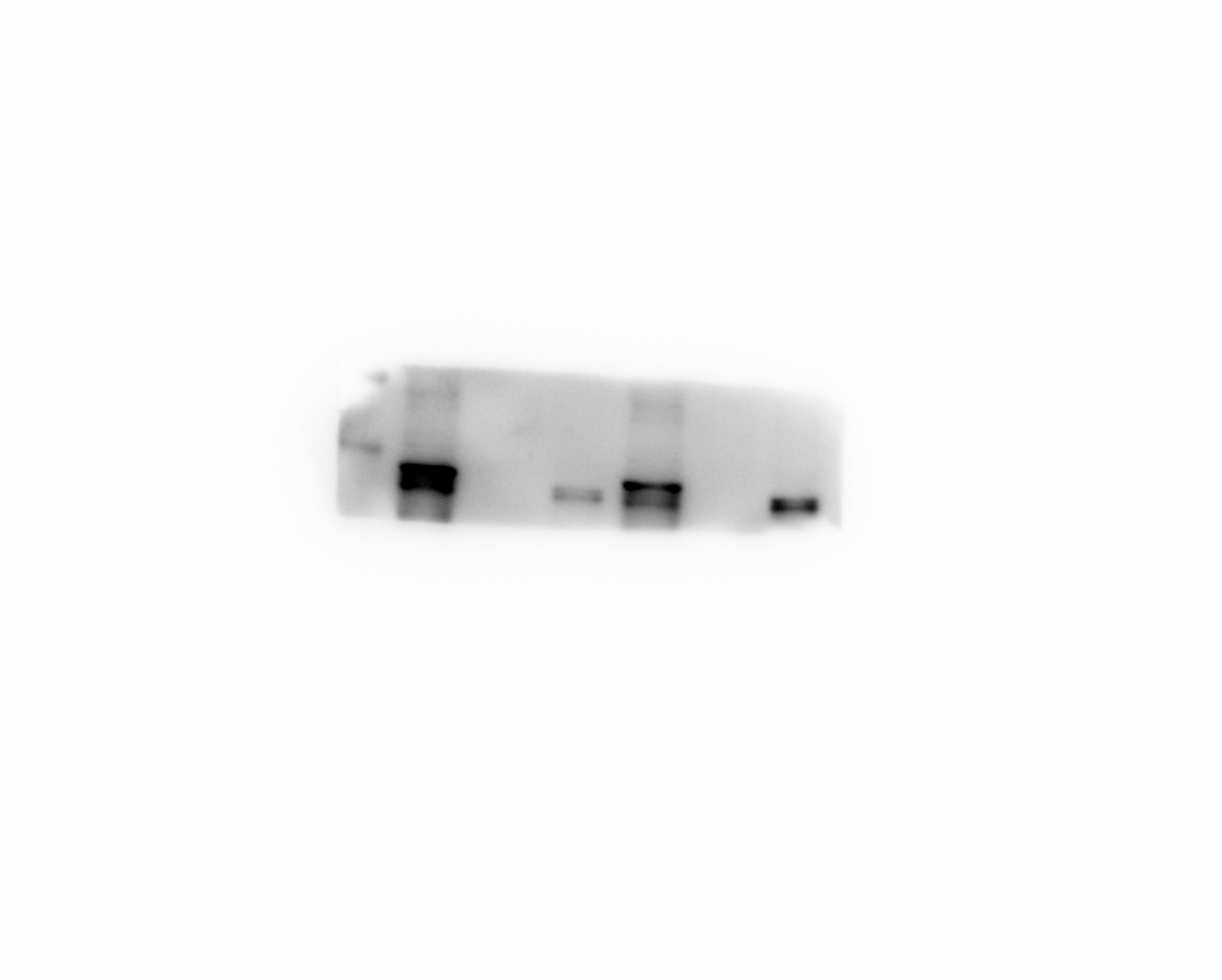

Supplement: Figure 4—source data 2. [file elife-103663-fig4-data2.zip › Figure 4/Figure 4D/HNF4α.Tif]

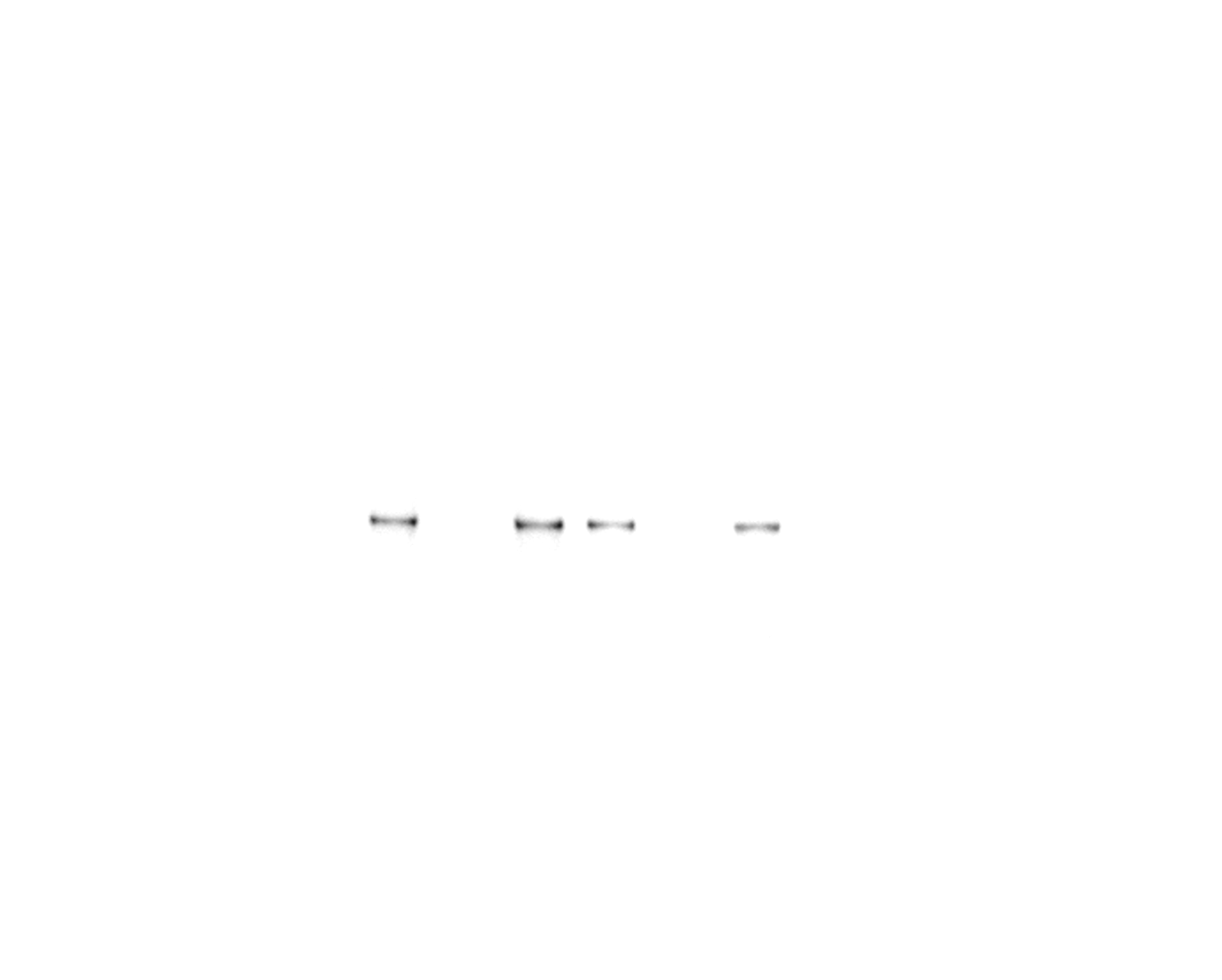

Supplement: Figure 4—source data 2. [file elife-103663-fig4-data2.zip › Figure 4/Figure 4D/TET2.Tif]

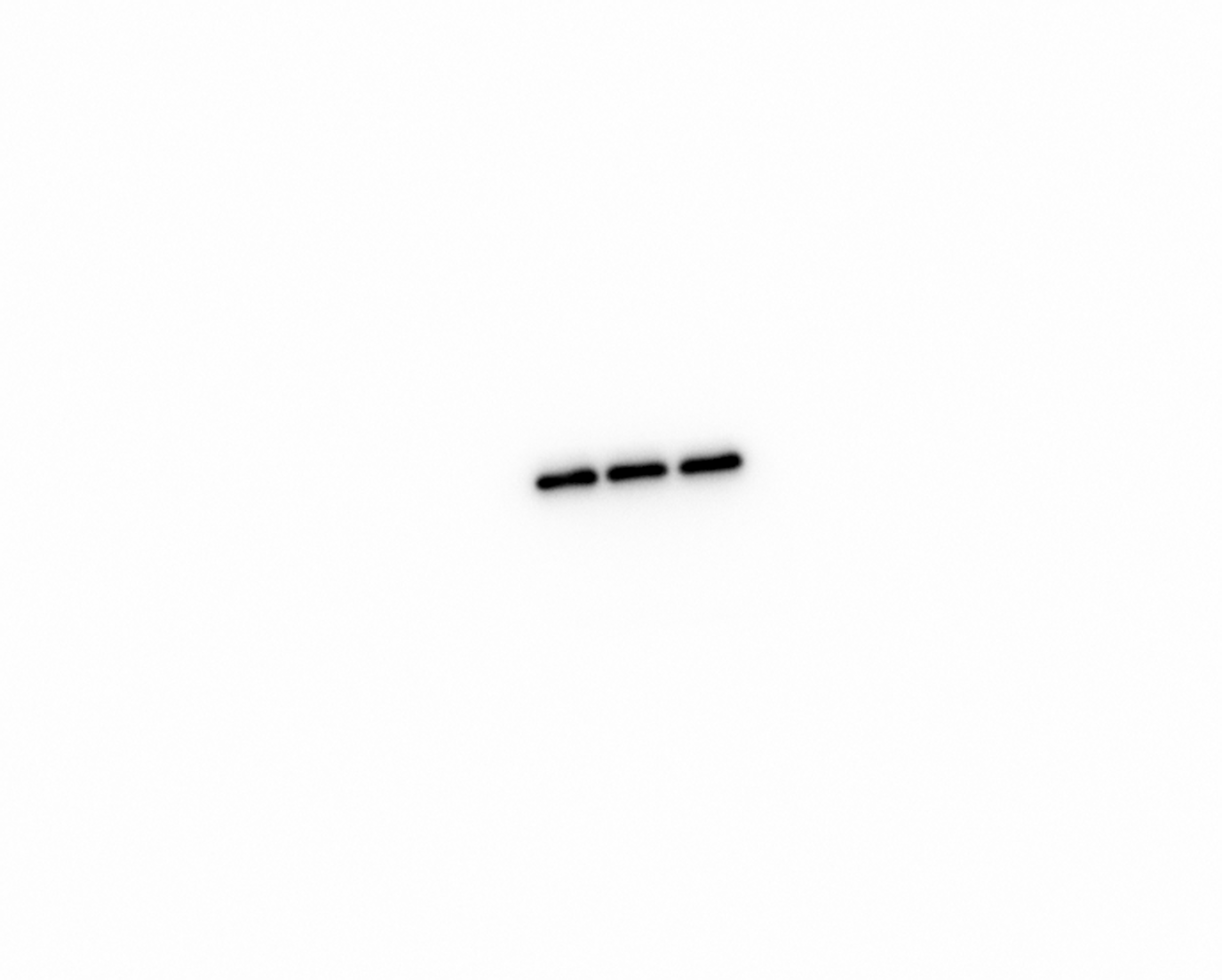

Supplement: Figure 4—source data 2. [file elife-103663-fig4-data2.zip › Figure 4/Figure 4E/GAPDH.Tif]

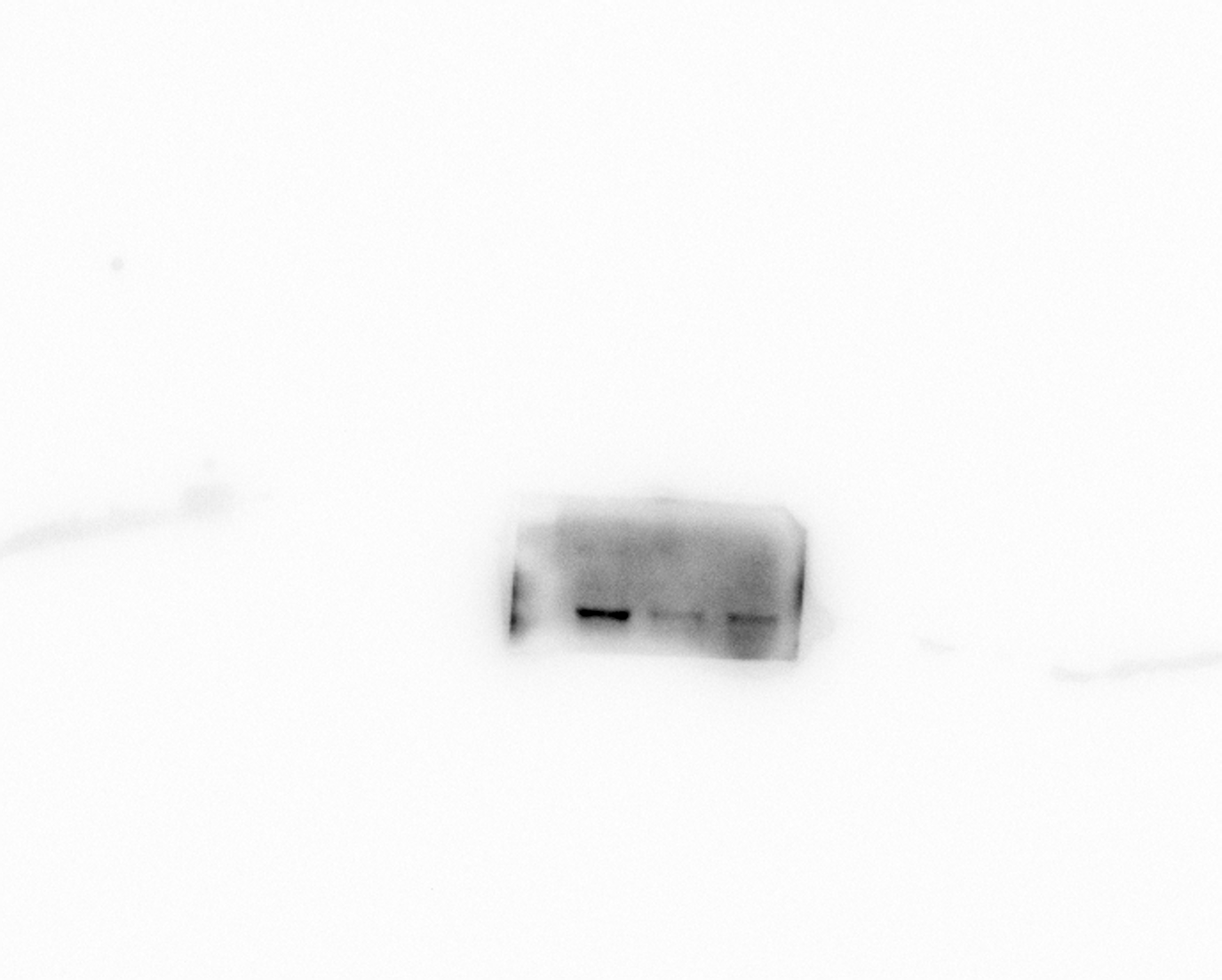

Supplement: Figure 4—source data 2. [file elife-103663-fig4-data2.zip › Figure 4/Figure 4E/HNF4α.Tif]

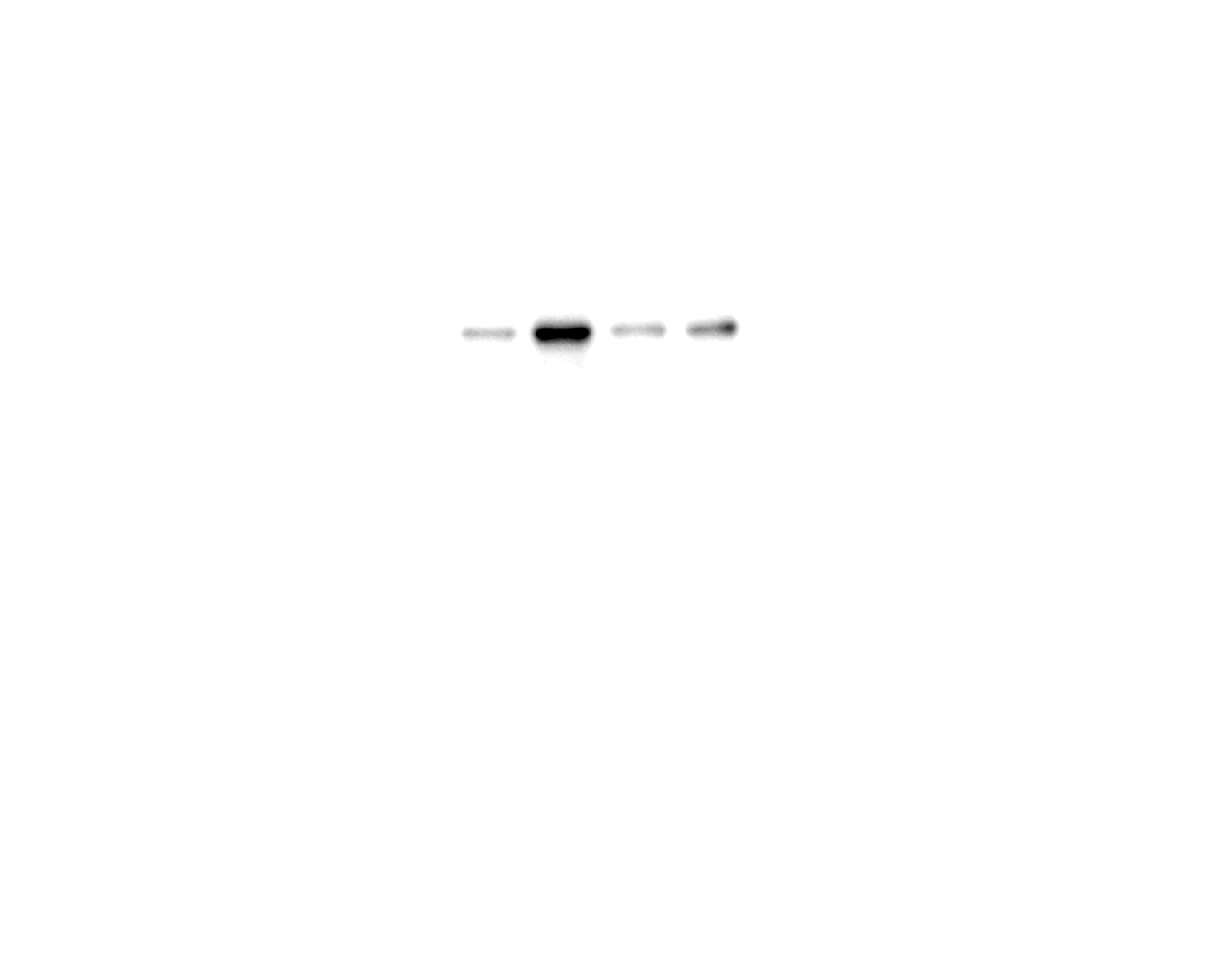

Supplement: Figure 4—source data 2. [file elife-103663-fig4-data2.zip › Figure 4/Figure 4I/FBP1.Tif]

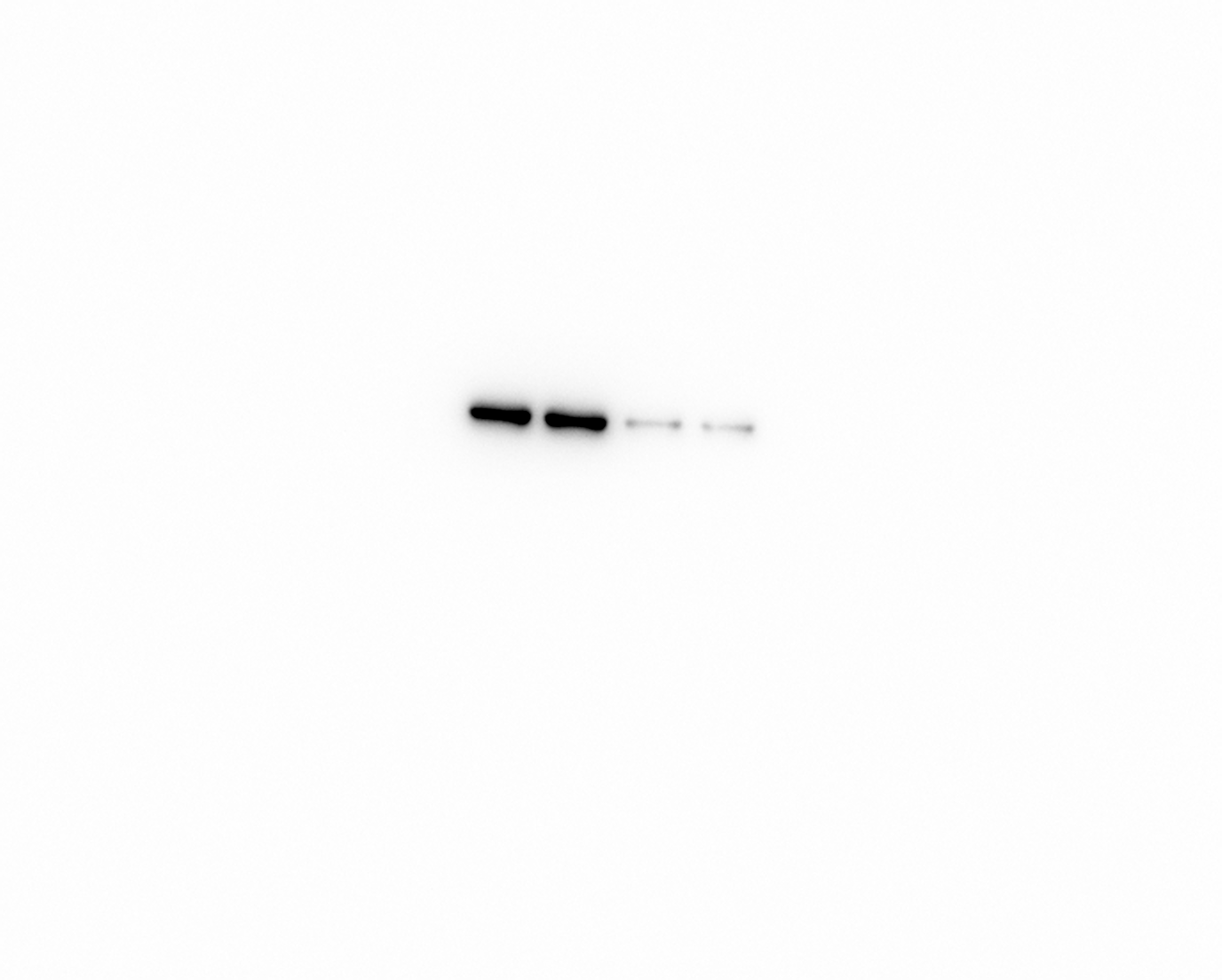

Supplement: Figure 4—source data 2. [file elife-103663-fig4-data2.zip › Figure 4/Figure 4I/HNF4α.Tif]

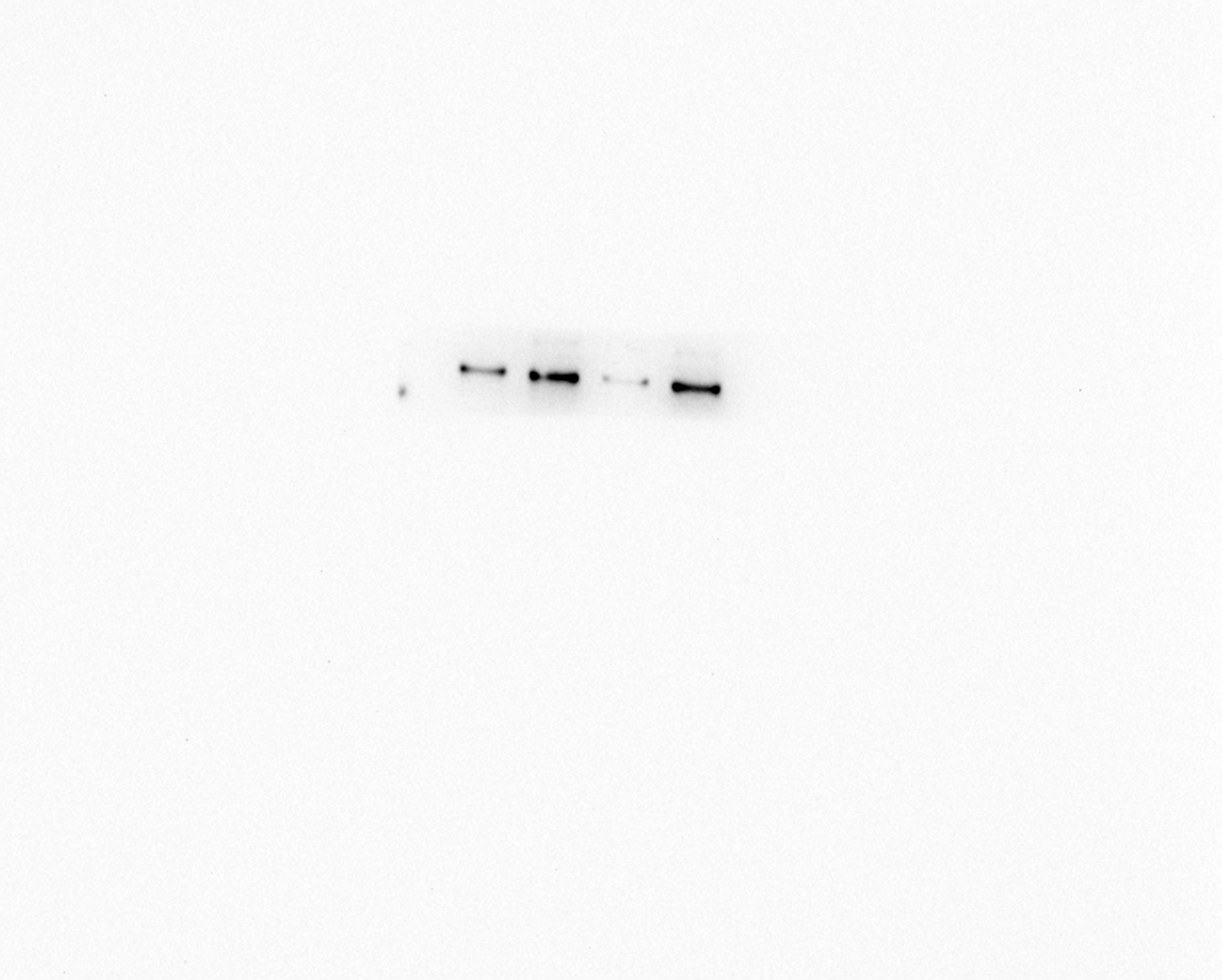

Supplement: Figure 4—source data 2. [file elife-103663-fig4-data2.zip › Figure 4/Figure 4I/TET2.Tif]

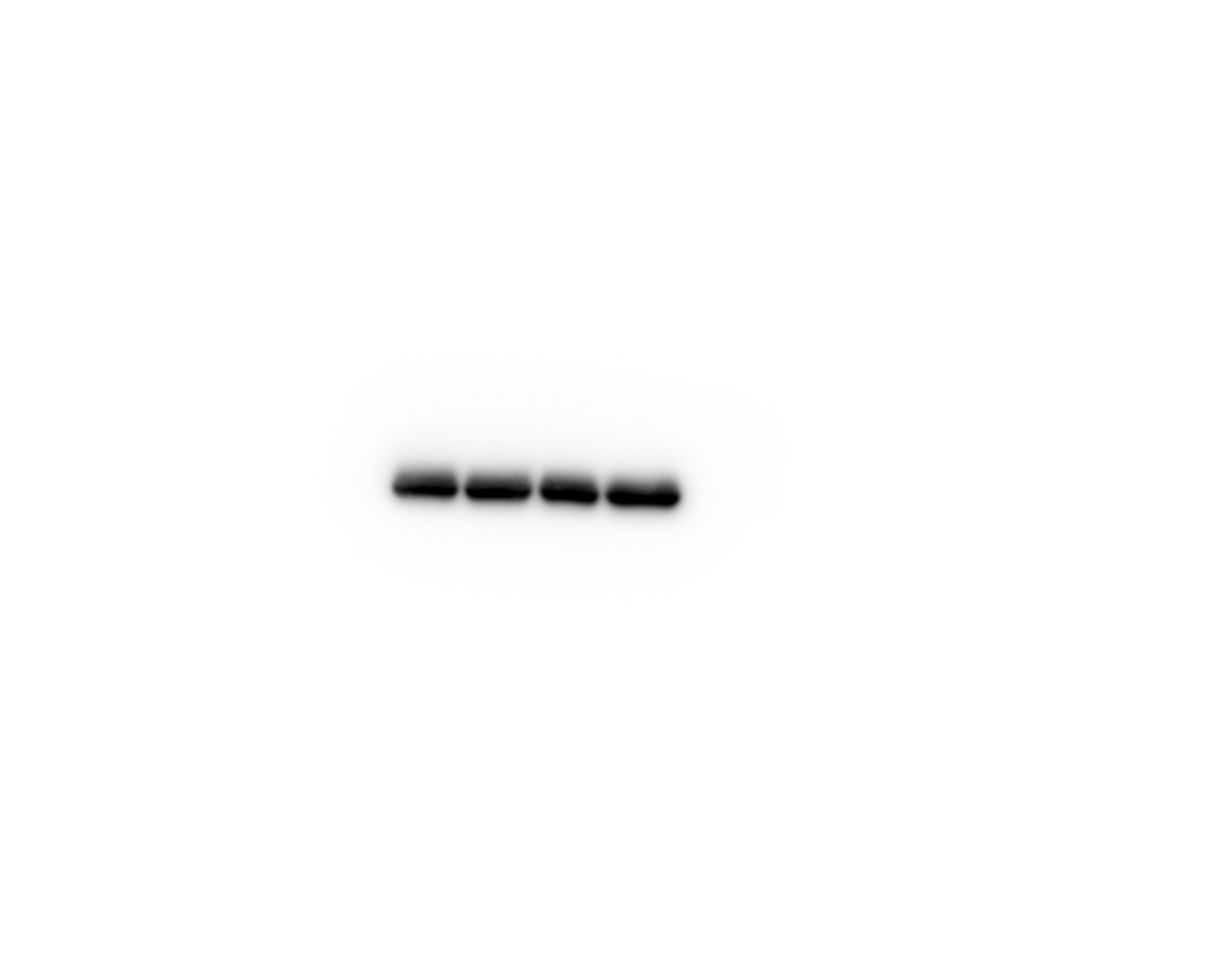

Supplement: Figure 4—source data 2. [file elife-103663-fig4-data2.zip › Figure 4/Figure 4I/Tubulin.Tif]

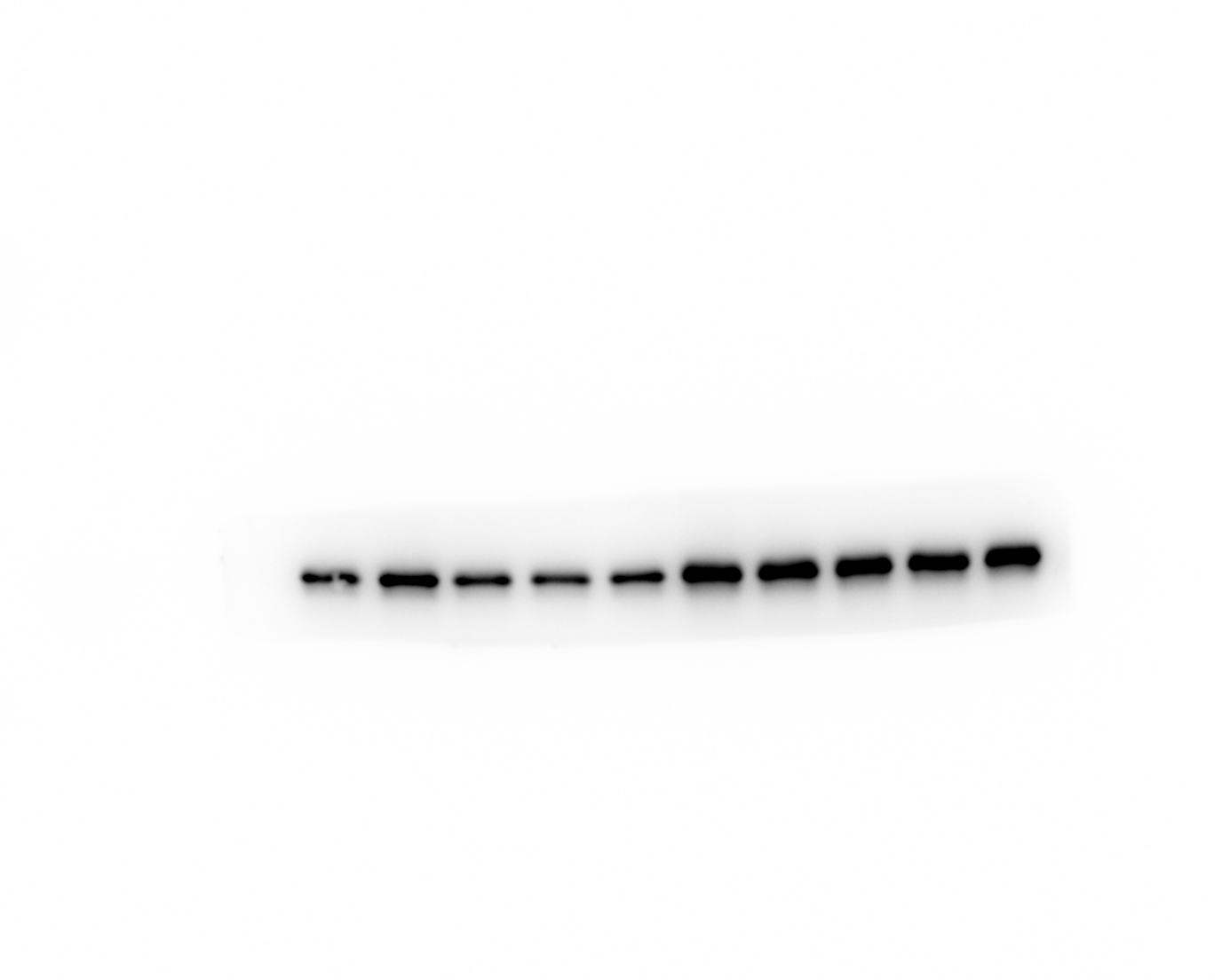

Supplement: Figure 4—source data 2. [file elife-103663-fig4-data2.zip › Figure 4/Figure 4K/Fbp1.Tif]

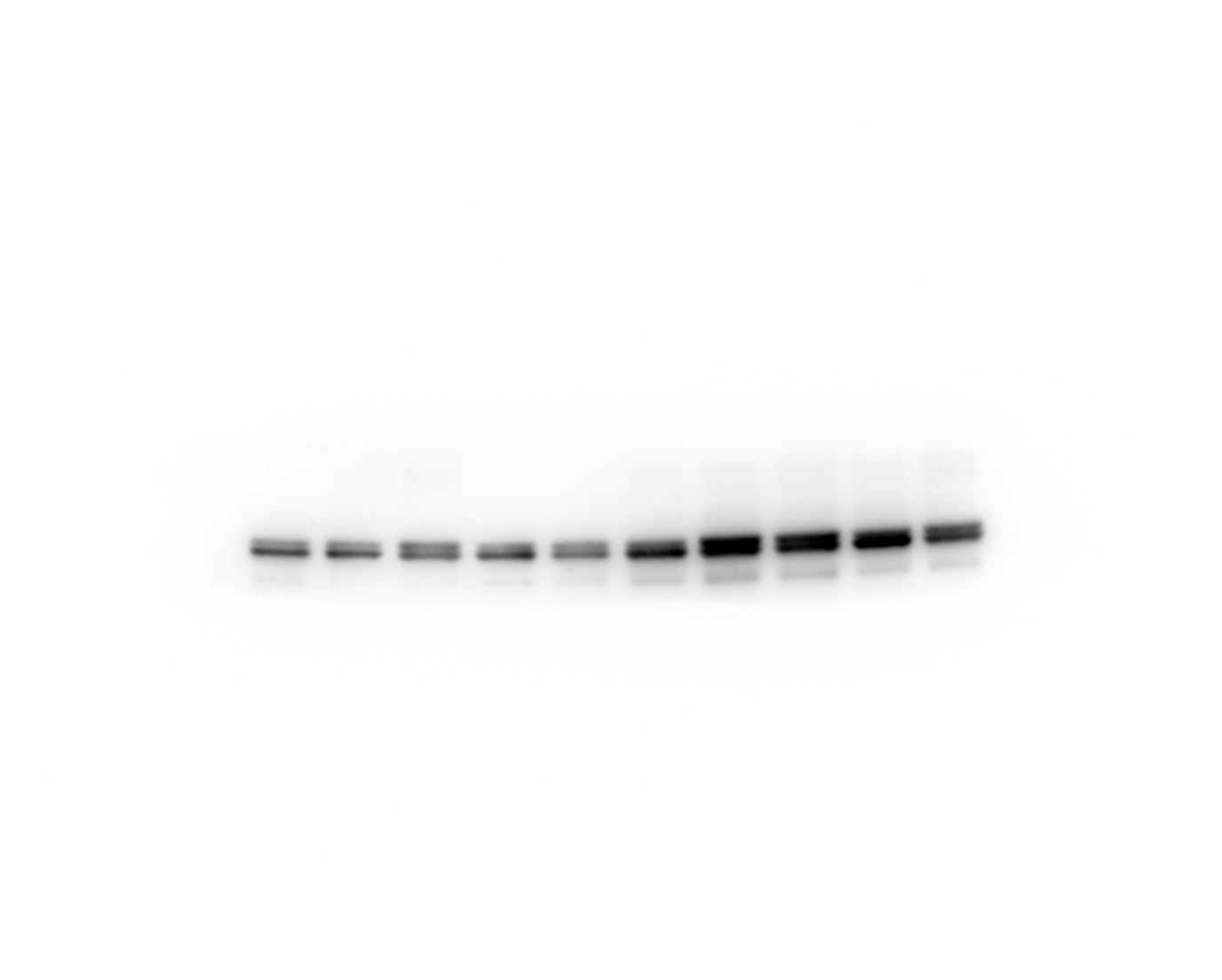

Supplement: Figure 4—source data 2. [file elife-103663-fig4-data2.zip › Figure 4/Figure 4K/Hnf4α.Tif]

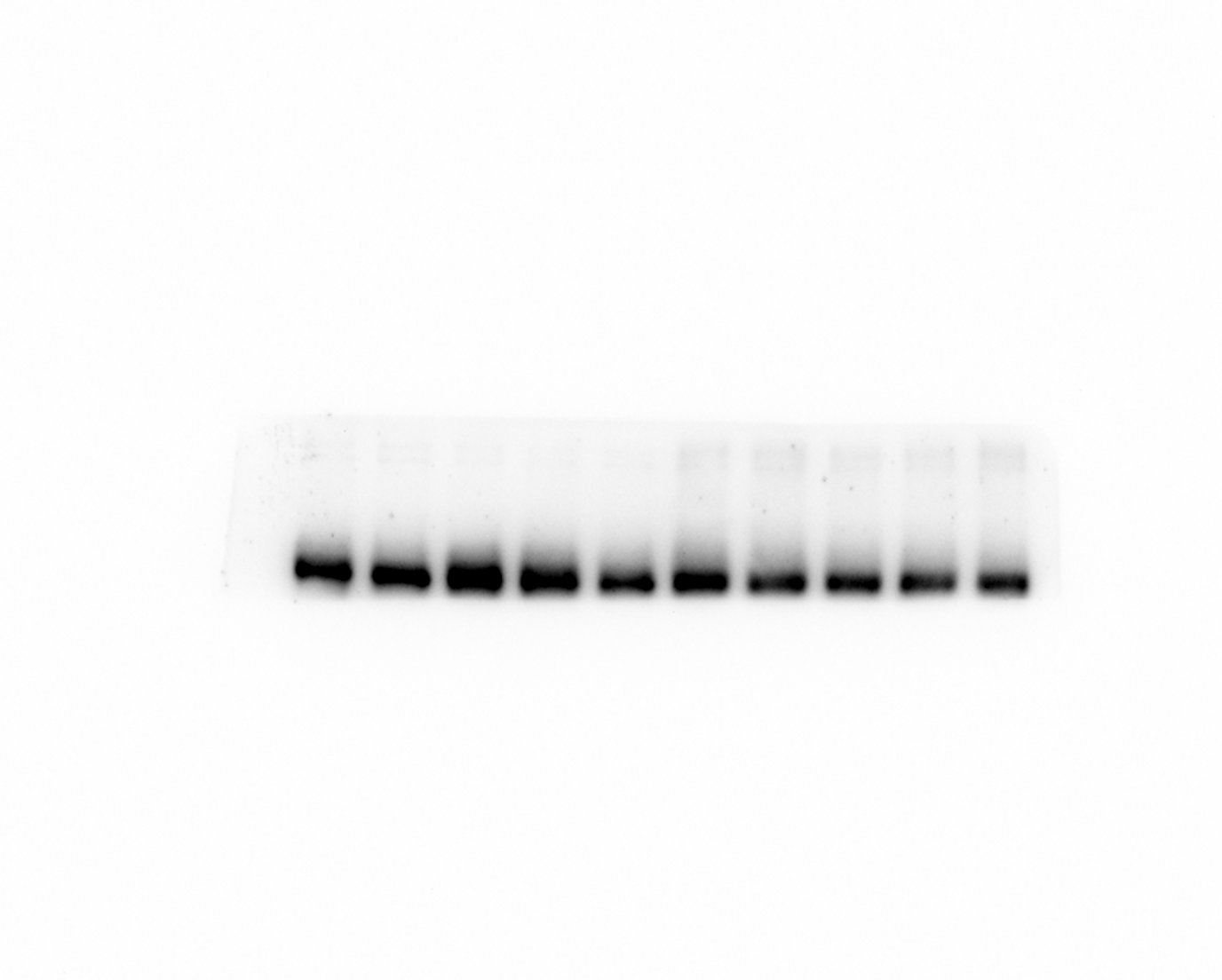

Supplement: Figure 4—source data 2. [file elife-103663-fig4-data2.zip › Figure 4/Figure 4K/Tubulin.Tif]

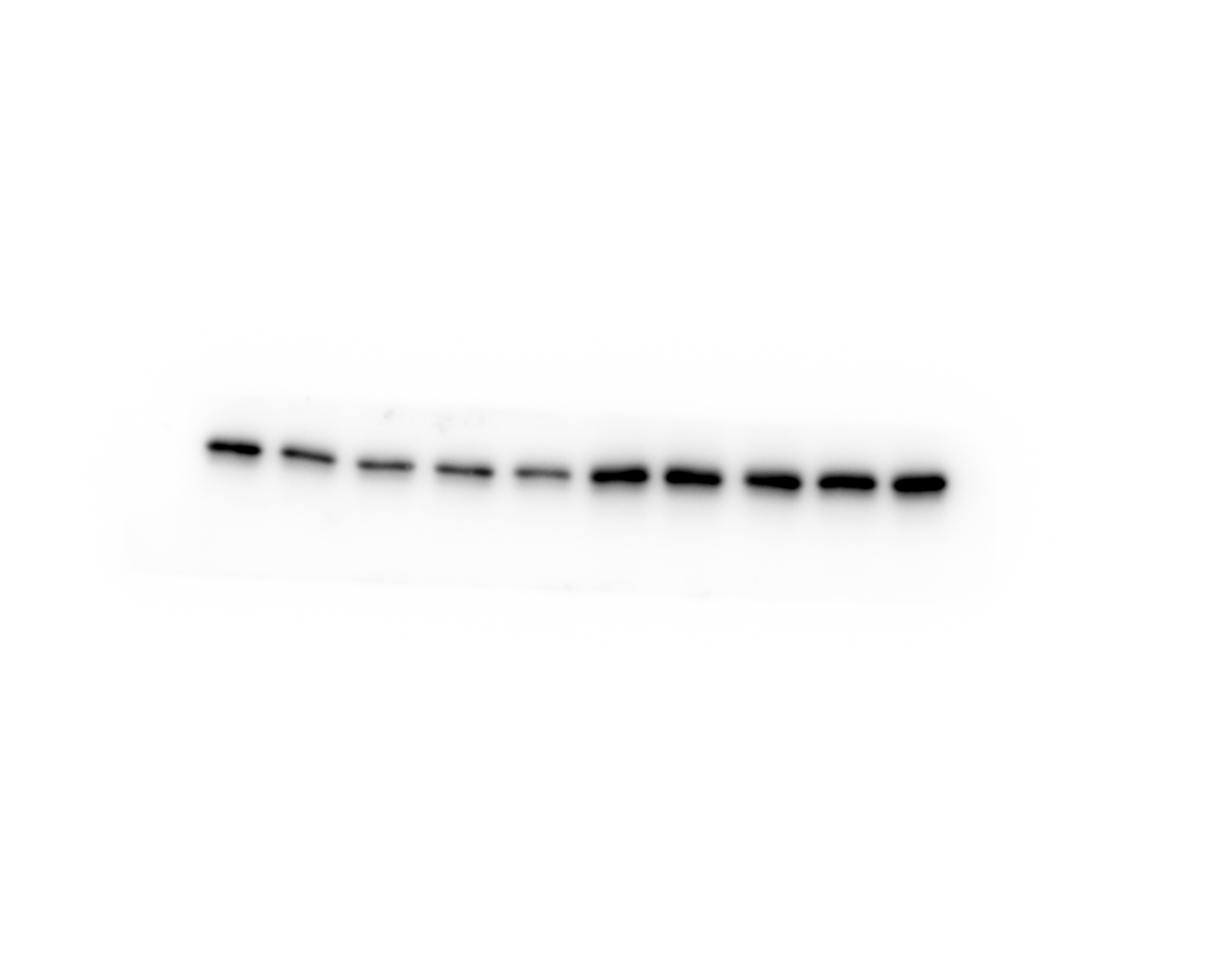

Supplement: Figure 4—source data 2. [file elife-103663-fig4-data2.zip › Figure 4/Figure 4L/Fbp1.Tif]

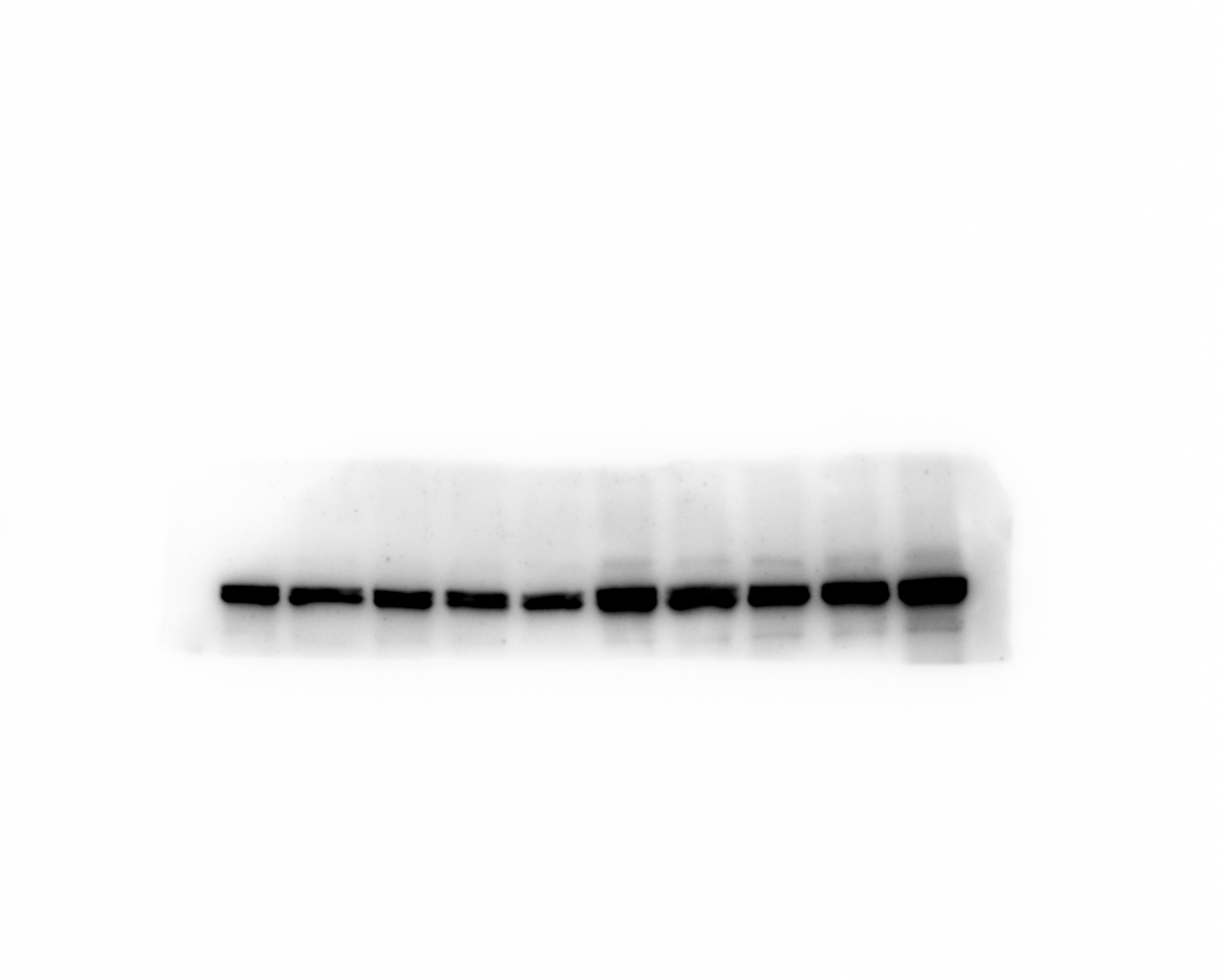

Supplement: Figure 4—source data 2. [file elife-103663-fig4-data2.zip › Figure 4/Figure 4L/Hnf4α.Tif]

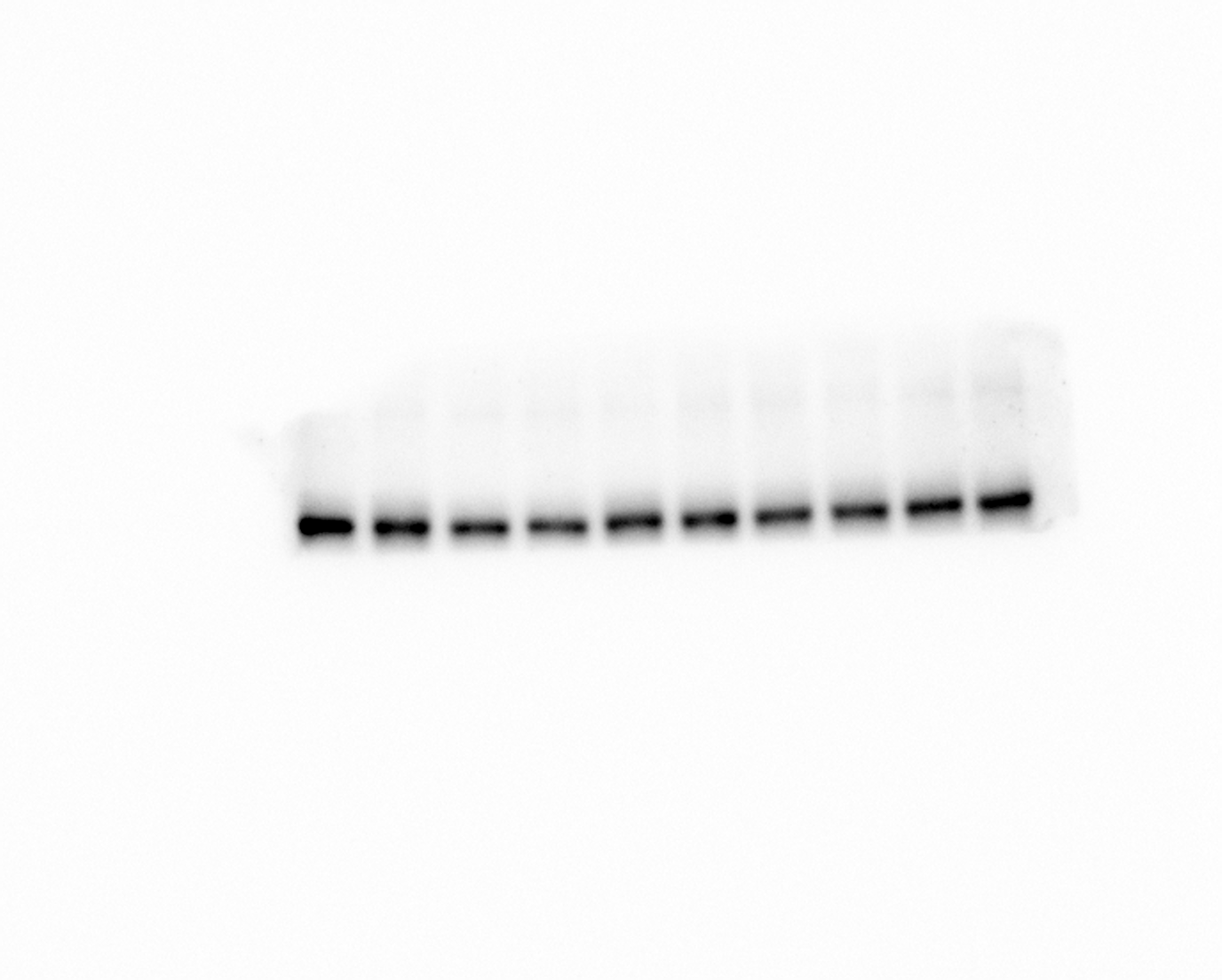

Supplement: Figure 4—source data 2. [file elife-103663-fig4-data2.zip › Figure 4/Figure 4L/Tubulin.Tif]

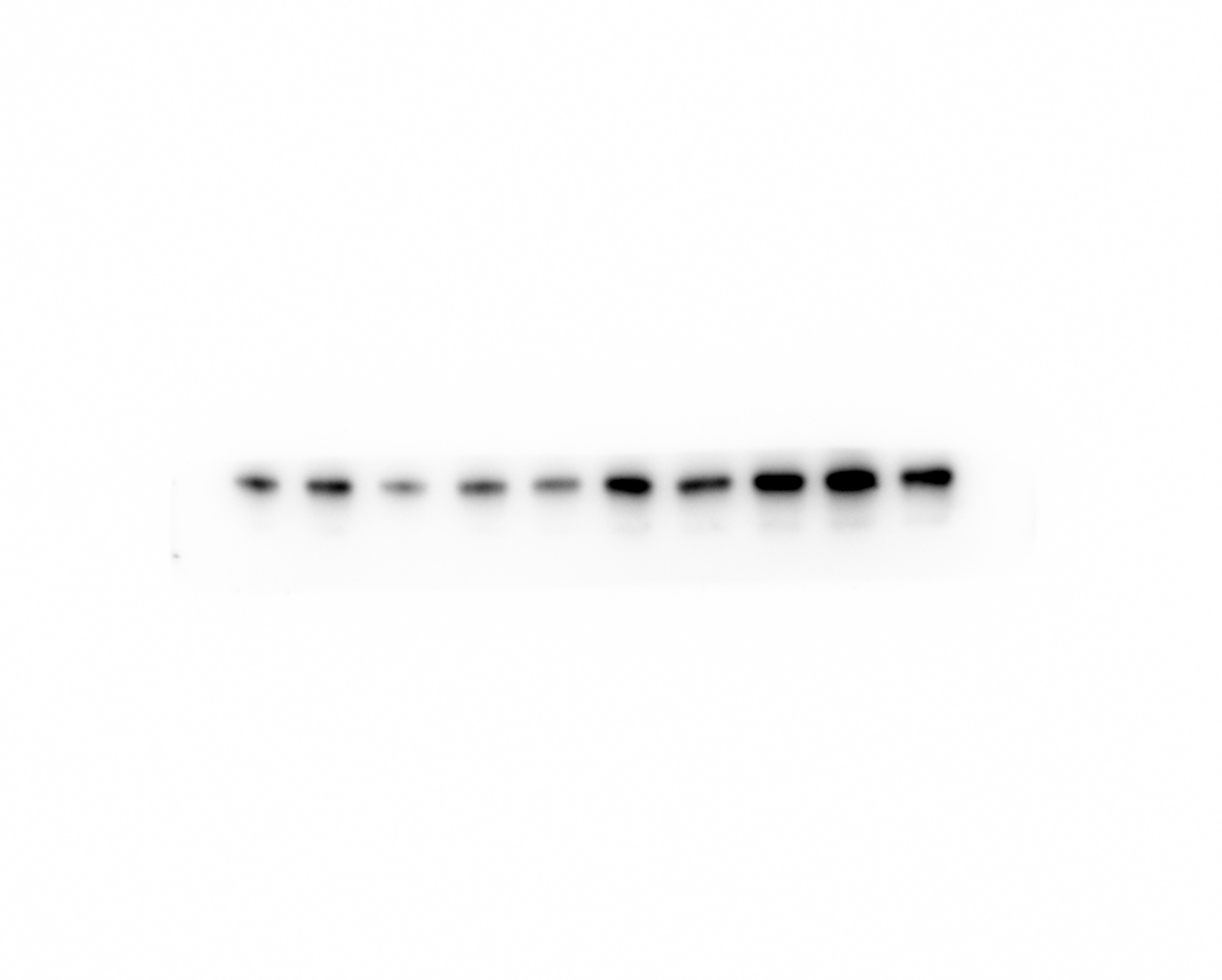

Supplement: Figure 4—source data 2. [file elife-103663-fig4-data2.zip › Figure 4/Figure 4M/Fbp1.Tif]

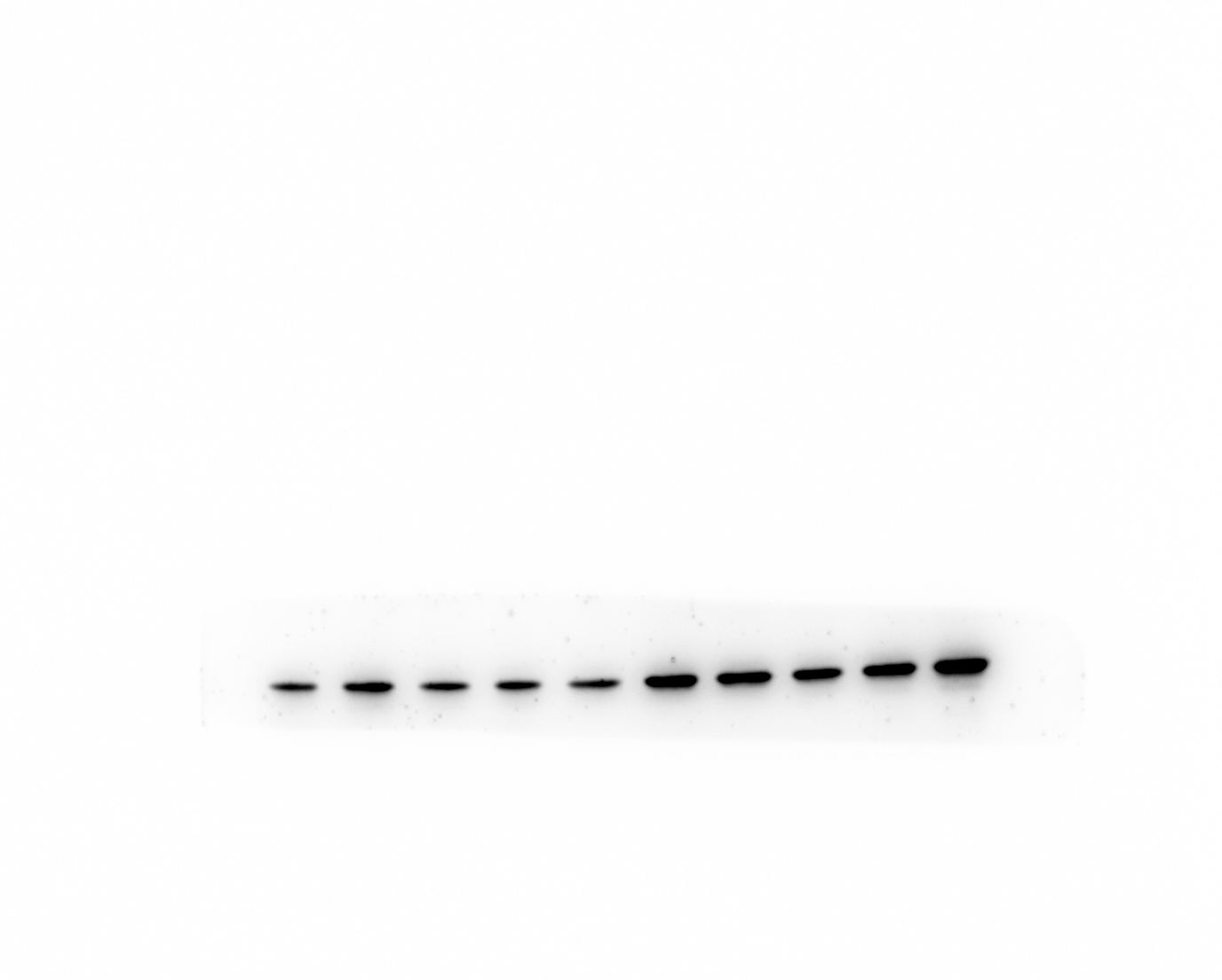

Supplement: Figure 4—source data 2. [file elife-103663-fig4-data2.zip › Figure 4/Figure 4M/Hnf4α.Tif]

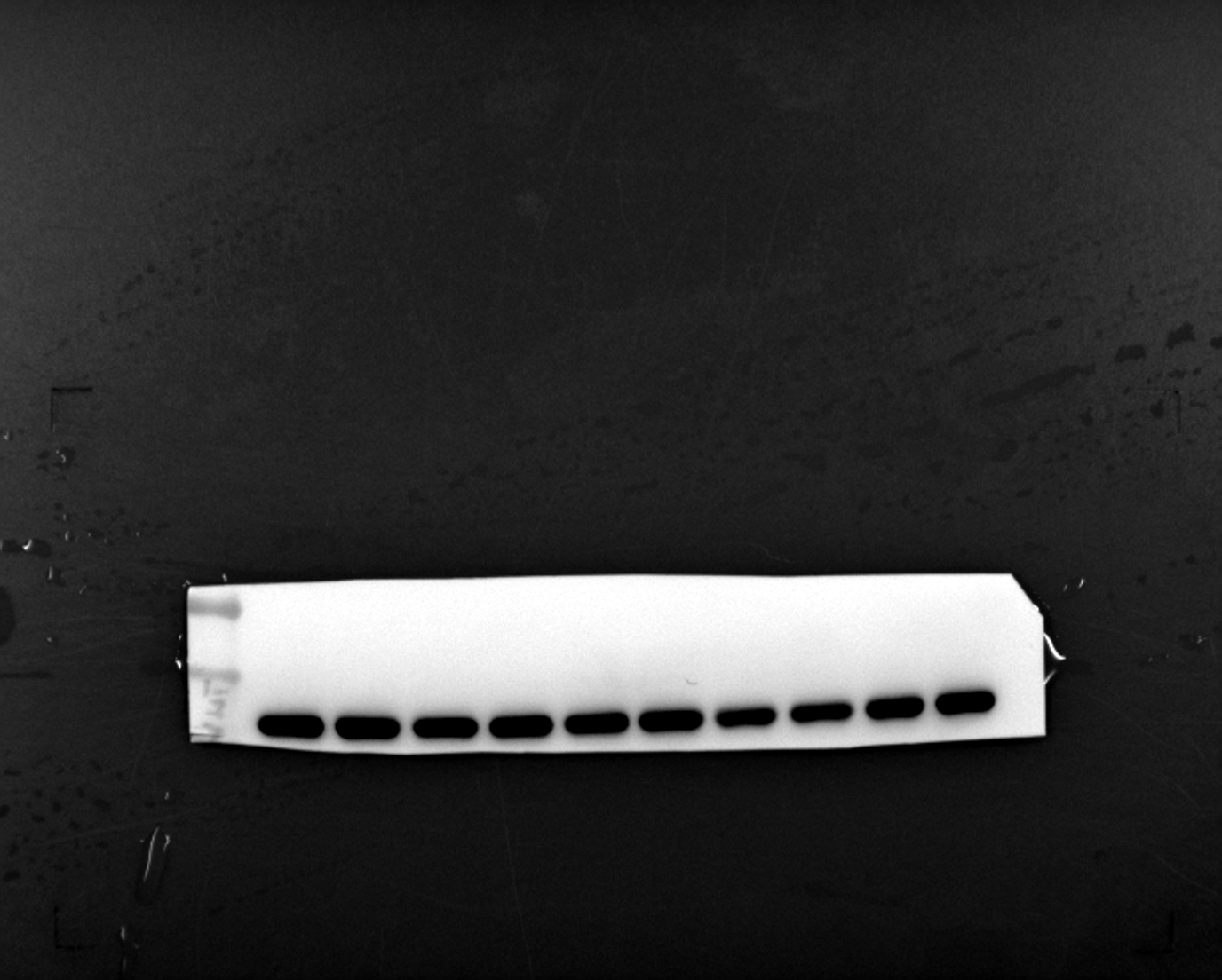

Supplement: Figure 4—source data 2. [file elife-103663-fig4-data2.zip › Figure 4/Figure 4M/Tubulin.Tif]

Figure. 5A

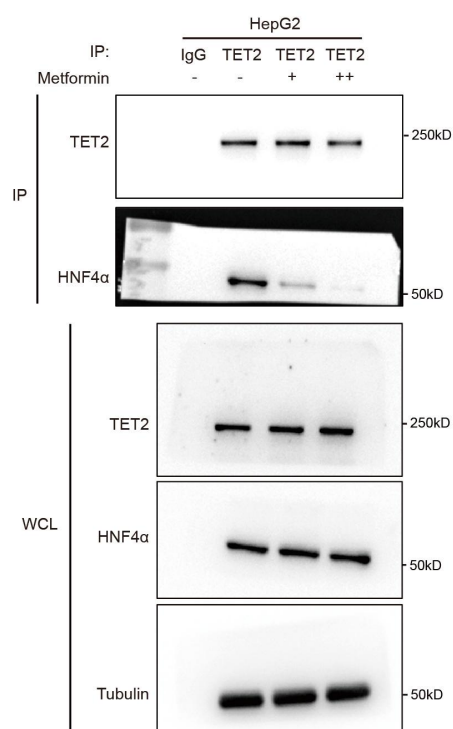

Figure. 5C

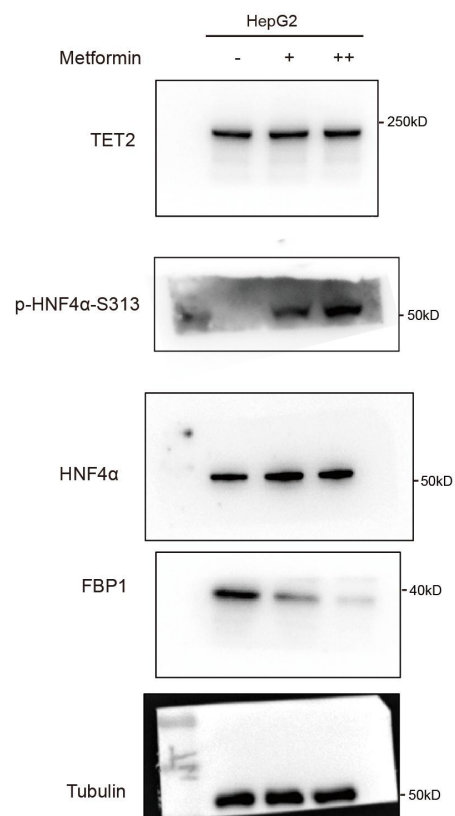

Figure. 5D

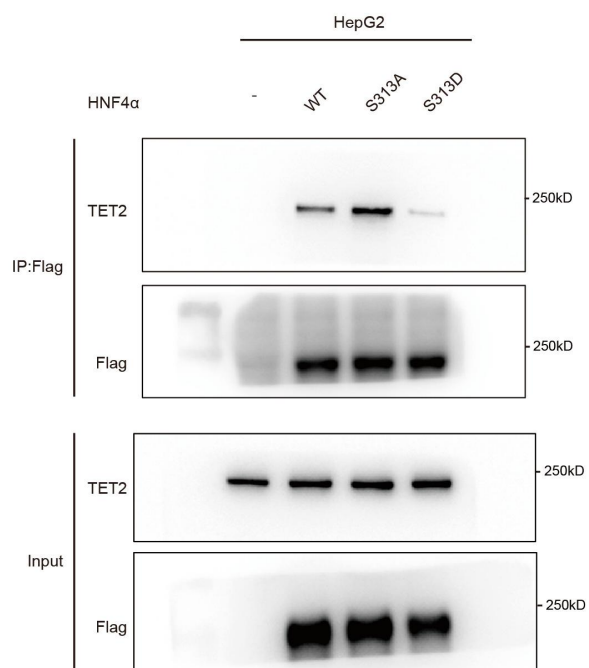

Figure. 5F

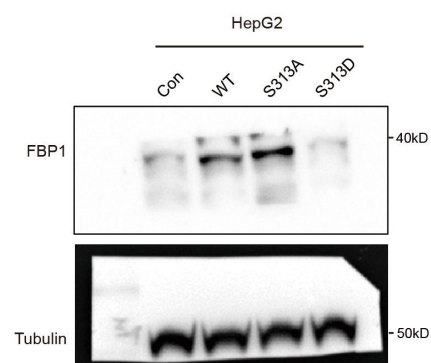

Supplement: Figure 5—source data 1. [file elife-103663-fig5-data1.pdf]

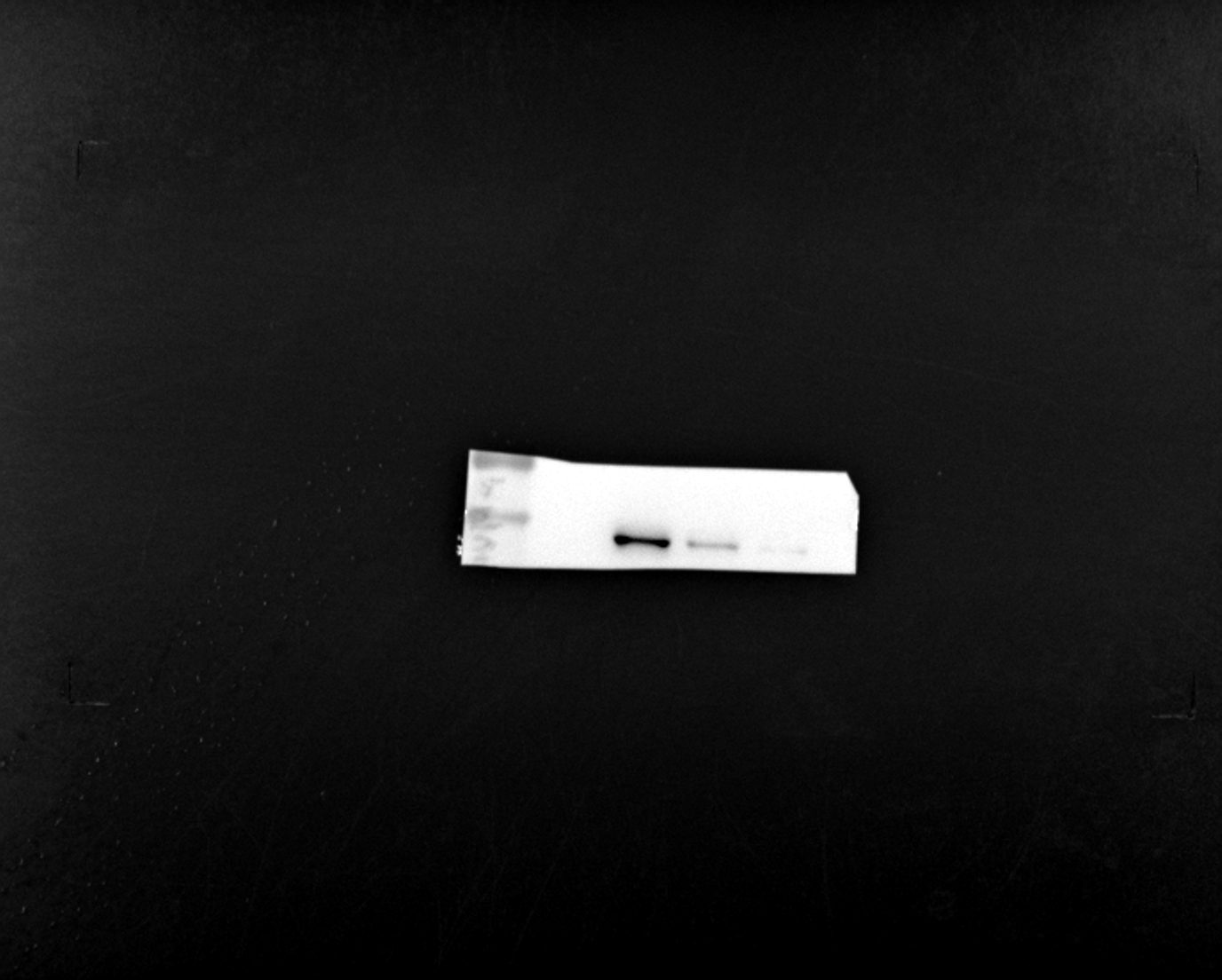

Supplement: Figure 5—source data 2. [file elife-103663-fig5-data2.zip › Figure 5/Figure 5A/IP-HNF4α.Tif]

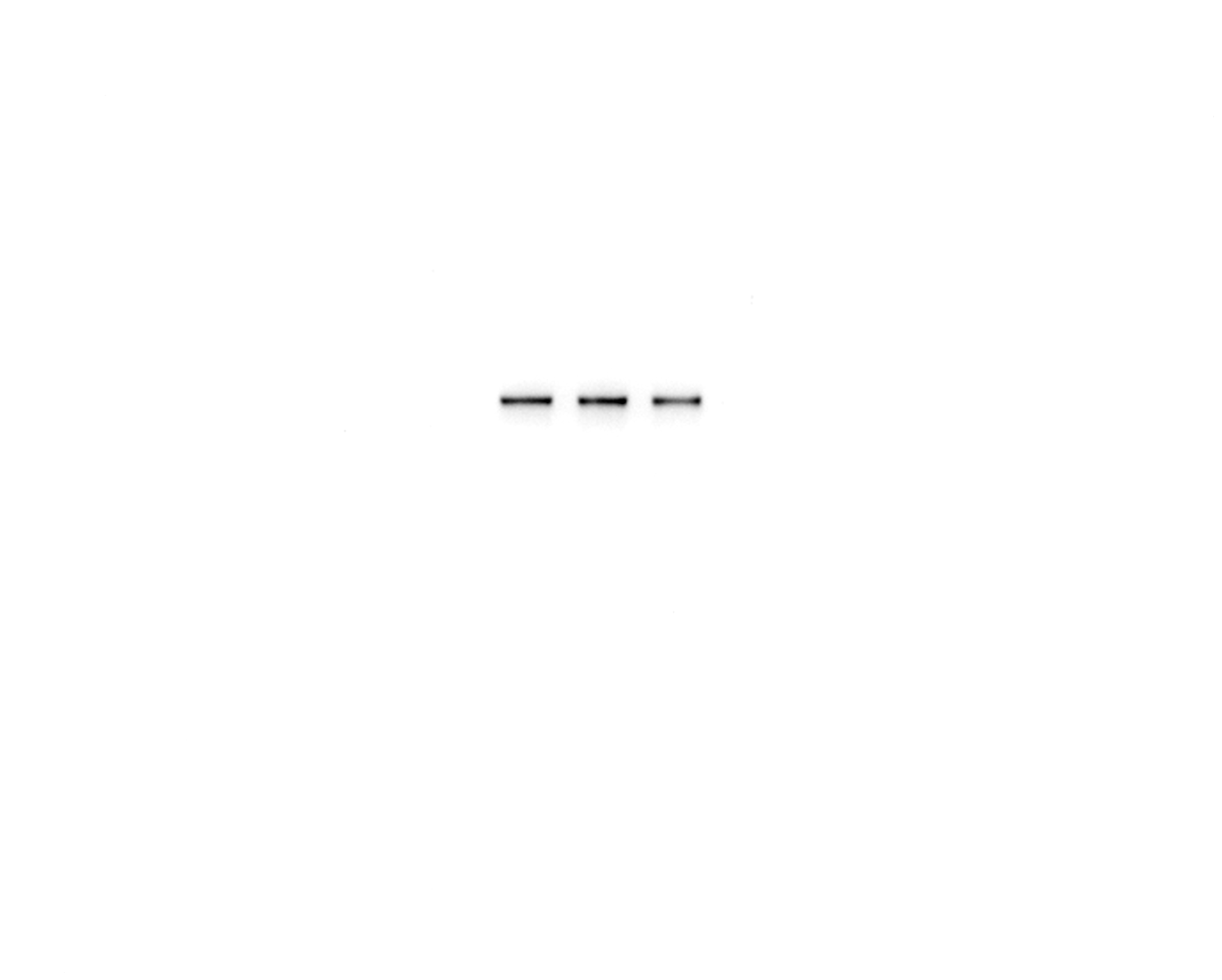

Supplement: Figure 5—source data 2. [file elife-103663-fig5-data2.zip › Figure 5/Figure 5A/IP-TET2.Tif]

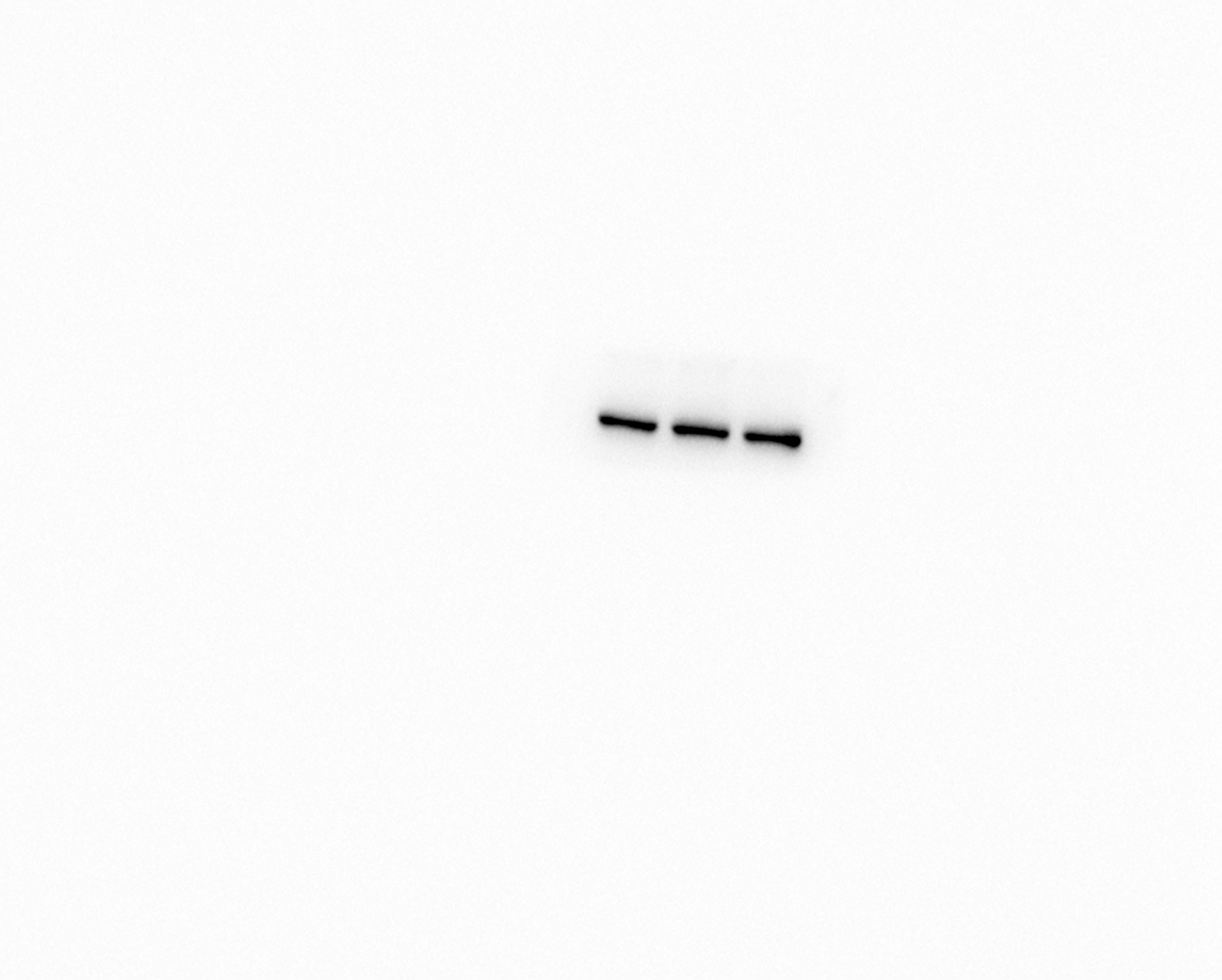

Supplement: Figure 5—source data 2. [file elife-103663-fig5-data2.zip › Figure 5/Figure 5A/Input-HNF4α.Tif]

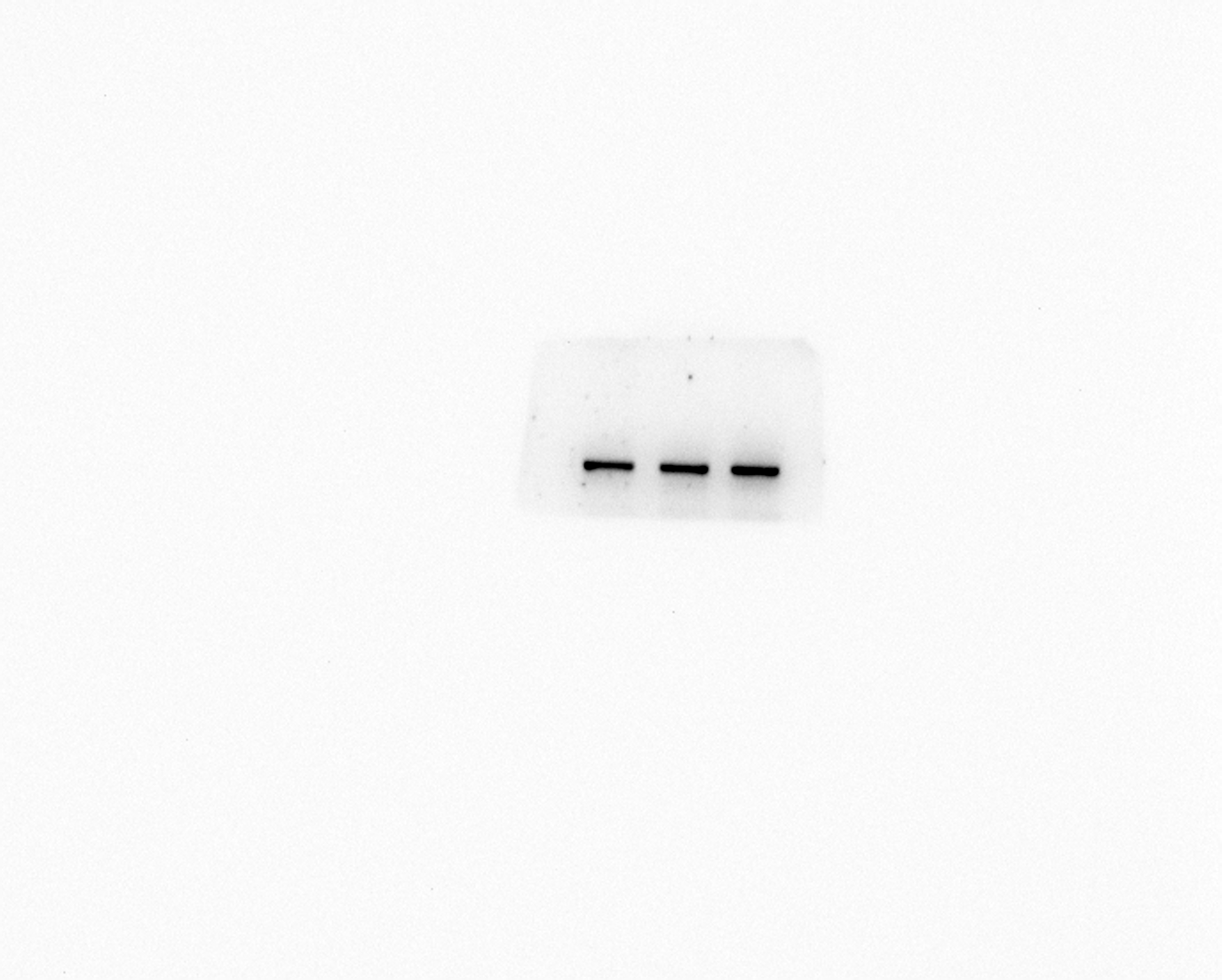

Supplement: Figure 5—source data 2. [file elife-103663-fig5-data2.zip › Figure 5/Figure 5A/Input-TET2.Tif]

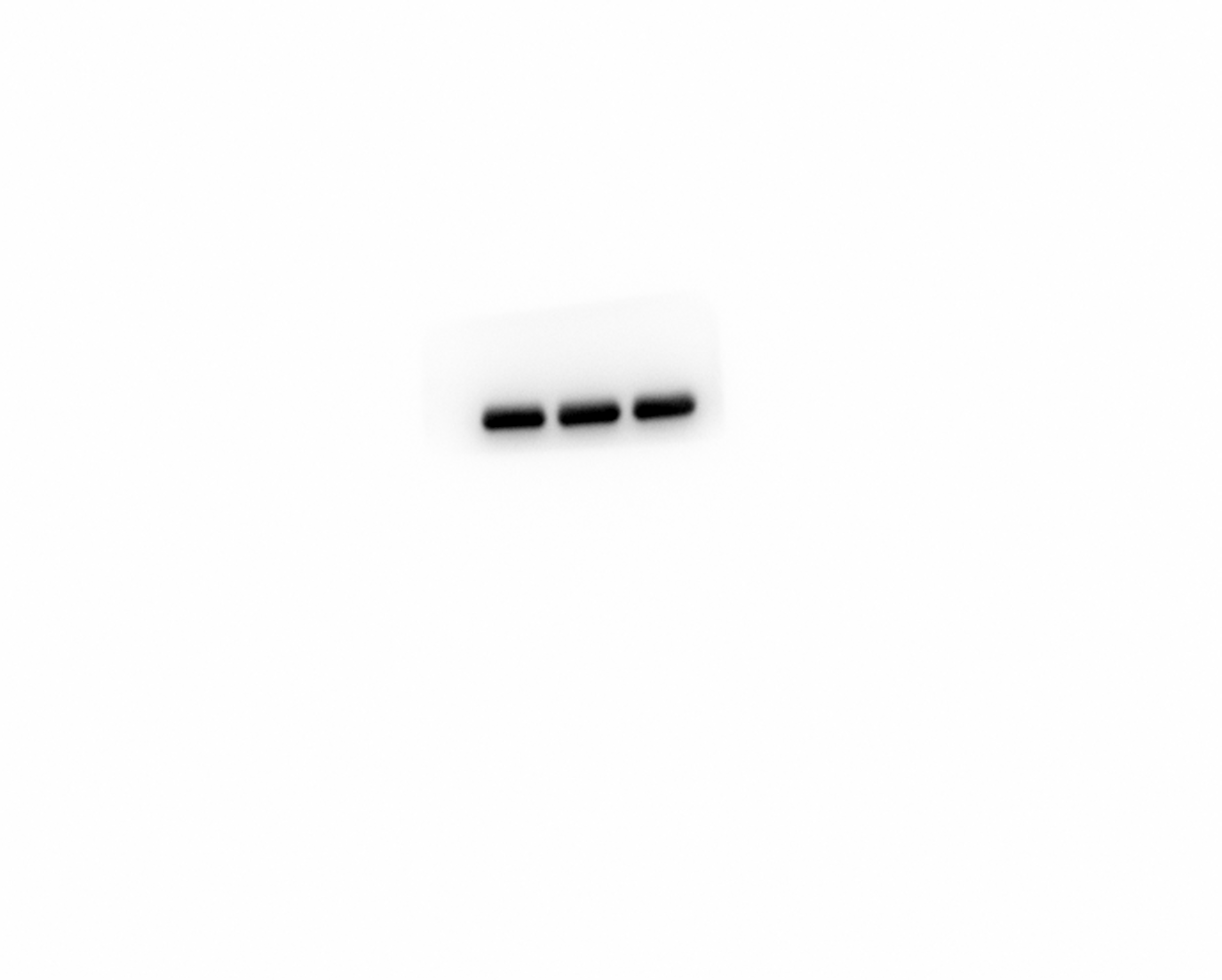

Supplement: Figure 5—source data 2. [file elife-103663-fig5-data2.zip › Figure 5/Figure 5A/Input-Tubulin.Tif]

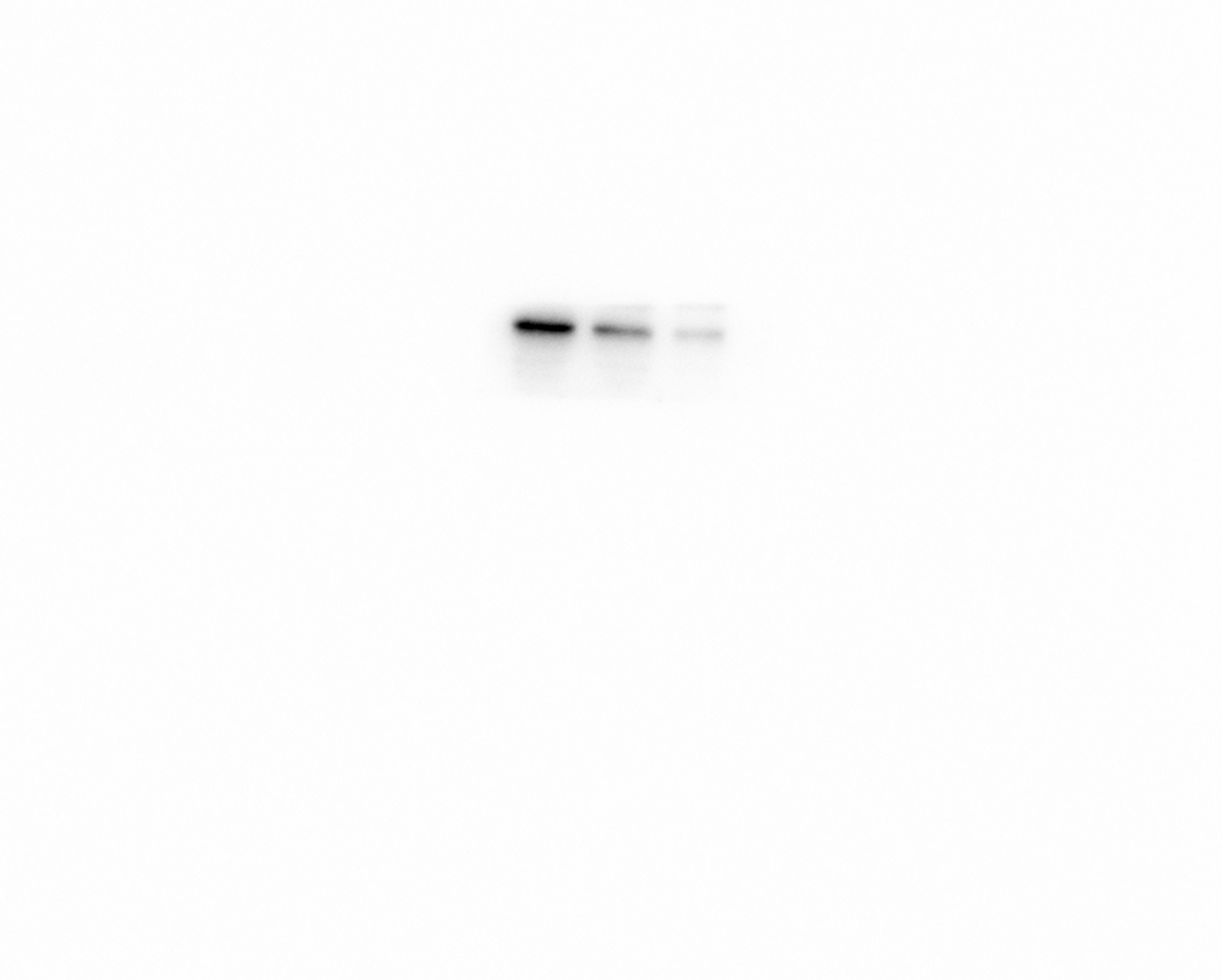

Supplement: Figure 5—source data 2. [file elife-103663-fig5-data2.zip › Figure 5/Figure 5C/FBP1.Tif]

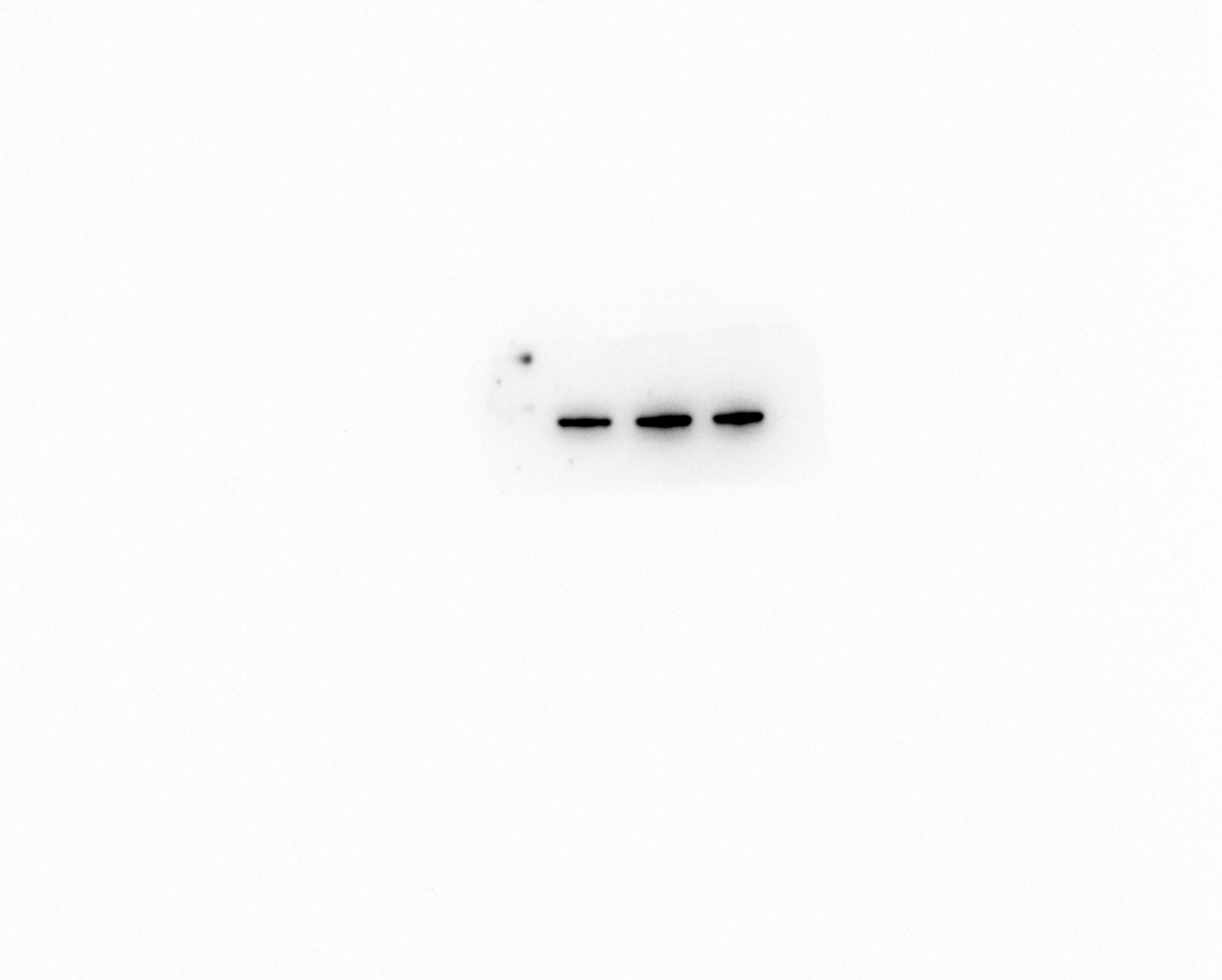

Supplement: Figure 5—source data 2. [file elife-103663-fig5-data2.zip › Figure 5/Figure 5C/HNF4α.Tif]

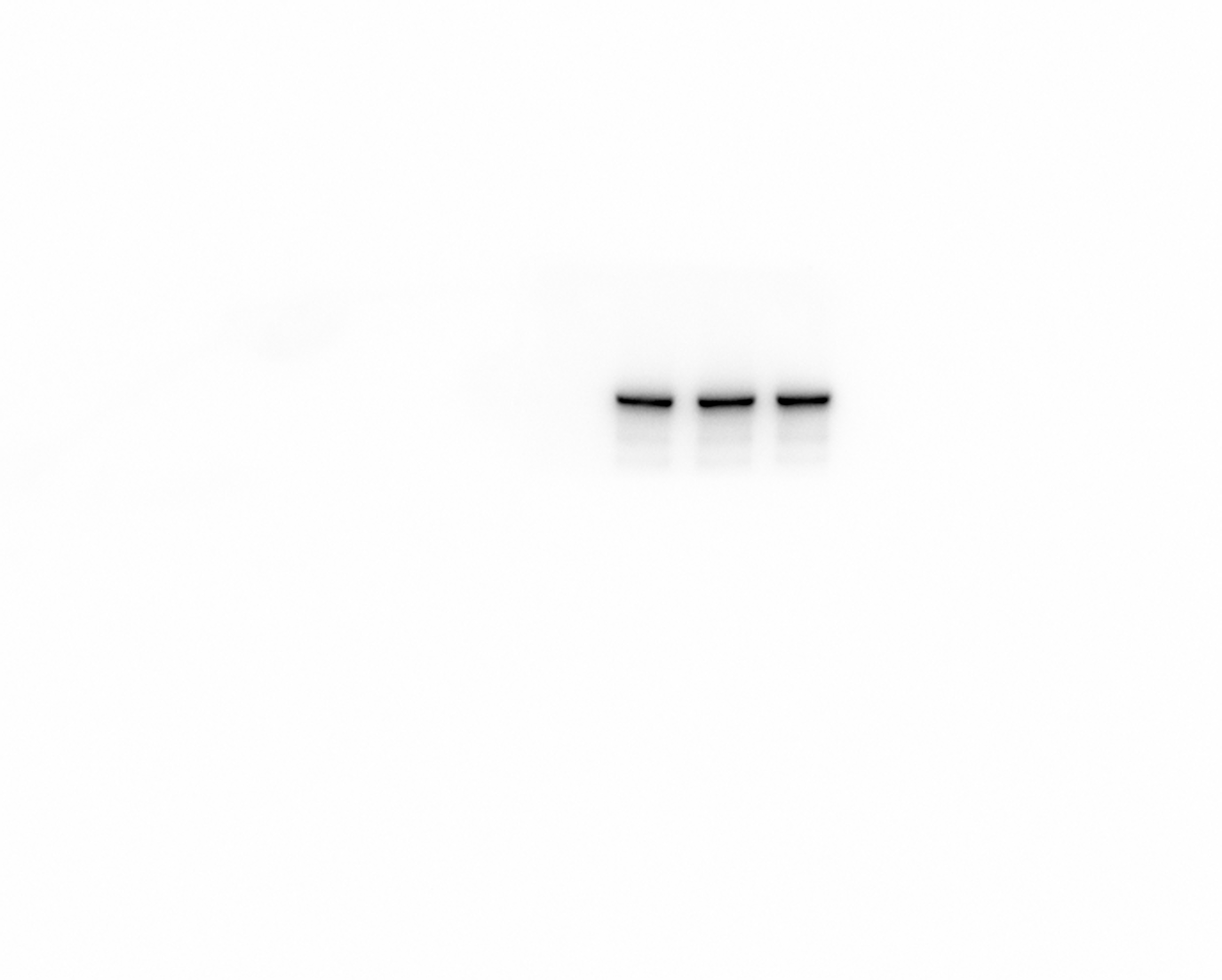

Supplement: Figure 5—source data 2. [file elife-103663-fig5-data2.zip › Figure 5/Figure 5C/TET2.Tif]

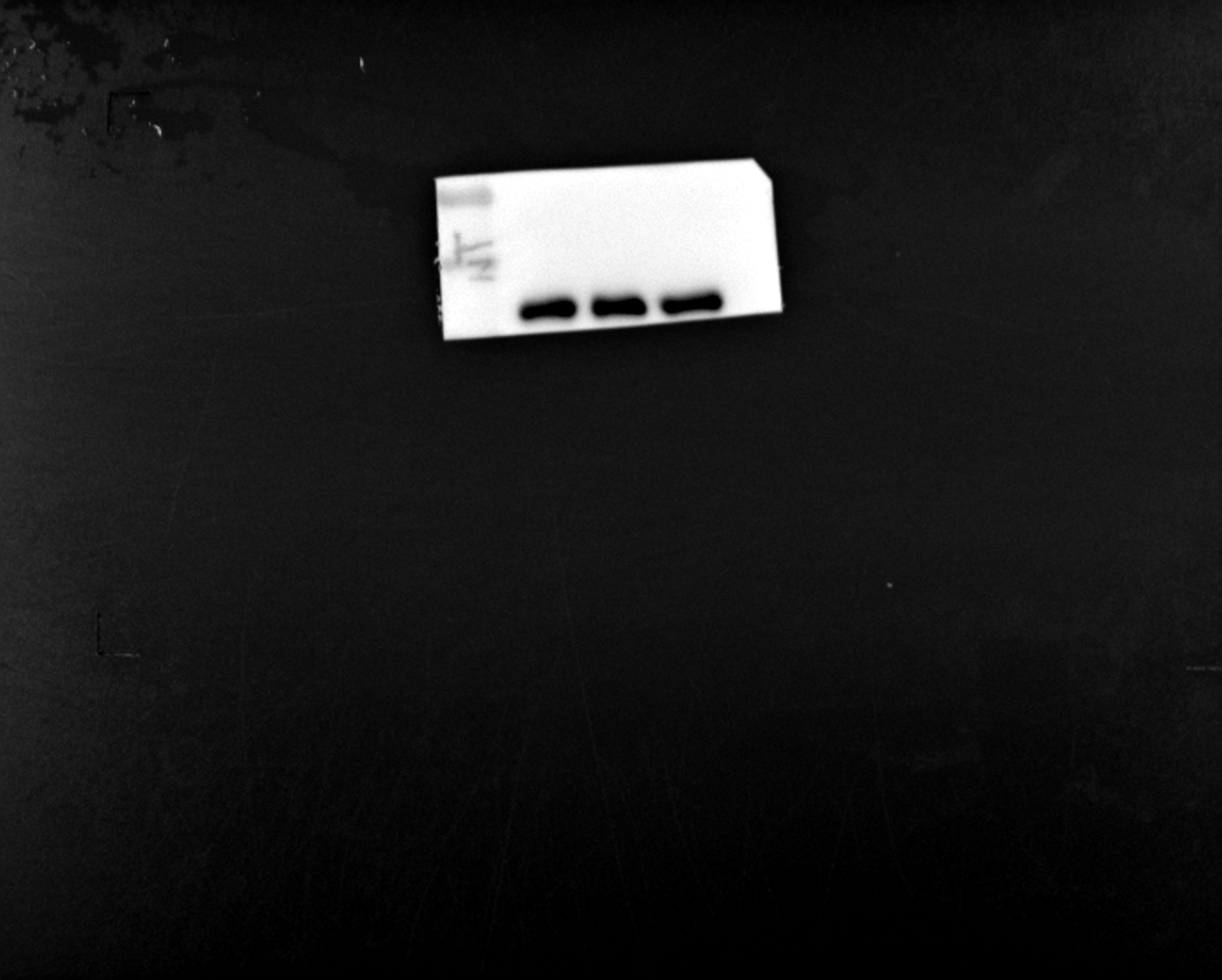

Supplement: Figure 5—source data 2. [file elife-103663-fig5-data2.zip › Figure 5/Figure 5C/Tubulin.Tif]

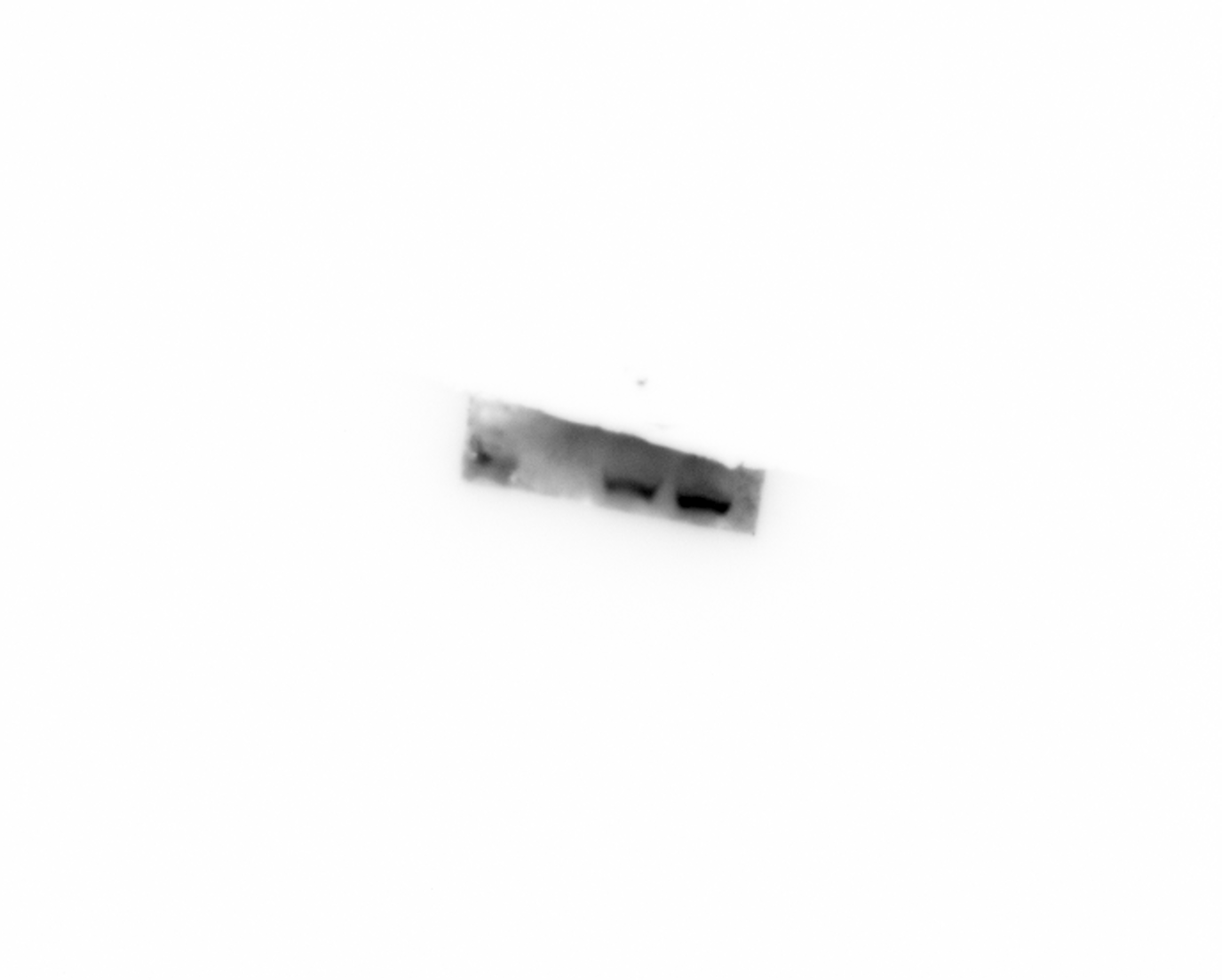

Supplement: Figure 5—source data 2. [file elife-103663-fig5-data2.zip › Figure 5/Figure 5C/p-HNF4α.Tif]

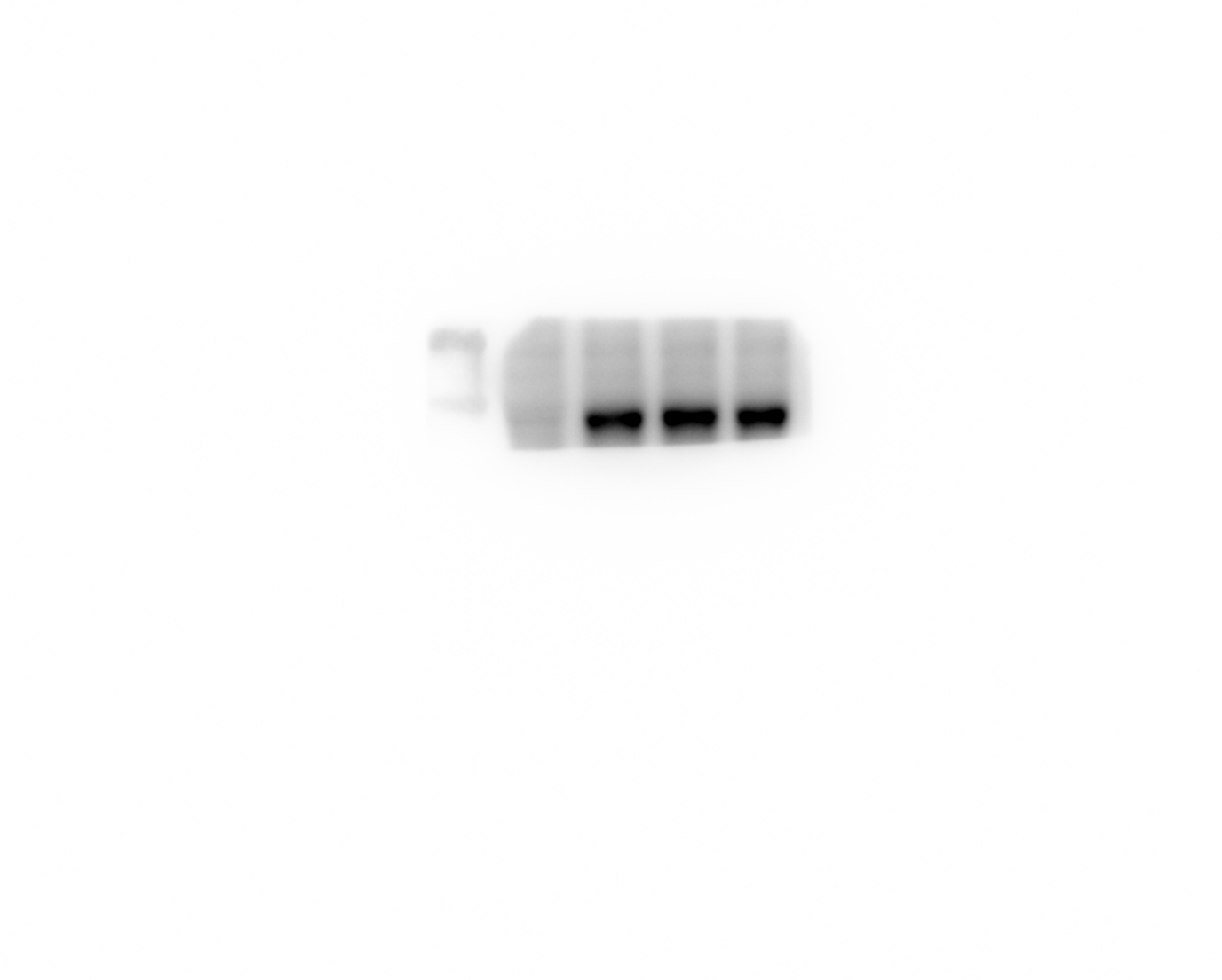

Supplement: Figure 5—source data 2. [file elife-103663-fig5-data2.zip › Figure 5/Figure 5D/IP-Flag.Tif]

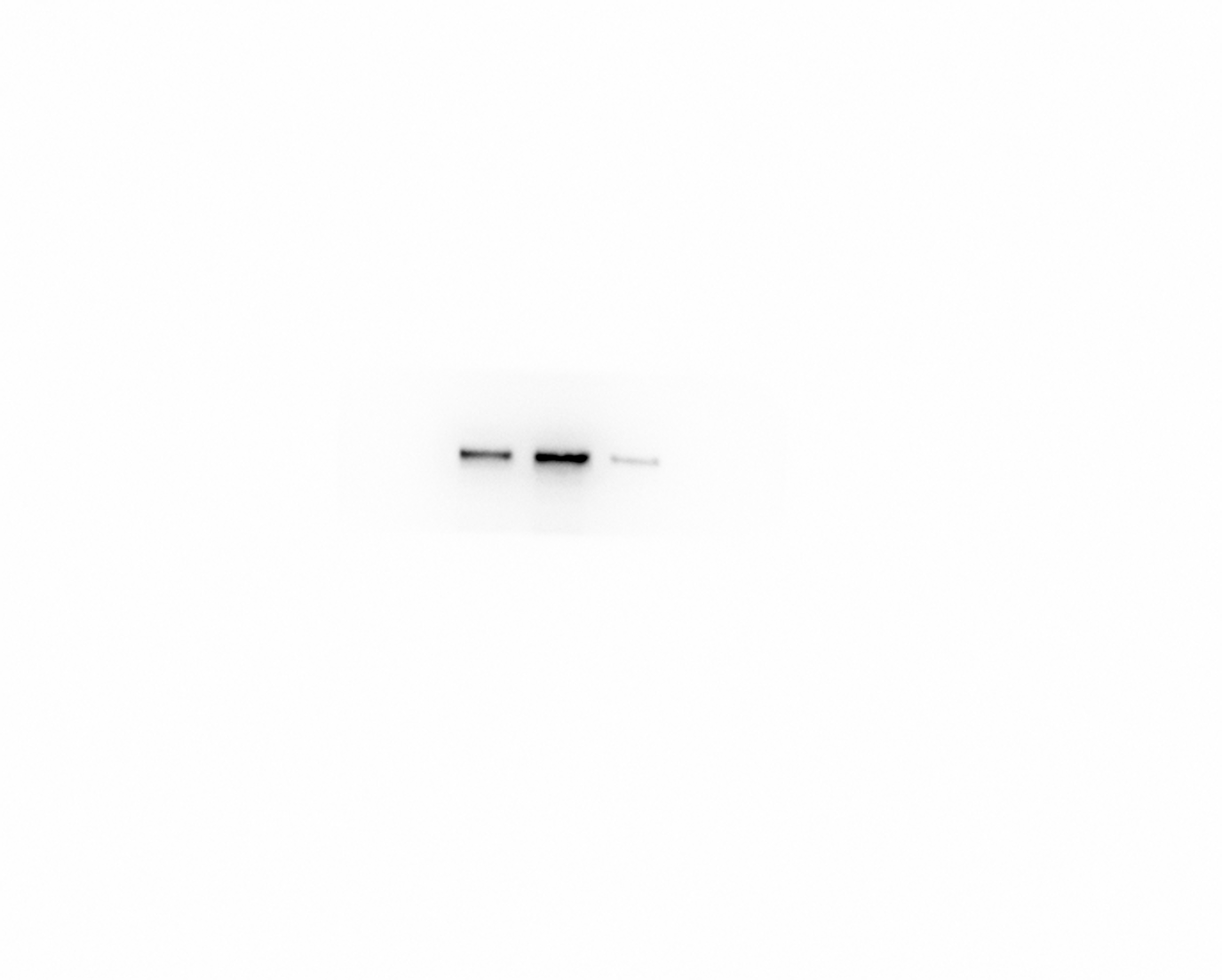

Supplement: Figure 5—source data 2. [file elife-103663-fig5-data2.zip › Figure 5/Figure 5D/IP-TET2.Tif]

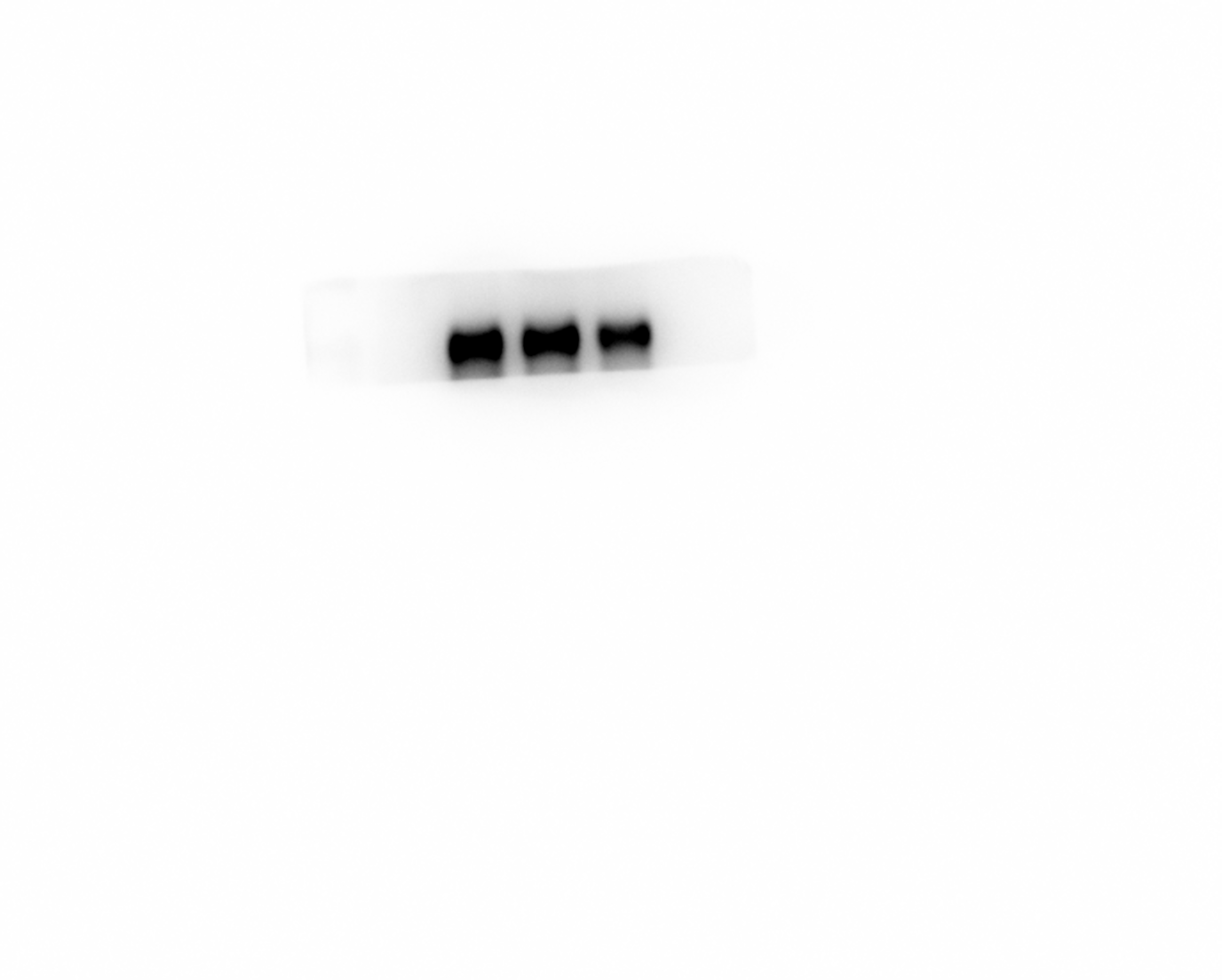

Supplement: Figure 5—source data 2. [file elife-103663-fig5-data2.zip › Figure 5/Figure 5D/Input-Fag.Tif]

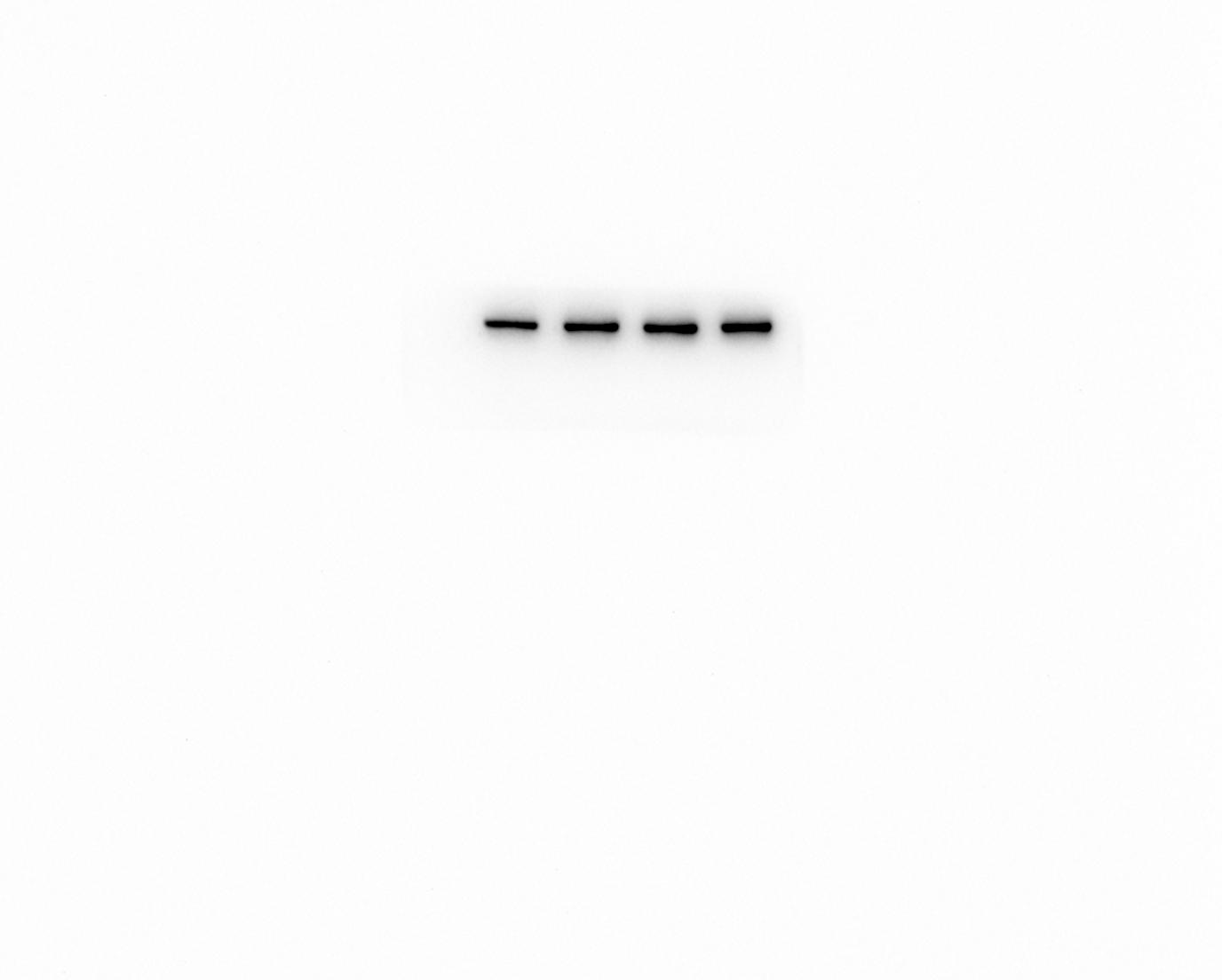

Supplement: Figure 5—source data 2. [file elife-103663-fig5-data2.zip › Figure 5/Figure 5D/Input-TET2.Tif]

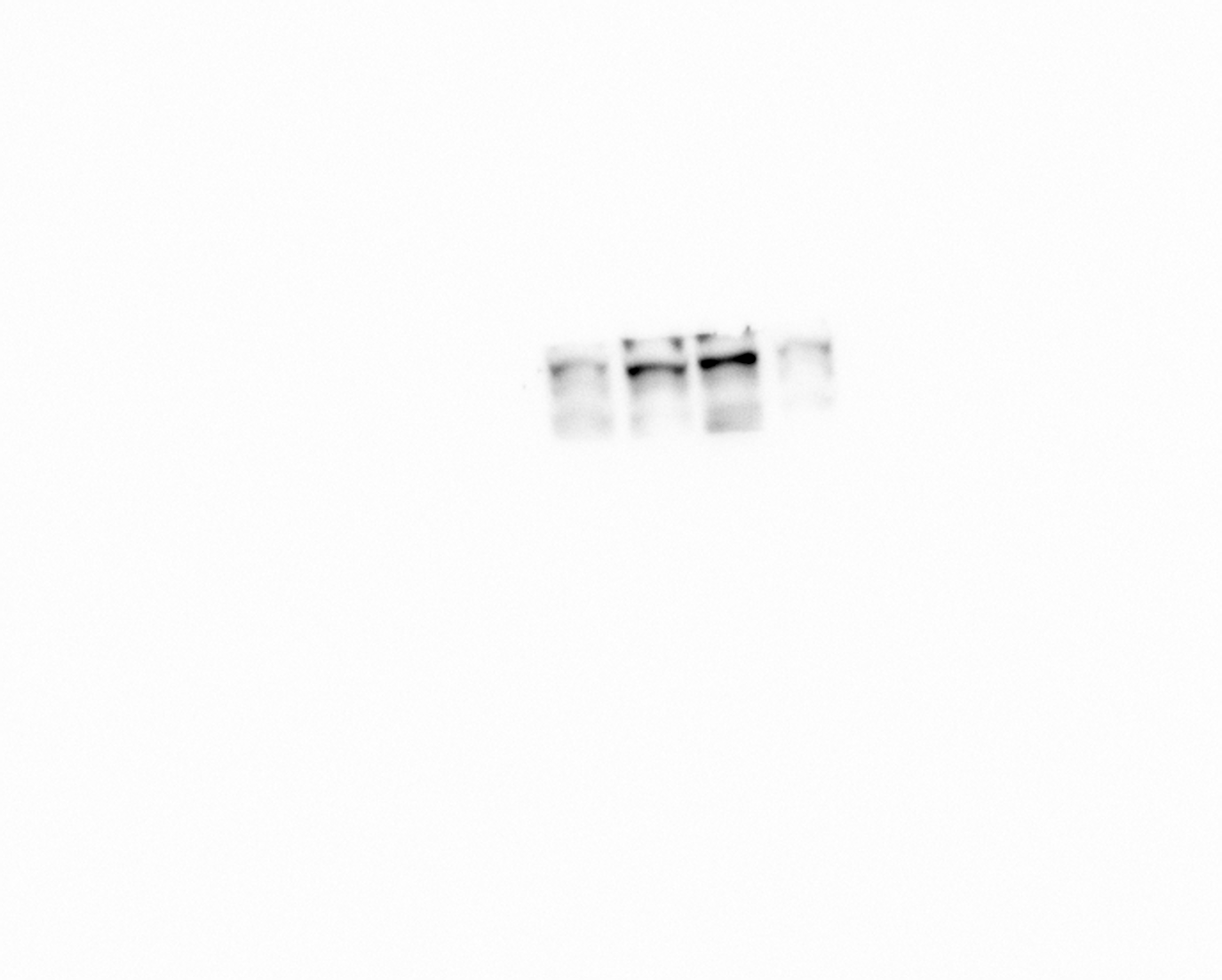

Supplement: Figure 5—source data 2. [file elife-103663-fig5-data2.zip › Figure 5/Figure 5F/FBP1.Tif]

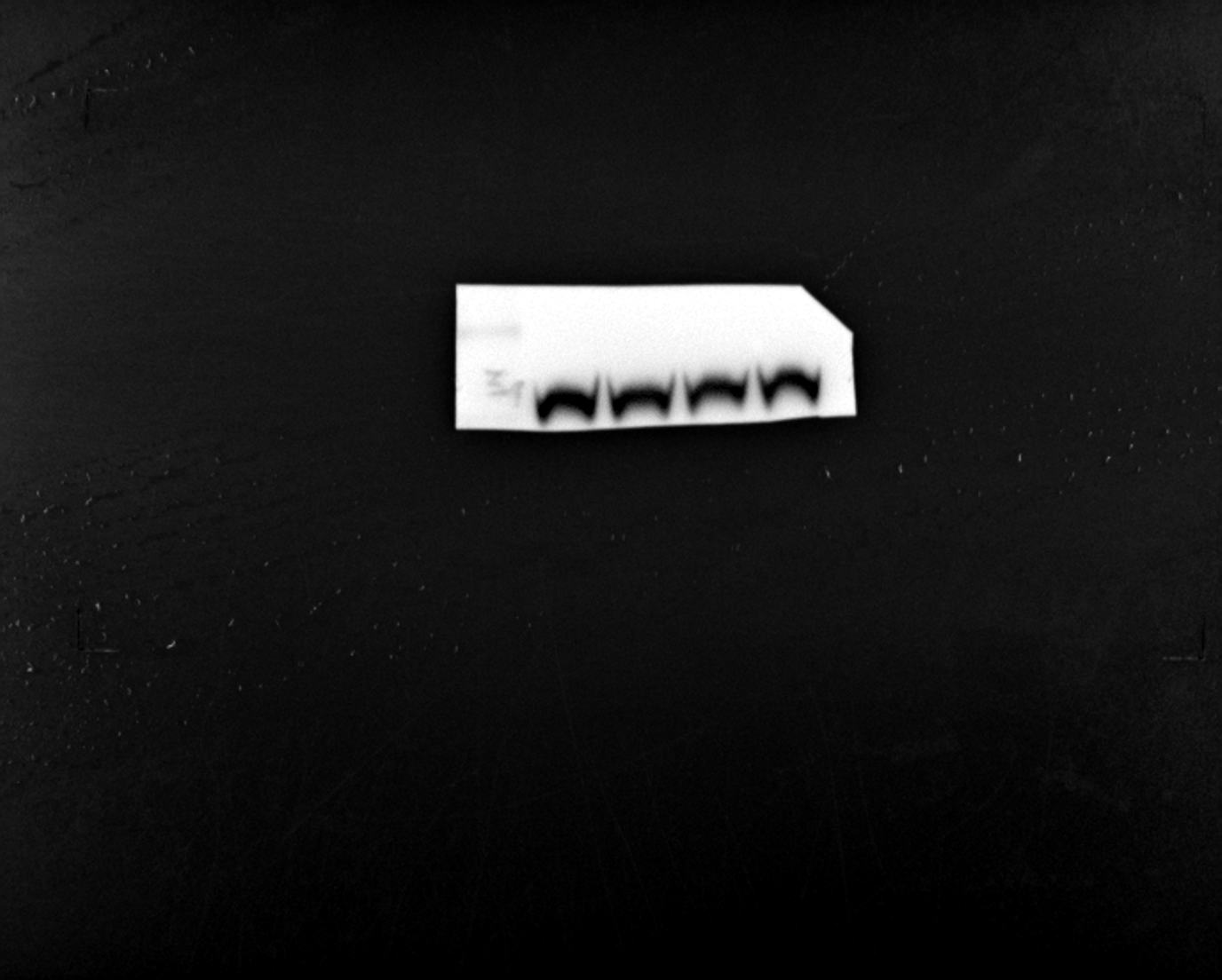

Supplement: Figure 5—source data 2. [file elife-103663-fig5-data2.zip › Figure 5/Figure 5F/Tubulin.Tif]

Figure. 6E

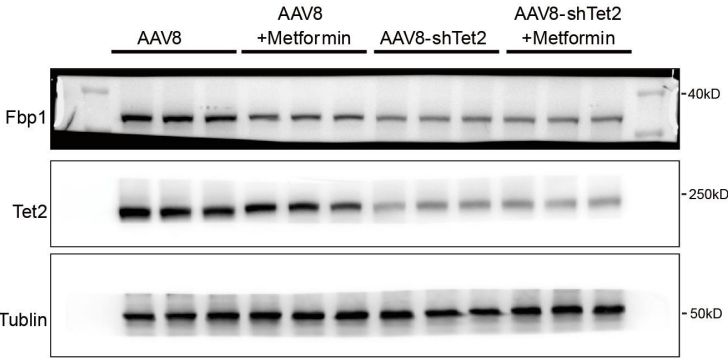

Supplement: Figure 6—source data 1. [file elife-103663-fig6-data1.pdf]

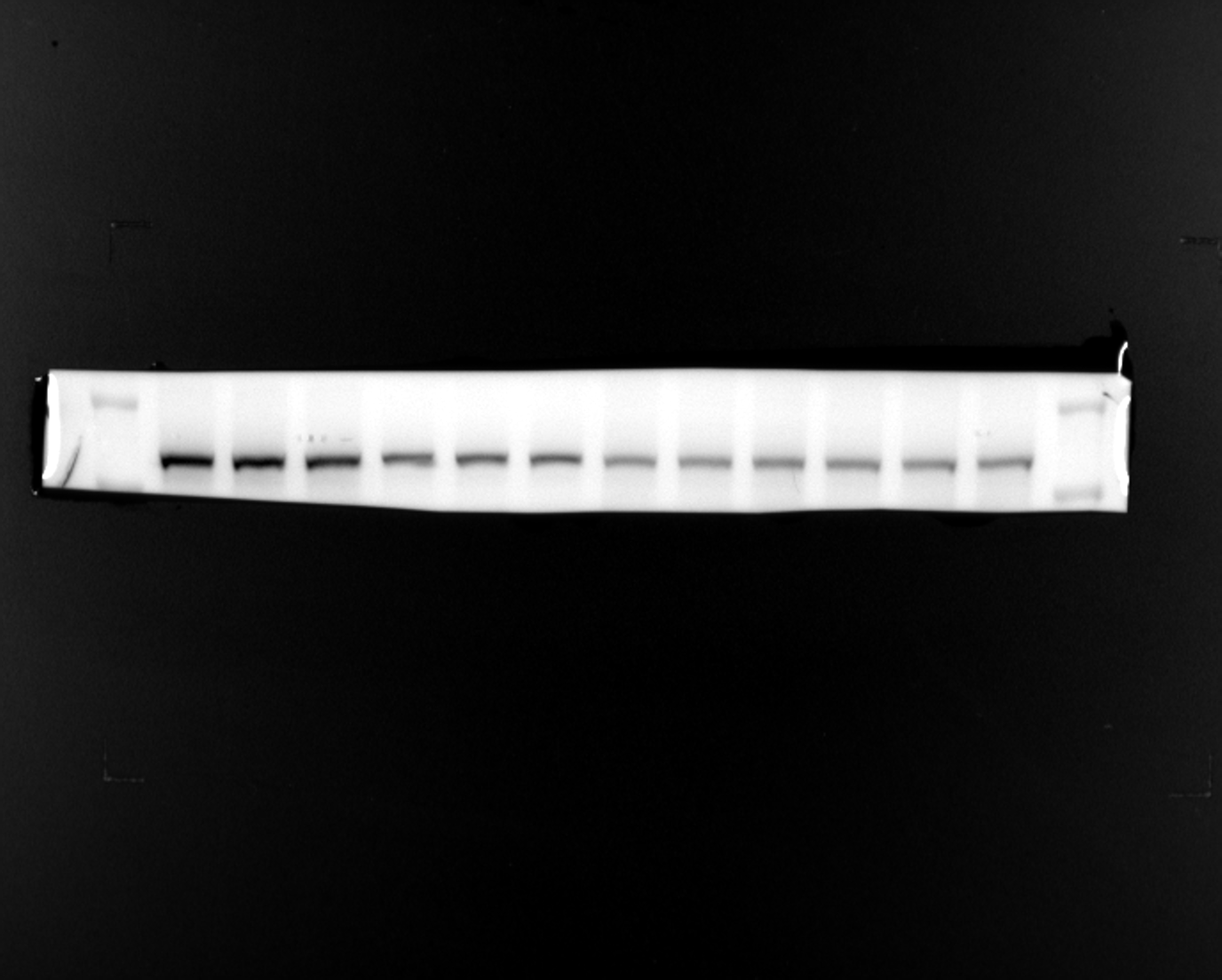

Supplement: Figure 6—source data 2. [file elife-103663-fig6-data2.zip › Figure 6/Fbp1.Tif]

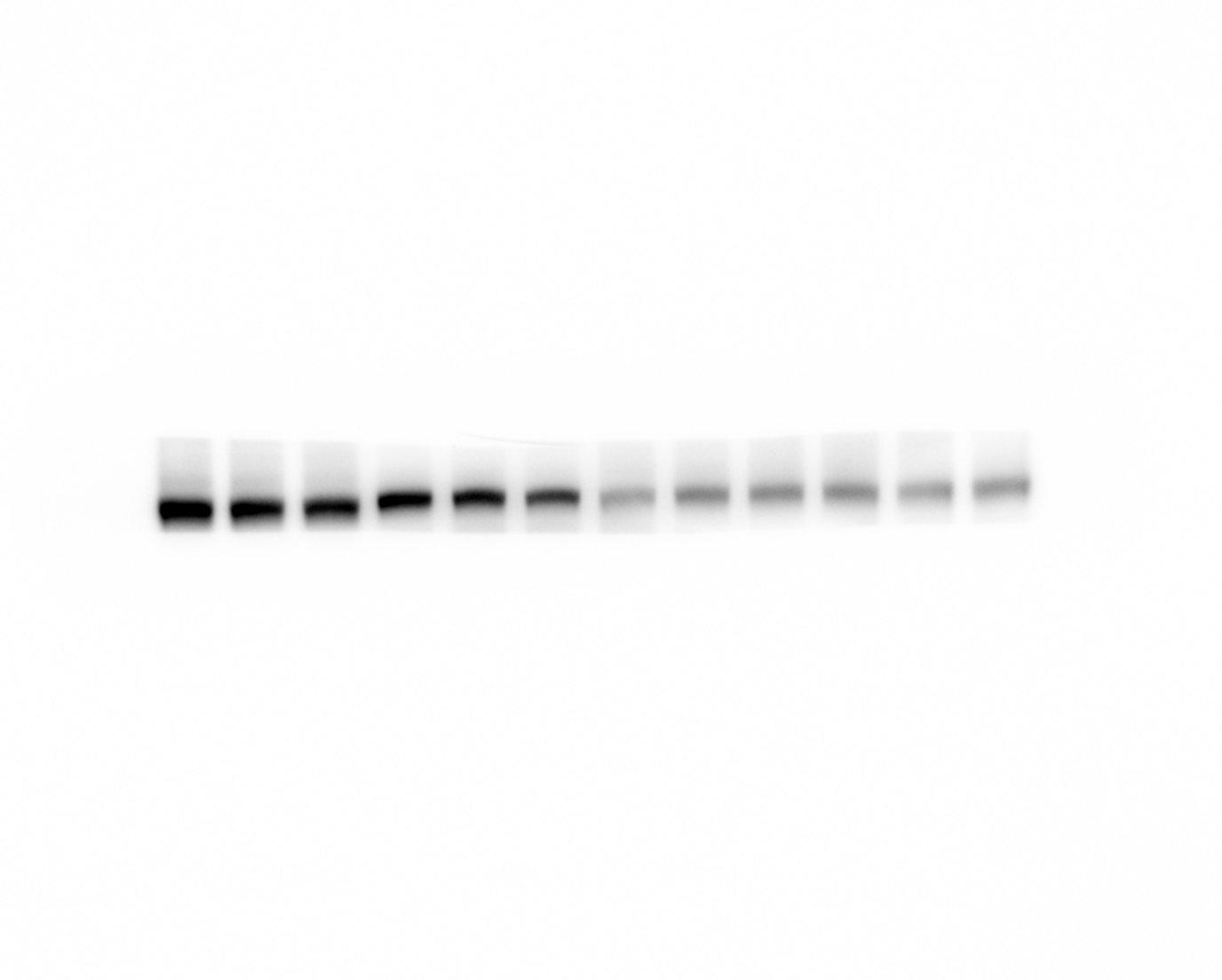

Supplement: Figure 6—source data 2. [file elife-103663-fig6-data2.zip › Figure 6/Tet2.Tif]

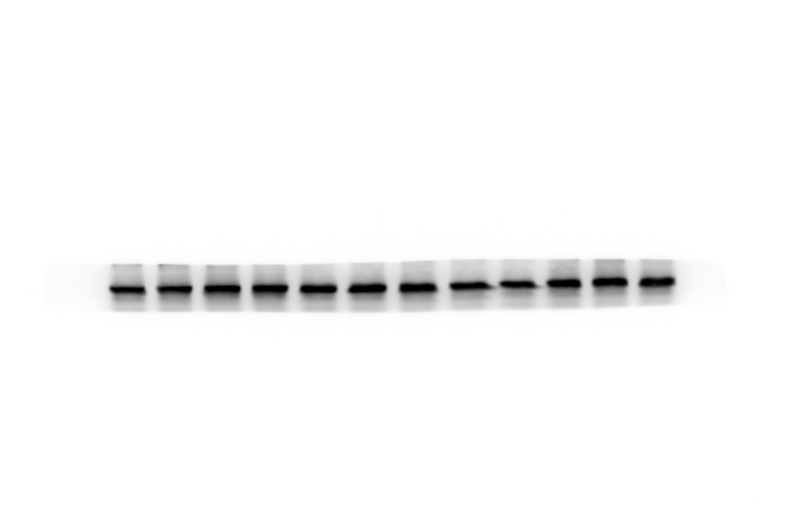

Supplement: Figure 6—source data 2. [file elife-103663-fig6-data2.zip › Figure 6/Tubulin.jpg]
